# 2D Conjugated Metal–Organic Frameworks as Electrocatalysts for Boosting Glycerol Upgrading Coupled with Hydrogen Production

**DOI:** 10.1002/anie.202502425

**Published:** 2025-05-08

**Authors:** Yutong Luo, Michael Beerbaum, Stefan Röher, Sara Amanzadeh Salout, Volodymyr Bon, Yang Lu, Leonid Shupletsov, Ankita De, Xinliang Feng, Inez M. Weidinger, Thomas D. Kühne, Arafat Hossain Khan, Irena Senkovska, Stefan Kaskel

**Affiliations:** ^1^ Chair of Inorganic Chemistry I Technische Universität Dresden Bergstraße 66 01069 Dresden Germany; ^2^ Center for Advanced Systems Understanding and Helmholtz‐Zentrum Dresden‐Rossendorf Technische Universität Dresden Untermarkt 20 D‐02826 Görlitz Germany; ^3^ Chair of Electrochemistry Technische Universität Dresden Zellescher Weg 19 01069 Dresden Germany; ^4^ Chair of Chemistry Technische Universität Dresden Bergstraße 66 01069 Dresden Germany; ^5^ Center for Advancing Electronics Dresden Technische Universität Dresden 01067 Dresden Germany; ^6^ Max‐Planck‐Institute of Microstructure Physics Weinberg 2 06120 Halle Germany

**Keywords:** 2D conjugated MOFs, Active centres, Electrocatalysis, Glycerol conversion, Reaction mechanism

## Abstract

The valorisation of glycerol using renewable electricity is recognised as an effective and attractive approach for producing high‐value compounds from biomass byproducts. 2D conjugated metal–organic frameworks (2D c‐MOFs) have emerged as promising electrocatalysts due to their tunable structures, high electronic conductivity, and efficient utilisation of well‐defined active centres. In this study, we report the first systematic investigation of 2D c‐MOFs containing Ni‐X_4_ (X = O or N) moieties for the glycerol oxidation reaction (GOR), examining key factors influencing both electrocatalytic activity and selectivity. In situ ^13^C electrochemical nuclear magnetic resonance and Raman spectroscopies provide insights into the GOR mechanism and confirm that Ni–O_4_ sites are the primary active centres. Theoretical calculations further reveal that [Ni_3_(HHTQ)_2_]_n_ (HHTQ = 2,3,7,8,12,13‐hexahydroxytricycloquinazoline) exhibits superior GOR activity due to strong adsorption of reaction intermediates and weak interlayer interactions. This work highlights the potential of 2D c‐MOFs as highly efficient GOR catalysts, paving the way for the rational design of advanced electrocatalysts and contributing to the development of sustainable energy conversion and storage technologies.

## Graphical abstract

The 2D conjugated [Ni_3_(HHTQ)_2_]_n_ MOF (HHTQ = 2,3,7,8,12,13‐hexahydroxytricycloquinazoline) with Ni–O_4_ node can be simultaneously utilised as a catalyst for glycerol oxidation reaction and hydrogen evolution reaction, showing high catalytic activity and selectivity. The in situ measurements and theoretical calculations reveal the reaction mechanism, active centres, and origin of outstanding performance.

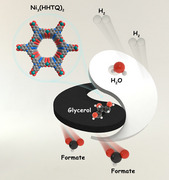

## Introduction

With the escalating depletion of finite fossil resources, increasing greenhouse gas emissions, and the emerging global warming scenario, the increasing demand for carbon neutrality and energy sustainability has accelerated the development of hydrogen technology.^[^ ^1^, ^2^, ^3^ ^]^ In addition to the well‐established electrolytic water splitting, electrocatalytic biomass valorisation, especially electroreforming of glycerol, is emerging as an effective route to convert glycerol into value‐added organic compounds and to replace the sluggish oxygen evolution reaction (OER), facilitating cathodic hydrogen evolution reaction (HER).^[^ ^4^, ^5^ ^]^ Glycerol is one of the most significant platform chemicals for biomass valorisation, produced in large quantities as a low‐value byproduct from the biodiesel manufacturing industry.^[^ ^6^ ^]^ It has been proven that the electrochemical glycerol oxidation reaction (GOR) can produce a broad family of chemicals with high economic values, such as glyceraldehyde, glyceric acid, glycolic acid and formic acid.^[^ ^7^, ^8^, ^9^, ^10^, ^11^ ^]^ Among these GOR products, formic acid or formate is an important and highly valued industrial intermediate and can be widely utilised across the textile, leather, medical, and pesticide industries.^[^ ^12^, ^13^, ^14^ ^]^ Additionally, it has generated considerable interest in the fields of hydrogen storage and fuel cells as potential liquid hydrogen carrier because of the relatively high theoretical gravimetric and volumetric hydrogen capacity (4.4 wt% and 53.4 g L^−1^, respectively).^[^ ^15^, ^16^ ^]^ On the other hand, the theoretical redox potential for the conversion of glycerol to formate requires only 0.69 V (versus standard hydrogen electrode, SHE), which is much lower than 1.23 V (vs.SHE) needed for OER, making it thermodynamically more favourable.^[^ ^17^ ^]^ Therefore, coupling the electrooxidation of glycerol to formate with the simultaneous electrolysis of water for hydrogen production would be an attractive and promising technology, which can not only lower the required input of cell voltage towards hydrogen generation at the cathode but also produce upgraded formic acid with the consumption of low‐valued glycerol at the anode.

Unfortunately, up to now, the development of practical and efficient electrolytic systems for synchronous glycerol valorisation and hydrogen generation remains highly challenging, primarily due to the need for electrocatalysts that combine low cost, high activity, high selectivity, and robust stability for both the GOR and HER.

Enhancing the intrinsic activity of individual active sites and increasing the density of exposed active sites are the most important approaches for the rational design of highly efficient electrocatalysts towards advanced renewable energy conversion technologies.^[^ ^18^, ^19^ ^]^ Two‐dimensional conjugated metal–organic frameworks (2D c‐MOFs), assembled by π‐conjugated non‐innocent organic ligands with metals, have attracted widespread attention, holding enormous potential for applications in electrocatalysis due to their catalytic active centres, layered periodic structure, large surface area, and chemical stability.^[^ ^20^, ^21^, ^22^ ^]^ In general, the square‐planar secondary building units (SBUs) of 2D c‐MOFs are widely regarded as the catalytically active centres (denoted as M‐X_4_, M = Ni, Co, Cu, etc.; X = O, N, S, etc.) and determine the intrinsic electrocatalytic properties of 2D c‐MOF catalysts.^[^ ^23^, ^24^ ^]^ The c‐MOFs can be subdivided into three categories, based on the coordinating functional groups and metal ions involved: semiquinoid‐based materials with M‐O_4_ active sites, iminosemiquinoid‐based materials with M‐N_4_ active sites, and dithiolene‐based materials with M‐S_4_ active sites, respectively. The 2D c‐MOFs are layered structures with strong in‐plane d–π conjugations and compact out‐of‐plane π–π interactions, realising the highly effective transfer of charge carriers along the 2D plane and π‐stacking direction, thus overcoming the insulating nature of conventional three‐dimensional (3D) MOFs and accelerating the kinetics of electron transfer during catalytic reaction process.^[^ ^25^, ^26^, ^27^ ^]^ Benefiting from these compelling features, 2D c‐MOFs have demonstrated remarkable electrocatalytic performance, among others, for water splitting,^[^ ^28^, ^29^, ^30^ ^]^ oxygen reduction reaction (ORR),^[^ ^31^, ^32^, ^33^ ^]^ 2e^−^ ORR for H_2_O_2_ synthesis,^[^ ^34^, ^35^, ^36^ ^]^ carbon dioxide reduction reaction (CO_2_RR),^[^ ^37^, ^38^, ^39^ ^]^ and nitrogen reduction reaction (NRR).^[^ ^40^, ^41^ ^]^ Unfortunately, due to the complex reaction process of GOR itself, reports on the application of MOFs towards GOR are extremely rare and mainly involve 3D MOFs. Recently, a yttrium‐doped [Co_2_(bdc)_2_(dabco)]*_n_* MOF (bdc = 1,4‐benzenedicarboxylate, and dabco = 1,4‐diazabicyclo[2.2.2]octane), has been proven as an effective GOR catalyst for electro‐reforming of glycerol in an alkaline environment.^[^ ^42^ ^]^ We anticipate that 2D c‐MOFs with M‐X_4_ active sites can serve as efficient catalysts for hydrogen production alongside formate synthesis. Therefore, we aim to investigate their catalytic activity and elucidate the underlying reaction mechanisms.

**Figure 1:**
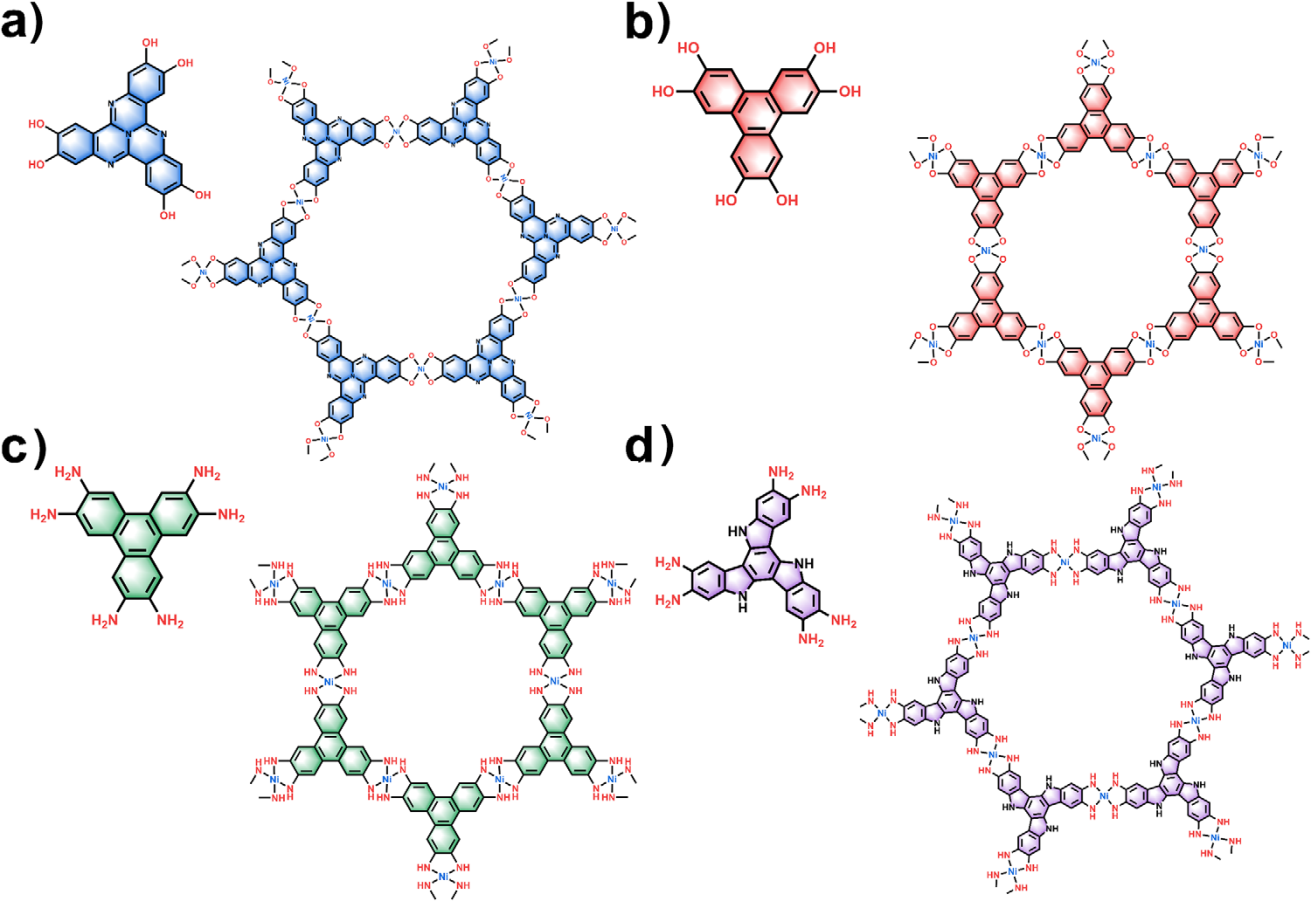
Chemical structures of the ligands and corresponding 2D c‐MOFs: a) Ni_3_(HHTQ)_2_, b) Ni_3_(HHTP)_2_, c) Ni_3_(HITP)_2,_ and d) Ni_3_(HATI)_2_.

In the following, we explore 2D c‐MOFs with Ni‐X_4_ (X = O or N) active centres as catalysts for simultaneous water electrolysis at the cathode to produce hydrogen and glycerol consumption at the anode to generate formate. Four kinds of Ni‐based 2D c‐MOFs differing in ligand structure are targeted (Figure 1), namely, [Ni_3_(HHTQ)_2_]*_n_* (HHTQ = 2,3,7,8,12,13‐hexahydroxytricycloquinazoline) and [Ni_3_(HHTP)_2_]*_n_* (HHTP = 2,3,6,7,10,11‐hexahydroxytriphenylene) with Ni‐O_4_ active sites, [Ni_3_(HITP)_2_]*_n_* (HITP = 2,3,6,7,10,11‐hexaiminotriphenylene) and [Ni_3_(HATI)_2_]*_n_* (HATI = 2,3,7,8,12,13‐hexaiminotriindole) with Ni‐N_4_ active sites, respectively. The GOR performance of these 2D c‐MOFs is evaluated under alkaline conditions. Interestingly, Ni_3_(HHTQ)_2_ with Ni‐O_4_ active centres has the highest activity for GOR. Specifically speaking, the 2D framework has an excellent electrocatalytic performance, reaching a current density of 10 mA cm^−2^ for electro‐oxidation of glycerol at a potential as low as 1.36 V (vs. reversible hydrogen electrode, RHE), as well as a high faradaic efficiency of 90% for formate production at 1.50 V. In addition, Ni_3_(HHTQ)_2_ catalyst integrated into a two‐electrode alkaline electrolytic cell as anode and cathode, demonstrates a low cell voltage of 1.87 V at a current density of 10 mA cm^−2^, which is 194 mV lower than that demanded for electrolytic water splitting. Moreover, with the help of in situ ^13^C electrochemical (EC)–nuclear magnetic resonance (NMR) spectroscopy, the relationship between selectivity towards intermediate products and oxidation potentials is elaborated, and the reaction mechanism of GOR on Ni_3_(HHTQ)_2_ is analysed at the molecular level. In situ EC–Raman spectroscopy further confirms the Ni‐O_4_ sites as the active centres during GOR process. Density functional theory (DFT) exploration demonstrates that the high GOR activity of Ni_3_(HHTQ)_2_ originates from strong adsorption of key molecules and weak interlayer interactions, which enhance the transport and, therefore, the overall kinetic of the process. Our work highlights the enormous potential of 2D c‐MOFs as catalysts with superior activity and high selectivity for the production of value‐added products at the cathode and anode simultaneously, providing insightful guidance into the rational design and advanced research for efficient GOR catalysts, which is of great significance for the development of renewable energy technology.

## Results and Discussion

### MOFs Synthesis and Materials Characterisation

**Figure 2:**
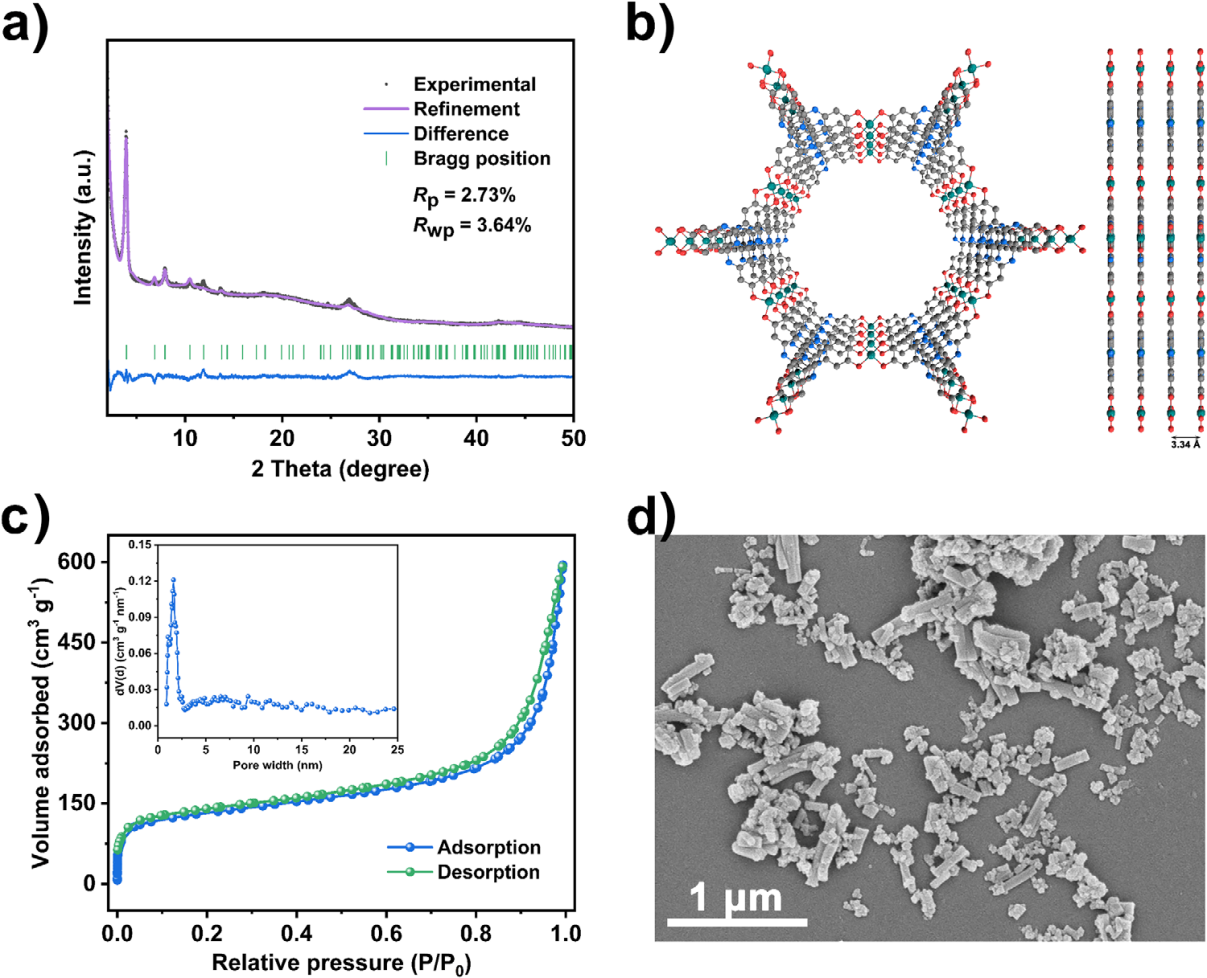
a) Rietveld plot for Ni_3_(HHTQ)_2_, (*R*_p_ = 2.73% and *R*_wp_ = 3.64%). b) Crystal structure of Ni_3_(HHTQ)_2_ along the *c* axis. Red, grey, blue, and green spheres represent O, C, N, and Ni atoms, respectively. Hydrogen atoms are omitted for clarity. c) N_2_ adsorption–desorption isotherms of Ni_3_(HHTQ)_2_ at 77 K, inset: the pore size distribution. d) SEM image of Ni_3_(HHTQ)_2_ crystals.

Four nickel‐based MOFs were synthesised for comparative study. Ni‐catecholate MOFs, Ni_3_(HHTQ)_2_ and Ni_3_(HHTP)_2_, were obtained in a solvothermal reaction of the corresponding linker (H_6_HHTQ or H_6_HHTP) and Ni(OAc)_2_ in a DMF/water mixture. For the synthesis of MOFs based on amine‐containing linkers, Ni_3_(HITP)_2_ or Ni_3_(HATI)_2_, Ni(OAc)_2_ was reacted with H_6_HITP or H_6_HATI in DMSO/water mixture under solvothermal conditions in the presence of NaOAc. All products were characterised after drying by powder X‐ray diffraction (PXRD), nitrogen physisorption at 77 K, and scanning electron microscopy (SEM) (Figures 2, S5–S8, S10–S12 and S14).

The PXRD patterns of all compounds were subjected to Rietveld refinement. The crystal structures of Ni_3_(HITP)_2_ and Ni_3_(HATI)_2_ could be confirmed (Figures S7 and S8). In the case of Ni_3_(HHTP)_2_, the Rietveld refinement using the earlier reported structure resulted in poor convergence.^[^ ^43^ ^]^ Therefore, the defect‐free structure with AB‐stacking of layers was used, leading to improved convergence (Figure S6).

The structure of Ni_3_(HHTQ)_2_ converged with a low *R*_p_ of 2.73% and *R*_wp_ of 3.64%.^[^ ^44^ ^]^ The broad peak at 2*θ* = 27.0° originates from the disordered stacking in [001] direction, which is a typical feature of layered MOFs.^[^ ^45^ ^]^

To investigate the porosity of Ni_3_(HHTQ)_2_, the nitrogen physisorption isotherm at 77 K was measured after the desolvation in a dynamic vacuum at 373 K. The Brunauer–Emmett–Teller (BET) surface area was determined to be 480 m^2^ g^−1^ (Figure 2c). The surface area is comparable to that of Ni_3_(HHTP)_2_ and lower than the surface area of Ni_3_(HITP)_2_ and Ni_3_(HATI)_2_ (Table S5). The pore size of Ni_3_(HHTQ)_2_ derived using nonlocal density functional theory (NLDFT), is centred at 1.63 nm and is close to the value obtained from the MOF structure (ca. 2.0 nm, taking the van der Waals radii into account, Figure 2b). The thermogravimetric analysis (TGA) in air (Figure S9a) shows a weight loss below ca. 473 K, which can be attributed to the removal of solvent molecules such as water adsorbed during sample preparation. The second weight loss between 473 and 673 K corresponds to the thermal decomposition of the Ni_3_(HHTQ)_2_ framework. SEM image reveals typical rod‐like crystals of Ni_3_(HHTQ)_2_ (Figure 2d), and elemental mapping confirms the uniform distribution of Ni, C, O and N elements (Figure S13).

### Electrocatalytic Performance for GOR and HER

The electrocatalytic performance of four MOF catalysts towards GOR and OER in an alkaline medium (1.0 M KOH) was investigated in a typical three‐electrode setup. The electrochemical measurements (Figure S15a,b) indicated that Ni_3_(HHTQ)_2_ has the highest GOR electrocatalytic activity and fastest kinetics among MOFs investigated. The enhanced GOR performance of Ni_3_(HHTQ)_2_ could be partially attributed to the higher electrochemical surface‐active area (ECSA), which is related to the effective specific surface area in the electrochemical system. It can be estimated from double‐layer capacitance (*C*_dl_) acquired from CV curves measured at varying scan rates (Figure S16). The *C*_dl_ of Ni_3_(HHTQ)_2_, Ni_3_(HHTP)_2_, Ni_3_(HITP)_2,_ and Ni_3_(HATI)_2_ were 6.76, 4.81, 4.06, and 3.29 mF cm^−2^, respectively (according to the calculations from the data presented in Figure S17). To verify the feasibility of simultaneous generation of hydrogen and value‐added products, the samples were used as a cathode to evaluate HER activity in 1.0 M KOH solution. For HER, Ni_3_(HHTQ)_2_ also demonstrates better performance compared to that of Ni_3_(HHTP)_2_, Ni_3_(HITP)_2,_ and Ni_3_(HATI)_2_ (Figure S26a). It delivers a current density of 10 mA cm^−2^ with an overpotential of 524 mV. To further study the HER electrocatalytic activity, the ECSA values of different samples were compared by corresponding *C*_dl_ (Figures S27 and 28), and it is evident that the C_dl_ value of Ni_3_(HHTQ)_2_ is higher than those of Ni_3_(HHTP)_2_, Ni_3_(HITP)_2,_ and Ni_3_(HATI)_2_. Moreover, the Tafel slope (Figure S26b) of Ni_3_(HHTQ)_2_ is 135 mV dec^−1^, which is much lower than the Tafel slope of Ni_3_(HHTP)_2_ (140 mV dec^−1^), Ni_3_(HITP)_2_ (188 mV dec^−1^), and Ni_3_(HATI)_2_ (216 mV dec^−1^), suggesting the faster catalytic kinetics of HER. The PXRD and SEM results of the MOFs after GOR and HER (Figures S19 and S29) further confirm the stability of the compounds under reaction conditions.

**Figure 3:**
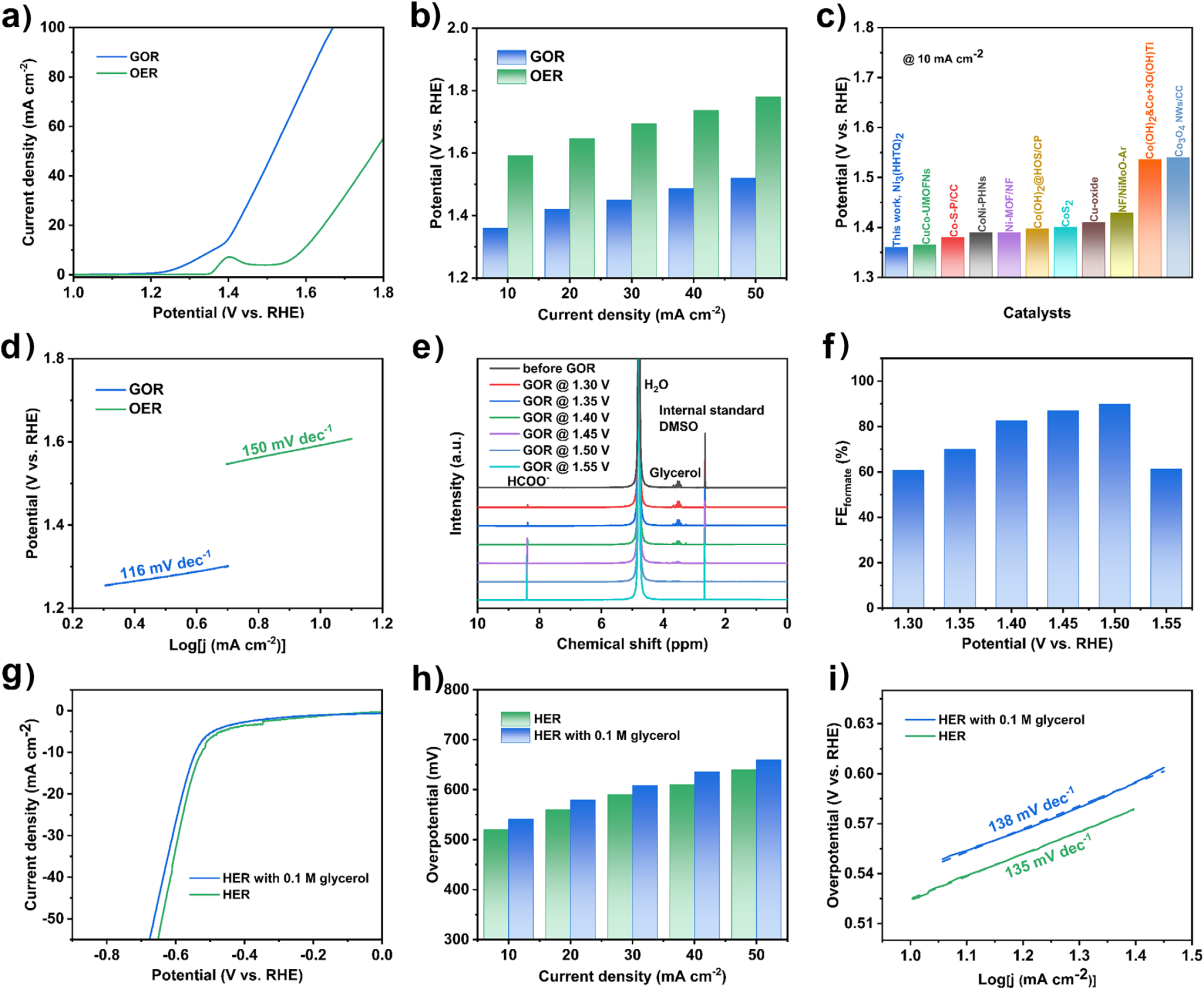
a) LSV curves of Ni_3_(HHTQ)_2_ anode in 1.0 M KOH with (blue) and without (green) glycerol (0.1 M). b) Potential comparisons to achieve different current densities for GOR and OER. c) Performance comparisons of the electrochemical biomass reforming systems in alkaline solutions. d) Corresponding Tafel plots of Ni_3_(HHTQ)_2_ for GOR and OER. e) ^1^H NMR spectra of the electrolytes before and after 8 h GOR on Ni_3_(HHTQ)_2_ electrode at various oxidation potentials. f) FEs of Ni_3_(HHTQ)_2_ for formate production at various oxidation potentials. g) LSV curves of Ni_3_(HHTQ)_2_ cathode for HER in 1.0 M KOH with and without 0.1 M glycerol addition. h) Overpotential comparisons to achieve different current densities for HER with and without 0.1 M glycerol addition. i) Corresponding Tafel plots of Ni_3_(HHTQ)_2_ for HER in 1.0 M KOH with and without 0.1 M glycerol addition.

After selecting the best catalyst, the GOR and HER performance of Ni_3_(HHTQ)_2_ was analysed in detail. Figure 3a displays the linear sweep voltammetry (LSV) curves of Ni_3_(HHTQ)_2_ for 1.0 M KOH before and after the glycerol addition, corresponding to GOR and OER reactions, respectively. In the absence of glycerol, the electrode exhibits moderate OER performance as it requires a relatively high potential of 1.59 V to reach the current density of 10 mA cm^−2^. The peak located at about 1.4 V can be attributed to the Ni^2+^/Ni^3+^ redox couple.^[^ ^46^ ^]^ After introducing 0.1 M glycerol, the current density increased significantly, and the GOR potential was reduced to 1.36 V at the current density of 10 mA cm^−2^. The impact of glycerol concentration on the Ni_3_(HHTQ)_2_ performance in GOR was also explored (Figure S18). It can be evidently observed that the best GOR performance was obtained in 1.0 M KOH solution containing 0.1 M glycerol.

The potential comparisons under the same current densities was further used to investigate the difference in electrocatalytic activity between GOR and OER. It can be seen in Figure 3b that the GOR potentials decrease by at least 227 mV to yield the current densities of 10, 20, 30, 40, and 50 mA cm^−2^, which are lower than the anode potentials of most recently reported biomass valorisation systems (Table S7 and Figure 3c). Furthermore, the Tafel slope (Figure 3d) of Ni_3_(HHTQ)_2_ towards GOR was determined to be 116 mV dec^−1^, which is lower than the 150 mV dec^−1^ of OER, indicating a more favourable kinetic for electro‐oxidation of glycerol.

The electrolytes after 8 h GOR were collected and analysed by ^1^H NMR spectroscopy to identify the electro‐oxidation products and to obtain the faradaic efficiencies (FEs). The ^1^H NMR spectra of the dissolved products obtained in electrolysis experiments (oxidation potential of 1.30 V was applied for 8 h) reveal that formate is the main product (Figure 3e). It is worth noting that competitive OER can be limited by adopting a relatively low potential of 1.30 V. In addition to the formate as a major product, glycerate and glycolate could be identified in the high‐resolution ^1^H NMR spectra (Figure S21), but their amounts are much lower. To determine the FEs of formate production, the calibration curve (Figure S22b) for formic acid was obtained based on ^1^H NMR spectra (Figure S22a) in a range of standard concentrations of formic acid. The prepared Ni_3_(HHTQ)_2_ electrode displays over 60% FE for formate formation in the range of oxidation potentials from 1.30 to 1.55 V, with the highest FE of 90% being achieved at the oxidation potential of 1.50 V. At lower oxidation potentials, the C─C bonds are difficult to break, whereas with a continuous increase in oxidation potential, the OER competes with GOR and reduces the FEs of formate.^[^ ^47^ ^]^ As shown in Figure S23a, the current density remained basically unchanged during continuous electrolysis for 8 h at 1.30 V, suggesting excellent long‐term stability of the Ni_3_(HHTQ)_2_ anode under GOR conditions.

The LSV curves before and after 8 h of electrolysis (Figure S24a) showed a slight decrease in electrocatalytic activity. This may be due to the consumption of glycerol and the pH change of electrolyte during long‐term electrolysis.^[^ ^48^ ^]^ The catalyst after GOR was characterised by PXRD, SEM, and elemental analysis (Figures S20 and S25). After the long‐term electrochemical measurement, the crystalline structure and morphology of Ni_3_(HHTQ)_2_ are almost preserved, further verifying the outstanding stability of the catalyst.

Since Ni_3_(HHTQ)_2_ showed the best results in the comparative HER study, the tolerance of HER against glycerol and the impact of glycerol on the performance in HER were studied for Ni_3_(HHTQ)_2_ (Figure 3g). The LSV curve only shows a slight deviation after the addition of 0.1 M glycerol, illustrating that it has almost no effect on the performance of Ni_3_(HHTQ)_2_. The Tafel slope with glycerol is found to be comparable to the value without glycerol (Figure 3i). The results of CA and LSV measurements after the long‐term HER test (Figures S23b and S24b) also indicate that Ni_3_(HHTQ)_2_ possesses outstanding electrochemical stability for HER in an alkaline solution. In addition, the PXRD, SEM, and elemental mapping analysis after long‐term HER measurement (Figures S30 and S31) further proved the sufficiently stable crystalline structure and morphology of Ni_3_(HHTQ)_2_.

### GOR Reaction Mechanism Based on In Situ ^13^C EC–NMR

**Figure 4:**
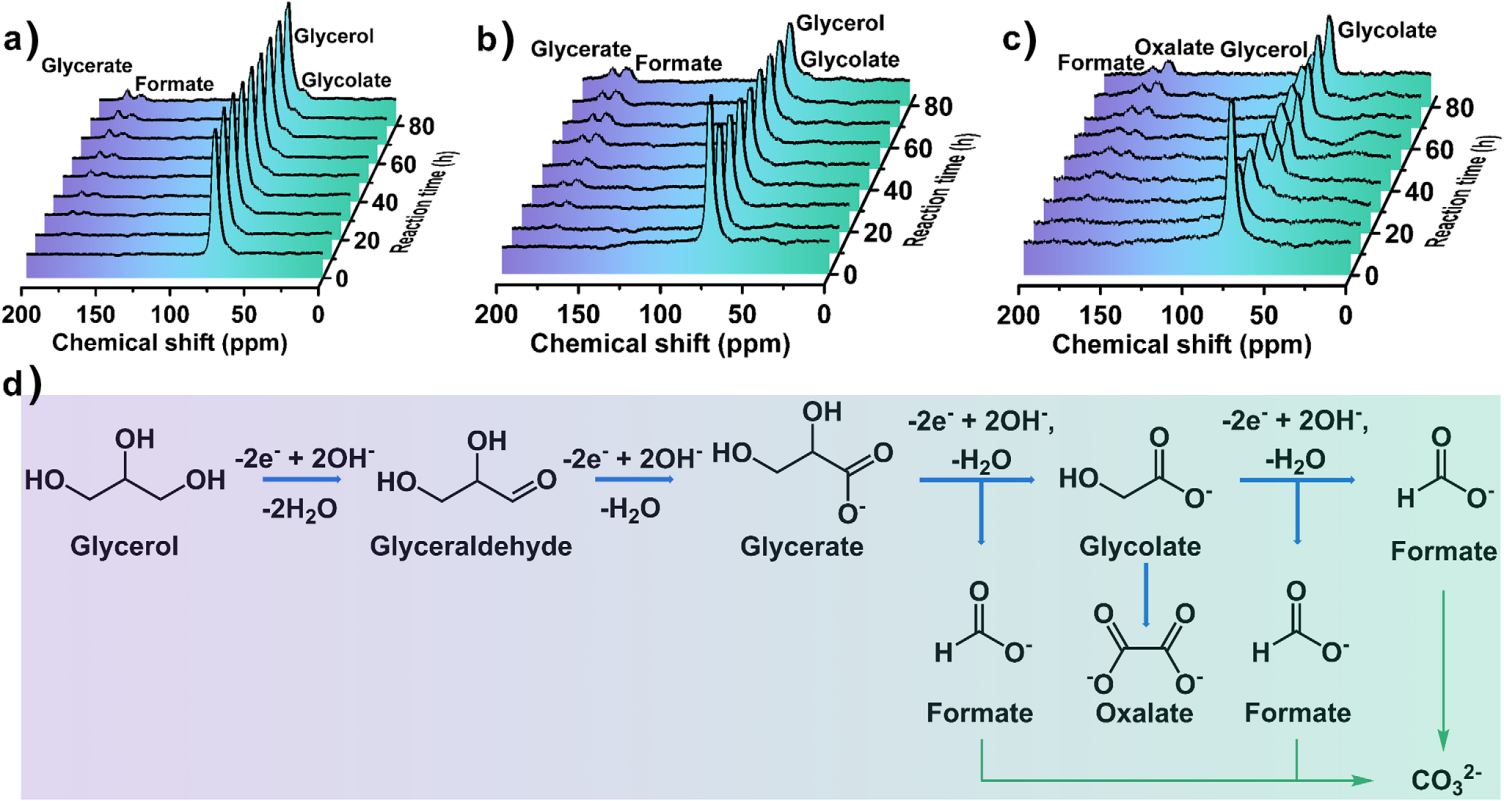
a)–c) In situ ^13^C EC–NMR spectra at oxidation potentials of 1.30, 1.50, and 1.55 V (vs. RHE). 1.0 M KOH with 0.1 M ^13^C labelled glycerol was used as the electrolyte. d) Proposed reaction mechanism of glycerol to formate in alkaline solution catalysed by Ni_3_(HHTQ)_2_. The blue and green arrows represent the dominant and minor reaction pathways.

The in situ ^13^C EC–NMR measurements with a commercial solid‐state NMR probe (eProbe Gmbh) were utilised to elucidate the GOR mechanism because it enables the noninvasive identification and quantification of reaction intermediates and product molecules in a time‐resolved manner with high chemical specificity.^[^ ^49^ ^]^ The customised 3D‐printed cell was utilised, enabling applied potential control (Figure S32). The ^13^C‐labeled glycerol (HOCH_2_)_2_^13^CHOH was used to enhance the sensitivity. The intensity of the NMR signals was monitored as a function of time during GOR. The recorded in situ ^13^C EC–NMR spectra at three different oxidation potentials of 1.30, 1.50, and 1.55 V over 80 h reaction time are presented in Figure 4a–c. Quantitative analysis of the compounds formed in GOR at particular oxidation potentials was carried out (Figures S36, S38, and S39) after subtraction of the polymer signals (Figure S34) located at 26 and 129–139 ppm and originating from 3D printed cells. Combining the results of in situ ^13^C EC–NMR experiments with corresponding compound concentration profiles, it can be seen that the signal at 73 ppm can be assigned to glycerol, and its peak intensity and concentration decrease with the measurement time, suggesting glycerol is being consumed as the starting material. At lower oxidation potentials of 1.30 and 1.50 V, the peaks detected in ^13^C NMR spectra can be attributed to glycolate (60 ppm), formate (172 ppm), and glycerate (180 ppm), whose intensities increase during the reaction time. However, at the higher oxidation potential of 1.55 V, the product distribution is different, except for glycolate and formate, the peak located at 160 ppm can be assigned to oxalate.

Thus, the product distribution is closely related to the oxidation potentials, and the amount of glycerol oxidised at a higher oxidation potential is higher than that at a lower oxidation potential. Sun et al. have reported a similar observation for GOR where the product distribution depends on the oxidation potentials.^[^ ^50^ ^]^ It should be noted that a small amount of carbonate was identified in the ^13^C NMR spectra (Figure S35), originating from the further electrochemical oxidation of formate.

Based on in situ ^13^C EC–NMR findings and previous literature reports,^[^ ^51^, ^52^, ^53^, ^54^ ^]^ a possible reaction pathway for the electrochemical oxidation of glycerol to formate in the alkaline environment is proposed, as depicted in Figure 4d. At first, a primary hydroxyl group (─OH) on the electron‐rich glycerol molecule is oxidised to glyceraldehyde through the double electron transfer. Then, the highly active glyceraldehyde can be easily converted to glycerate with a carboxyl group (COO─). After the cleavage of C─C bond, this glycerate intermediate undergoes two‐electron transfer oxidation to generate equal quantities of glycolate and formate. Finally, in addition to the formation of formate molecules, the electron‐rich glycolate can be further oxidised to oxalate. As a result, almost all intermediates formed upon GOR can be potentially oxidised to formate.

### In Situ EC–Raman Identifying the Active Centres

An open question in the literature is whether Ni‐frameworks at high oxidation potentials are intrinsic catalysts or precatalysts transforming into NiOOH species with high electrocatalytic activity. Such metamorphosis has been, in particular, reported under harsh OER conditions.^[^ ^55^ ^]^

**Figure 5:**
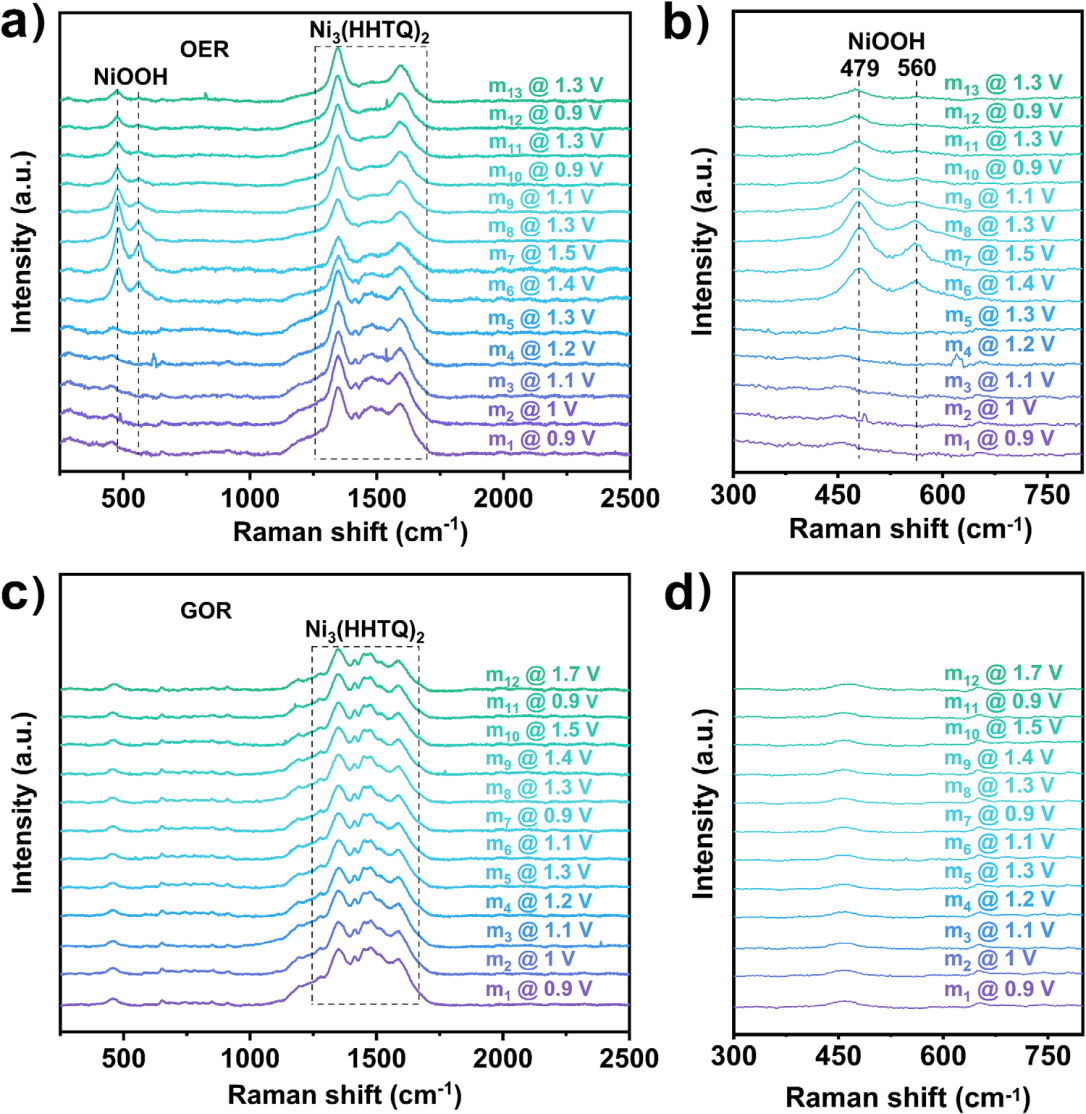
In situ EC–Raman spectra of Ni_3_(HHTQ)_2_ upon OER and GOR at different applied potentials. In the “m*_n_*” labelling, the “*n*” denotes the respective number of Raman measurements. a) and b) In situ EC–Raman spectra collected upon OER. 1.0 M KOH was used as the electrolyte. c) and d) In situ EC–Raman spectra collected upon GOR. 1.0 M KOH with 0.1 M glycerol was used as the electrolyte.

In situ EC–Raman spectroscopy is a powerful tool to detect surface reconstruction of the catalyst to determine the real active centres. Thus, in situ EC–Raman measurements were performed at various oxidation potentials during OER and GOR to obtain surface structure information of Ni_3_(HHTQ)_2_ by analysing the characteristic peaks of Ni_3_(HHTQ)_2_ between 1200 and 1600 cm^−1^(Figure 5a–d).

In situ EC–Raman spectra collected under OER conditions (Figure 5a) indicate two additional peaks of NiOOH appearing at potentials of 1.4 V or higher. These peaks are located at 479 and 560 cm^−1^, which are consistent with the spectroscopic features of NiOOH reported.^[^ ^56^, ^57^ ^]^ Secondly, with the appearance of NiOOH peaks, the band shape in the region of 1200 to 1600 cm^−1^ changes significantly, pointing to the degradation of Ni_3_(HHTQ)_2_. Moreover, the attenuation of the NiOOH peaks can be observed from scans 6 to 13 (spectra labelled as m_6_–m_13_ in Figure 5b). The m_8_ and m_13_ spectra were collected under the same applied potential, but the NiOOH peaks in m_13_ are less intense than those in m_8_. The intensity decrease is possibly related to irreversible reactions.

Significant differences were revealed under GOR conditions via in situ EC–Raman measurements. The oxidation potential was first increased to 1.3 V and then reduced to 0.9 V to avoid possible sample degradation above 1.4 V as observed in OER. At such conditions, there is no obvious alteration in these Raman spectra (Figure 5c, m_1_–m_7_). Therefore, the potential was further increased to 1.5 V, then lowered to 0.9 V, and finally even increased to 1.7 V. Interestingly, even under such harsh conditions, the Raman spectra remained unchanged, and no additional signals were detected. Hence, the proposed metamorphosis is not a general observation but critically depends on the presence and concentration of oxidisable substrates. In presence of glycerol, degradation of Ni_3_(HHTQ)_2_ and NiOOH formation is not detectable by means of Raman spectroscopy, whereas in pure KOH electrolyte, a clear transformation is observed.

To verify this finding, we replaced the glycerol solution with pure KOH solution, using a syringe while keeping all other conditions unchanged and applied the potentials of 1.3 and 1.5 V again. As expected, the NiOOH peaks appeared at 1.5 V, followed by a spectrum showing degradation at 0.9 V (Figure S40).

Therefore, the in situ EC–Raman results reveal the conversion of Ni_3_(HHTQ)_2_ to NiOOH proceeds only in pure KOH solution at potentials above 1.4 V. The presence of glycerol in KOH stabilises the Ni_3_(HHTQ)_2_ in the broad potential range from 1.4 to 1.7 V. These findings emphasise that stability considerations critically depend on the presence of oxidisable species affecting the structure of Ni‐O_4_ sites as active centres during the GOR process.

### Overall Electrochemical Performance for GOR Coupled with HER with Ni_3_(HHTQ)_2_ as Catalyst

**Figure 6:**
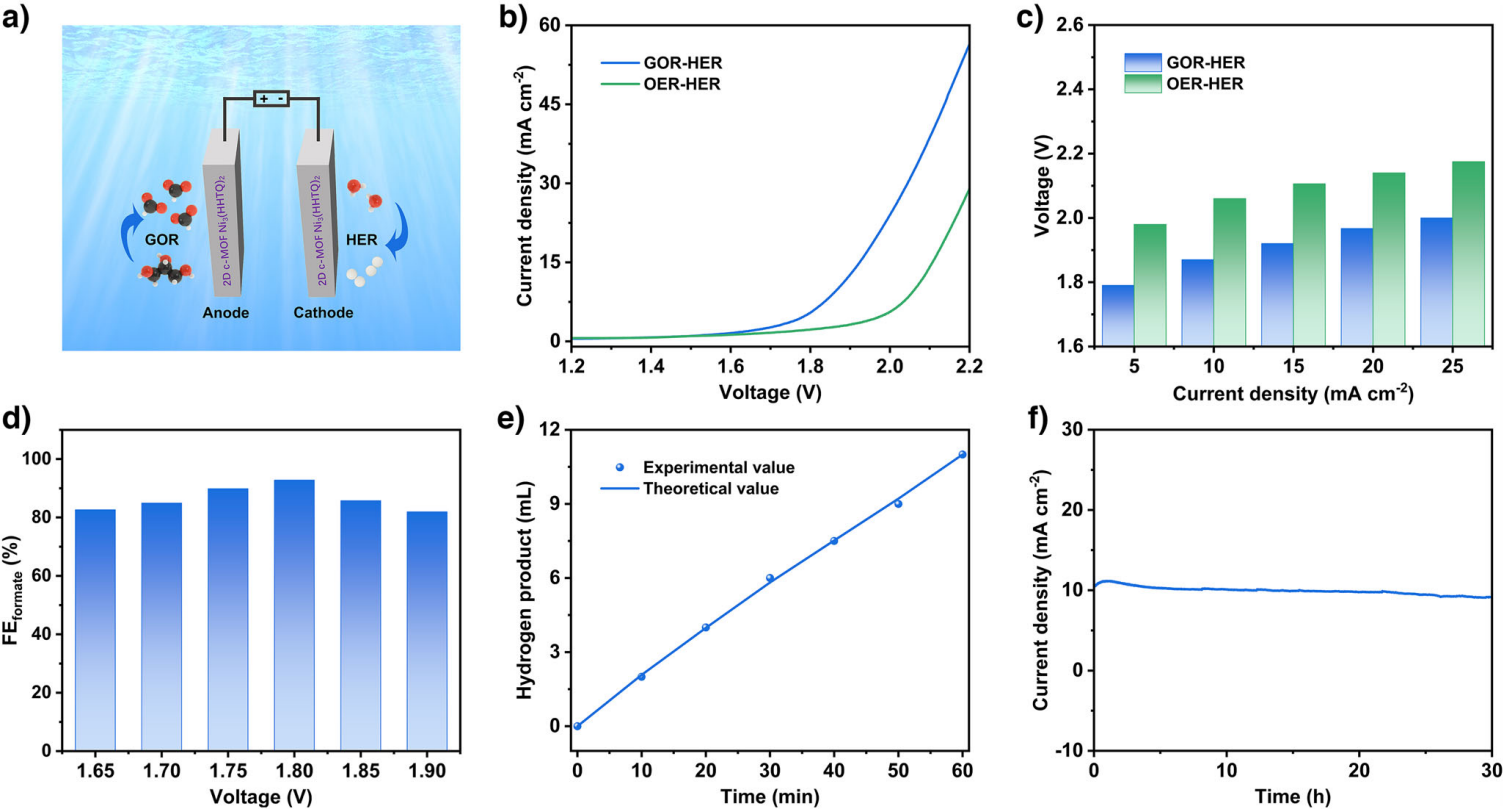
Overall electrochemical performance for GOR coupled with HER. a) Schematic illustration for a two‐electrode electrolytic cell for simultaneous formate and hydrogen production. b) LSV curves for GOR–HER and OER‐HER in 1.0 M KOH with and without 0.1 M glycerol addition. c) Comparisons of cell voltages for GOR–HER and OER‐HER at varying current densities. d) FEs of Ni_3_(HHTQ)_2_ couple for formate production at various applied voltages. e) Amount of hydrogen theoretically calculated and experimentally measured for the electrolytic cell of glycerol valorisation on Ni_3_(HHTQ)_2_ couple. f) Long‐term stability measurement of the electrolytic cell of GOR–HER based on Ni_3_(HHTQ)_2_ couple.

Given the outstanding electrochemical performance of Ni_3_(HHTQ)_2_ for both anodic GOR and cathodic HER, a two‐electrode electrolytic cell with a pair of Ni_3_(HHTQ)_2_ electrodes was implemented for the simultaneous production of hydrogen and formate and 1.0 M KOH solution containing 0.1 M glycerol was used as the electrolyte (Figure 6a). The input of 1.79, 1.87, 1.92, 1.97, and 2 V is required to afford current densities of 5, 10, 15, 20, and 25 mA cm^−2^, demonstrating the prominent performance of Ni_3_(HHTQ)_2_ towards GOR–HER (Figure 6b,c). In contrast, a conventional electrolytic cell for water splitting (OER‐HER) was also tested under the same conditions, but without glycerol addition, and much higher voltages of 1.98, 2.06, 2.11, 2.14, and 2.18 V were needed to reach the identical current densities. In particular, comparing the voltages of the two electrolytic cells at the current density of 10 mA cm^−2^, the voltage of glycerol valorisation is 194 mV lower than the value required for electrolytic water splitting, indicating the reduced energy consumption for glycerol electro‐oxidation coupled with hydrogen generation.

After electrolysis at a series of voltages applied for 8 h, the anode GOR products were also analysed by ^1^H NMR (Figure S41). The Ni_3_(HHTQ)_2_ catalyst has more than 80% of FEs for formate generation over a wide range of applied voltages from 1.65 to 1.90 V, with a maximum value of 93% at the applied voltage of 1.80 V (Figure 6d). Meanwhile, the FE for hydrogen production is close to 100% (Figure 6e). The long‐term stability for the electrolytic cell of GOR–HER was further estimated by CA measurement (Figure 6f) at the current density of 10 mA cm^−2^.

Despite some fluctuation, the current density is basically maintained at 10 mA cm^−2^ for 30 h, illustrating excellent long‐term stability for the production of both hydrogen and formate. The PXRD, SEM, and corresponding elemental mapping analysis after the long‐term electrolysis towards GOR–HER (Figures S43 and 44) further proved that the crystal structure and morphology of Ni_3_(HHTQ)_2_ did not change significantly.

### Theoretical Insight into GOR and HER

**Figure 7:**
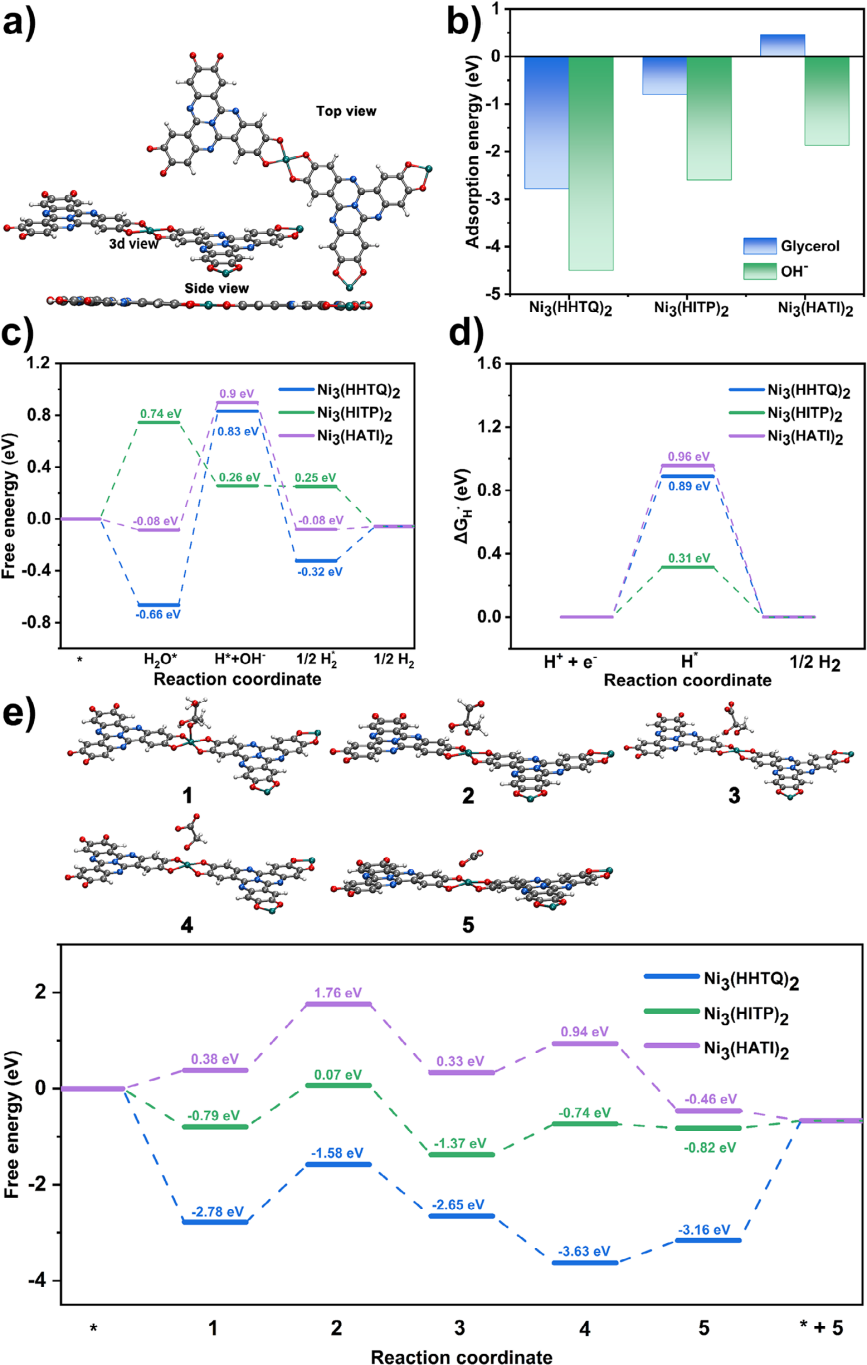
DFT calculations for electro‐reforming of glycerol and the asterisk (*) represents the active site of the catalyst that can adsorb and desorb reactants and intermediates during the specific reaction process. a) 3D, top, and side view of Ni_3_(HHTQ)_2_ model. b) Adsorption free energies of glycerol and OH^−^ on Ni‐X_4_ active sites in Ni_3_(HHTQ)_2_, Ni_3_(HITP)_2,_ and Ni_3_(HATI)_2_, respectively. c) Gibbs free energy diagram of alkaline HER on Ni‐X_4_ sites in Ni_3_(HHTQ)_2_, Ni_3_(HITP)_2,_ and Ni_3_(HATI)_2_, respectively. d) Gibbs free energy profiles of H atom adsorption on Ni_3_(HHTQ)_2_, Ni_3_(HITP)_2,_ and Ni_3_(HATI)_2_, respectively. e) Gibbs free energy diagrams for GOR on Ni_3_(HHTQ)_2_, Ni_3_(HITP)_2,_ and Ni_3_(HATI)_2_, respectively. 1, one glycerol molecule; 2, one glyceraldehyde molecule; 3, one glycerate molecule; 4, one glycolate molecule; 5, one formate molecule.

Density functional theory (DFT) calculations were performed to unveil the origin of the remarkable performance of Ni_3_(HHTQ)_2_ towards both HER and GOR, among other MOFs investigated. The crystal structures of Ni_3_(HHTQ)_2_, Ni_3_(HITP)_2,_ and Ni_3_(HATI)_2_^[^ ^24^, ^44^ ^]^ were optimized first (Figures 7a and S45–S47). The calculation box, including one infinite single layer terminated by vacuum, was constructed and optimised to study the interaction of the MOF with the molecule of interest. In the next step, water adsorption was studied for all three compounds.

The adsorption of water on Ni_3_(HHTQ)_2_ (Figures 7c and S48) is strong, reflected in the free energy of −0.66 eV (−63.68  kJ mol^−1^), which is not the case for the other two catalysts, i.e., +0.74 eV (71.40  kJ mol^−1^) for Ni_3_(HITP)_2_ and −0.08 eV (−7.72  kJ mol^−1^) for Ni_3_(HATI)_2_. The water oxygen atom is in close proximity to the catalytic Ni centre (2.34 Å) for the Ni_3_(HHTQ)_2_. For the Ni_3_(HITP)_2_ and Ni_3_(HATI)_2,_ distances are increased to 2.53 and 3.50 Å. The water on Ni_3_(HATI)_2_ does not bind to the Ni centre. In contrast to Ni_3_(HHTQ)_2_ and Ni_3_(HITP)_2_, the interaction in Ni_3_(HATI)_2_ is dominated by hydrogen bonds (Figures S49–S51) to the nitrogen atoms of ligands.

The water molecule in the alkaline solution binds to the Ni and dissociates, leaving an adsorbed hydrogen atom and a hydroxyl group (a process known as Volmer steps). The adsorbed H atom (H*) remains adsorbed on Ni_3_(HHTQ)_2_ (Figure S49). The NH groups in Ni_3_(HITP)_2_ and Ni_3_(HATI)_2_ (Figures S50 and S51), however, interact with the generated H* to form NH_2_ groups. Therefore, the position closer to the metallic centres is energetically unfavourable on Ni_3_(HATI)_2_/Ni_3_(HITP)_2_, compared to the positions at the NH groups of ligands, as determined by the geometry optimisations. We expect this difference to influence the performance of the catalysts in HER.

The Gibbs free energy for hydrogen adsorption (Δ*G*_H*_) is generally considered another crucial descriptor to evaluate the performance of catalysts towards HER. Furthermore, based on the Sabatier principle, excellent HER electrocatalysts should have moderate hydrogen adsorption energy.^[^ ^58^, ^59^ ^]^ The calculation results, shown in Figure 7d, indicate the value of Δ*G*_H*_ for Ni‐O_4_ site in Ni_3_(HATI)_2_ to be 0.96 eV (93.19 kJ mol^−1^) and the lower value of 0.31 eV (30.37 kJ mol^−1^) for Ni_3_(HITP)_2_. It is noteworthy that although Δ*G*_H*_ of Ni_3_(HITP)_2_ is closer to thermodynamic value compared to that of Ni_3_(HHTQ)_2_ (0.89 eV, 85.74 kJ mol^−1^), the water dissociation step for it is much slower than that for Ni_3_(HHTQ)_2_, resulting in a slower entire HER kinetic.

On the catalyst's surface, the glycerol molecule and hydroxyl ion coparticipate in the GOR process, hence, the effective balance of adsorption energies towards reactants is significant for high selectivity. Therefore, the adsorption energies for glycerol molecule (C_3_H_8_O_3_^*^) and hydroxyl (OH^*^) on the surface of three MOFs were investigated (Figure 7b). The adsorption energies of C_3_H_8_O_3_^*^ and OH^*^ on Ni_3_(HHTQ)_2_ have the negative values of −2.78 eV (−268.25 kJ mol^−1^) and −4.49 eV (−429.39 kJ mol^−1^), respectively.

The Gibbs free energy landscapes for the entire catalytic process from glycerol to formate were calculated to rationalise the proposed GOR mechanism based on in situ ^13^C EC–NMR and EC–Raman results. During the overall GOR process in Figure 7e, we find the adsorption energies of reaction intermediates for Ni_3_(HHTQ)_2_ are shifted to more negative values (thus favouring adsorption), whereas Ni_3_(HITP)_2_ and Ni_3_(HATI)_2_ have some adsorption energies whose values are close to or above 0. Therefore, the adsorption of reaction intermediates on Ni_3_(HITP)_2_/Ni_3_(HATI)_2_ is unfavourable, hindering the GOR process (Figures S52 and S53).

Secondly, Ni_3_(HHTQ)_2_ layer is entirely flat in the geometry‐optimised state but tends to form a pocket at the Ni site (Figure S45). The pocket should allow the different adsorbates to better approach the Ni‐O_4_ active centres with a lower energy barrier. However, for Ni_3_(HITP)_2_ and Ni_3_(HATI)_2_, the interaction between layers instead influences the stacking of layers, which leads to the observed serrated (AA'AA’) stacking (Figures S46 and S47), which further shields the active centres from the GOR process as well as influences the electronic properties.^[^ ^60^ ^]^ These results unveil that Ni_3_(HHTQ)_2_ possesses optimal Ni‐O_4_ active sites among the tested materials, resulting in an outstanding bifunctional electrochemical performance for HER and GOR.

## Conclusion

In summary, we identified an outstanding catalyst among a series of Ni‐based 2D c‐MOFs with Ni‐X_4_ (X = O and N) active sites for GOR electrocatalysis. In particular, Ni_3_(HHTQ)_2_ shows prominent GOR activity and selectivity, delivering a current density of 10 mA cm^−2^ at only 1.36 V while possessing FE as high as 90% for converting glycerol to formate at 1.50 V. Moreover, by utilising Ni_3_(HHTQ)_2_ as cathode and anode in an electrolytic cell, GOR is effectively coupled with HER, reducing energy consumption. Because of the more favourable GOR thermodynamics, the two‐electrode system only needs a cell voltage of 1.87 V at a current density of 10 mA cm^−2^, which is 194 mV lower than conventional water splitting with the same Ni_3_(HHTQ)_2_ electrodes. In situ^13^C EC–NMR and EC–Raman spectroscopies revealed the reaction mechanism of Ni_3_(HHTQ)_2_ towards GOR and identified the Ni‐O_4_ sites as the active centres. Theoretical calculations pointed out the reason for the excellent performance of Ni_3_(HHTQ)_2_, that is, favourable adsorption of key intermediates and weak interlayer interactions, enhancing the accessibility of the active sites for the substrate. Our work not only sheds light on the rational design of 2D c‐MOFs as highly active catalysts but also provides an energy‐saving and cost‐effective strategy to develop sustainable production for high‐valued chemicals from biomass conversion products.

## Author Contributions

Y.L.: Experiments, data processing, and manuscript writing; M.B.: DFT calculations for GOR and HER; S.R.: In situ EC–Raman spectroscopy and corresponding Raman data analysis; S.A.S.: In situ ^13^C EC–NMR spectroscopy; V.B.: PXRD refinement; Y.L.: Ni_3_(HATI)_2_ MOF and HITP ligand synthesis; L.S.: Support by the in situ electrolytic measurements; A.D.: SEM measurement; X.F.: Ni_3_(HATI)_2_ MOF and HITP ligand synthesis; I.M.W.: In situ EC–Raman spectroscopy and corresponding Raman data analysis; T.D.K.: DFT calculations for GOR and HER; A.H.K.: Design of the in situ ^13^C EC–NMR experiments and corresponding NMR data analysis; I.S. and S.K.: Project conceptualisation and supervision; S.K.: Funding acquisition and all authors: Review and editing.

## Conflict of Interests

The authors declare no conflict of interest.

## Supporting information



Supporting Information

Supporting Information

## Data Availability

The data that support the findings of this study are available from the corresponding author upon reasonable request.

## References

[1] J. Wu , X. Liu , Y. Hao , S. Wang , R. Wang , W. Du , S. Cha , X. Y. Ma , X. Yang , M. Gong , Angew. Chem. Int. Ed. 2023, 62, 202216083. 10.1002/anie.202216083 36594790

[2] Y. Wang , Y.‐Q. Zhu , Z. Xie , S.‐M. Xu , M. Xu , Z. Li , L. Ma , R. Ge , H. Zhou , Z. Li , X. Kong , L. Zheng , J. Zhou , H. Duan , ACS Catal. 2022, 12, 12432–12443.

[3] P. Prabhu , Y. Wan , J.‐M. Lee , Matter 2020, 3, 1162–1177.

[4] G. Chen , X. Li , X. Feng , Angew. Chem. Int. Ed. 2022, 61, 202209014. 10.1002/anie.202209014 PMC9826310 35849025

[5] L. Fan , B. Liu , X. Liu , N. Senthilkumar , G. Wang , Z. Wen , Energy Technol. 2021, 9, 2000804.

[6] J. Wu , X. Yang , M. Gong , Chinese J. Catal. 2022, 43, 2966–2986.

[7] B. Liu , G. Wang , X. Feng , L. Dai , Z. Wen , S. Ci , Nanoscale 2022, 14, 12841–12848. 36039893 10.1039/d2nr02689a

[8] L. Fan , Y. Ji , G. Wang , Z. Zhang , L. Yi , K. Chen , X. Liu , Z. Wen , J. Energy Chem. 2022, 72, 424–431.

[9] L. S. Oh , M. Park , Y. S. Park , Y. Kim , W. Yoon , J. Hwang , E. Lim , J. H. Park , S. M. Choi , M. H. Seo , W. B. Kim , H. J. Kim , Adv. Mater. 2023, 35, 2203285. 10.1002/adma.202203285 35679126

[10] Z. He , J. Hwang , Z. Gong , M. Zhou , N. Zhang , X. Kang , J. W. Han , Y. Chen , Nat. Commun. 2022, 13, 3777. 35773257 10.1038/s41467-022-31484-0 PMC9246976

[11] J. Wu , J. Li , Y. Li , X. Y. Ma , W. Y. Zhang , Y. Hao , W. B. Cai , Z. P. Liu , M. Gong , Angew. Chem. Int. Ed. 2022, 61, 202113362. 10.1002/anie.202113362 34957665

[12] Y. Zhu , Q. Qian , Y. Chen , X. He , X. Shi , W. Wang , Z. Li , Y. Feng , G. Zhang , F. Cheng , Adv. Funct. Mater. 2023, 33, 2300547.

[13] X. Yu , R. B. Araujo , Z. Qiu , E. Campos dos Santos , A. Anil , A. Cornell , L. G. M. Pettersson , M. Johnsson , Adv. Energy Mater. 2022, 12, 2103750.

[14] Z. Xia , C. Ma , Y. Fan , Y. Lu , Y.‐C. Huang , Y. Pan , Y. Wu , Q. Luo , Y. He , C.‐L. Dong , S. Wang , Y. Zou , ACS Catal. 2024, 14, 1930–1938.

[15] Y. X. Duan , F. L. Meng , K. H. Liu , S. S. Yi , S. J. Li , J. M. Yan , Q. Jiang , Adv. Mater. 2018, 30, 1706194. 10.1002/adma.201706194 29473227

[16] J. Dong , Y. Liu , J. Pei , H. Li , S. Ji , L. Shi , Y. Zhang , C. Li , C. Tang , J. Liao , S. Xu , H. Zhang , Q. Li , S. Zhao , Nat. Commun. 2023, 14, 6849. 37891185 10.1038/s41467-023-42539-1 PMC10611760

[17] Y. Li , X. Wei , L. Chen , J. Shi , M. He , Nat. Commun. 2019, 10, 5335. 31767871 10.1038/s41467-019-13375-z PMC6877572

[18] Q. Jiang , C. Zhou , H. Meng , Y. Han , X. Shi , C. Zhan , R. Zhang , J. Mater. Chem. A 2020, 8, 15271–15301.

[19] U. Khan , A. Nairan , J. Gao , Q. Zhang , Small Struct. 2023, 4, 2200109.

[20] L. S. Xie , G. Skorupskii , M. Dinca , Chem. Rev. 2020, 120, 8536–8580. 32275412 10.1021/acs.chemrev.9b00766 PMC7453401

[21] H. Wu , J. Wang , W. Jin , Z. Wu , Nanoscale 2020, 12, 18497–18522. 32839807 10.1039/d0nr04458j

[22] J. Liu , X. Song , T. Zhang , S. Liu , H. Wen , L. Chen , Angew. Chem. Int. Ed. 2021, 60, 5612–5624. 10.1002/anie.202006102 32452126

[23] H. Zhong , M. Wang , G. Chen , R. Dong , X. Feng , ACS Nano 2022, 16, 1759–1780. 35049290 10.1021/acsnano.1c10544

[24] Y. Lu , Z. Hu , P. Petkov , S. Fu , H. Qi , C. Huang , Y. Liu , X. Huang , M. Wang , P. Zhang , U. Kaiser , M. Bonn , H. I. Wang , P. Samori , E. Coronado , R. Dong , X. Feng , J. Am. Chem. Soc. 2024, 146, 2574–2582. 38231138 10.1021/jacs.3c11172

[25] Y. Lu , Y. Zhang , C. Y. Yang , S. Revuelta , H. Qi , C. Huang , W. Jin , Z. Li , V. Vega‐Mayoral , Y. Liu , X. Huang , D. Pohl , M. Polozij , S. Zhou , E. Canovas , T. Heine , S. Fabiano , X. Feng , R. Dong , Nat. Commun. 2022, 13, 7240. 36433971 10.1038/s41467-022-34820-6 PMC9700716

[26] M. Ko , L. Mendecki , K. A. Mirica , Chem. Commun. 2018, 54, 7873–7891. 10.1039/c8cc02871k 29926846

[27] L. Lin , Q. Zhang , Y. Ni , L. Shang , X. Zhang , Z. Yan , Q. Zhao , J. Chen , Chem 2022, 8, 1822–1854.

[28] Y. He , F. Yan , X. Zhang , C. Zhu , Y. Zhao , B. Geng , S. Chou , Y. Xie , Y. Chen , Adv. Energy Mater. 2023, 13, 2204177.

[29] H. Huang , Y. Zhao , Y. Bai , F. Li , Y. Zhang , Y. Chen , Adv. Sci. 2020, 7, 2000012. 10.1002/advs.202000012 PMC7201256 32382489

[30] H. Jia , Y. Yao , J. Zhao , Y. Gao , Z. Luo , P. Du , J. Mater. Chem. A 2018, 6, 1188–1195.

[31] R. Iqbal , S. Ali , A. Saleem , M. K. Majeed , A. Hussain , S. Rauf , A. Rehman Akbar , H. Xu , L. Qiao , W. Zhao , Chem. Eng. J. 2023, 455, 140799.

[32] E. M. Miner , S. Gul , N. D. Ricke , E. Pastor , J. Yano , V. K. Yachandra , T. Van Voorhis , M. Dincă , ACS Catal. 2017, 7, 7726–7731.

[33] Y. Lian , W. Yang , C. Zhang , H. Sun , Z. Deng , W. Xu , L. Song , Z. Ouyang , Z. Wang , J. Guo , Y. Peng , Angew. Chem. Int. Ed. 2020, 59, 286–294. 10.1002/anie.201910879 31638312

[34] R. D. Ross , H. Sheng , Y. Ding , A. N. Janes , D. Feng , J. R. Schmidt , C. U. Segre , S. Jin , J. Am. Chem. Soc. 2022, 144, 15845–15854. 35985015 10.1021/jacs.2c06810

[35] L. Sun , X. Jin , T. Su , A. C. Fisher , X. Wang , Adv. Mater. 2023, 36, 2306336. 10.1002/adma.202306336 37560974

[36] K. Dong , J. Liang , Y. Wang , L. Zhang , Z. Xu , S. Sun , Y. Luo , T. Li , Q. Liu , N. Li , B. Tang , A. A. Alshehri , Q. Li , D. Ma , X. Sun , ACS Catal. 2022, 12, 6092–6099.

[37] Y. Liu , S. Li , L. Dai , J. Li , J. Lv , Z. Zhu , A. Yin , P. Li , B. Wang , Angew. Chem. Int. Ed. 2021, 60, 16409–16415. 10.1002/anie.202105966 33961317

[38] J. D. Yi , D. H. Si , R. Xie , Q. Yin , M. D. Zhang , Q. Wu , G. L. Chai , Y. B. Huang , R. Cao , Angew. Chem. Int. Ed. 2021, 60, 17108–17114. 10.1002/anie.202104564 34033203

[39] H. Zhong , M. Ghorbani‐Asl , K. H. Ly , J. Zhang , J. Ge , M. Wang , Z. Liao , D. Makarov , E. Zschech , E. Brunner , I. M. Weidinger , J. Zhang , A. V. Krasheninnikov , S. Kaskel , R. Dong , X. Feng , Nat. Commun. 2020, 11, 1409. 32179738 10.1038/s41467-020-15141-y PMC7075876

[40] H. Zhong , M. Wang , M. Ghorbani‐Asl , J. Zhang , K. H. Ly , Z. Liao , G. Chen , Y. Wei , B. P. Biswal , E. Zschech , I. M. Weidinger , A. V. Krasheninnikov , R. Dong , X. Feng , J. Am. Chem. Soc. 2021, 143, 19992–20000. 34784212 10.1021/jacs.1c11158

[41] W. Xiong , X. Cheng , T. Wang , Y. Luo , J. Feng , S. Lu , A. M. Asiri , W. Li , Z. Jiang , X. Sun , Nano Res. 2020, 13, 1008–1012.

[42] Y. Xu , Q. Zhou , T. Liu , T. Ren , H. Yu , K. Deng , Z. Wang , L. Wang , H. Wang , Chem. Commun. 2023, 59, 7623–7626. 10.1039/d3cc01741a 37254963

[43] M. Hmadeh , Z. Lu , Z. Liu , F. Gándara , H. Furukawa , S. Wan , V. Augustyn , R. Chang , L. Liao , F. Zhou , E. Perre , V. Ozolins , K. Suenaga , X. Duan , B. Dunn , Y. Yamamto , O. Terasaki , O. M. Yaghi , Chem. Mater. 2012, 24, 3511–3513.

[44] J. H. Dou , M. Q. Arguilla , Y. Luo , J. Li , W. Zhang , L. Sun , J. L. Mancuso , L. Yang , T. Chen , L. R. Parent , G. Skorupskii , N. J. Libretto , C. Sun , M. C. Yang , P. V. Dip , E. J. Brignole , J. T. Miller , J. Kong , C. H. Hendon , J. Sun , M. Dinca , Nat. Mater. 2021, 20, 222–228. 33230325 10.1038/s41563-020-00847-7

[45] M. G. Campbell , D. Sheberla , S. F. Liu , T. M. Swager , M. Dinca , Angew. Chem. Int. Ed. 2015, 54, 4349–4352. 10.1002/anie.201411854 25678397

[46] Y. Li , X. Wei , L. Chen , J. Shi , M. He , Nat. Commun. 2019, 10, 5335. 31767871 10.1038/s41467-019-13375-z PMC6877572

[47] P. Wang , G. Wang , K. Chen , W. Pan , L. Yi , J. Wang , Q. Chen , J. Chen , Z. Wen , Nano Energy 2023, 118, 108992.

[48] Z. Ke , N. Williams , X. Yan , S. Younan , D. He , X. Song , X. Pan , X. Xiao , J. Gu , J. Mater. Chem. A 2021, 9, 19975–19983.

[49] J. B. Richter , C. Essbach , I. Senkovska , S. Kaskel , E. Brunner , Chem. Commun. 2019, 55, 6042–6045. 10.1039/c9cc02660f 31065638

[50] L. Huang , J.‐Y. Sun , S.‐H. Cao , M. Zhan , Z.‐R. Ni , H.‐J. Sun , Z. Chen , Z.‐Y. Zhou , E. G. Sorte , Y. J. Tong , S.‐G. Sun , ACS Catal. 2016, 6, 7686–7695.

[51] H. Wang , L. Thia , N. Li , X. Ge , Z. Liu , X. Wang , ACS Catal. 2015, 5, 3174–3180.

[52] L. Fan , Y. Ji , G. Wang , J. Chen , K. Chen , X. Liu , Z. Wen , J. Am. Chem. Soc. 2022, 144, 7224–7235. 35404594 10.1021/jacs.1c13740

[53] H. Yu , M. Hu , C. Chen , C. Hu , Q. Li , F. Hu , S. Peng , J. Ma , Angew. Chem. Int. Ed. 2023, 62, 202314569. 10.1002/anie.202314569 37942995

[54] H. Yu , W. Wang , Q. Mao , K. Deng , Z. Wang , Y. Xu , X. Li , H. Wang , L. Wang , Appl. Catal. B Environ. 2023, 330, 122617.

[55] S. Hou , W. Li , S. Watzele , R. M. Kluge , S. Xue , S. Yin , X. Jiang , M. Doblinger , A. Welle , B. Garlyyev , M. Koch , P. Muller‐Buschbaum , C. Woll , A. S. Bandarenka , R. A. Fischer , Adv. Mater. 2021, 33, 2103218. 34337809 10.1002/adma.202103218 PMC11468612

[56] J. Gallenberger , H. Moreno Fernández , A. Alkemper , M. Li , C. Tian , B. Kaiser , J. P. Hofmann , Catal. Sci. Technol. 2023, 13, 4693–4700.

[57] A. C. Garcia , T. Touzalin , C. Nieuwland , N. Perini , M. T. M. Koper , Angew. Chem. Int. Ed. 2019, 58, 12999–13003. 10.1002/anie.201905501 31250499

[58] Z. W. Chen , J. Li , P. Ou , J. E. Huang , Z. Wen , L. Chen , X. Yao , G. Cai , C. C. Yang , C. V. Singh , Q. Jiang , Nat. Commun. 2024, 15, 359. 38191599 10.1038/s41467-023-44261-4 PMC10774414

[59] D. Y. Kuo , H. Paik , J. Kloppenburg , B. Faeth , K. M. Shen , D. G. Schlom , G. Hautier , J. Suntivich , J. Am. Chem. Soc. 2018, 140, 17597–17605. 30463402 10.1021/jacs.8b09657

[60] A. Kuc , M. A. Springer , K. Batra , R. Juarez‐Mosqueda , C. Wöll , T. Heine , Adv. Funct. Mater. 2020, 30, 1908004.

