# Supplementary material for: 2D Conjugated Metal–Organic Frameworks as Electrocatalysts for Boosting Glycerol Upgrading Coupled with Hydrogen Production
Source: Angew Chem Int Ed Engl. 2025 May 8;64(27):e202502425. doi: 10.1002/anie.202502425 (PMC12207376; doi:10.1002/anie.202502425)
Supplement: Supplementary file 1 — Supporting Information [file ANIE-64-e202502425-s002.pdf]

Two-dimensional Conjugated Metal-Organic Frameworks as Electrocatalysts for Boosting Glycerol Upgrading Coupled with Hydrogen Production

Yutong Luo,[a] Michael Beerbaum,[b] Stefan Röher,[c] Sara Amanzadeh Salout,[d] Volodymyr Bon,[a] Yang Lu,[e] Leonid Shupletsov,[a] Ankita De,[a] Xinliang Feng,[e] Inez M. Weidinger,[c] Thomas D. Kühne,[b] Arafat Hossain Khan,[d] Irena Senkovska,[a] and Stefan Kaskel*[a]*

[a]Chair of Inorganic Chemistry I, Technische Universität Dresden, Bergstraße 66, Dresden 01069, Germany.
[b]Center for Advanced Systems Understanding and Helmholtz-Zentrum Dresden-Rossendorf, Technische Universität Dresden, Untermarkt 20, D-02826, Görlitz, Germany.

[c]Chair of Electrochemistry, Technische Universität Dresden, Zellescher Weg 19, 01069 Dresden, Germany.

[d]Chair of Bioanalytical Chemistry, Technische Universität Dresden, Bergstraße 66, Dresden 01069, Germany.

[e]Center for Advancing Electronics Dresden, Technische Universität Dresden, 01067 Dresden, Germany; Max-Planck-Institute of Microstructure Physics, Weinberg 2, 06120 Halle, Germany.

1. Reagents and materials

6-Nitroveratraldehyde (96%) was purchased from Thermo Fisher Scientific. Pyridine hydrochloride (98%) was purchased from Alfa Aesar. Diethyl ether (100%) and formic acid (99-100%) were purchased from VWR. Nafion solution (5 wt% in lower aliphatic alcohols and water), tin powder (99%), $Ni(OAc)_2{\cdot}4H_2O$ (98.0%), glycerol (99.5%), and sulfolane (99%) were purchased from Sigma-Aldrich. Acetic acid (100%) was purchased form Carl Roth GmbH. *N, N*-dimethylformamide (DMF, 99.5%), dimethyl sulfoxide (DMSO, 99.9%), and ethanol (99.8%) were obtained from Fisher Chemical. Ammonium acetate (NH_4OAc, 97%) was purchased from ABCR GmbH. Sodium acetate (NaOAc, 99%) and anhydrous magnesium sulfate ($MgSO_4$, 99%) were purchased from Grüssing GmbH. $CDCl_3$ (99.8%), DMSO-d_6 (99.8%), and deuterium oxide (D_2O, 99.95%) were purchased from Deutero GmbH. 2,3,6,7,10,11-Hexahydroxytriphenylene (HHTP, 95.0%) was purchased from TCI. Carbon paper (AvCarb P50, SKU: 590142-1, Lot Number: 4472-P50) purchased from the Fuel Cell Store was cut into pieces with 10 mm width × 20 mm length and sonicated in acetone, deionized water, and ethanol for 20 min each to remove the surface impurities and then kept at 298 K for further use.

2. Materials characterization

The powder X-ray diffraction (PXRD) patterns were obtained on Stoe Stadi P diffractometer using a Cu-Kα_1 radiation source (λ = 0.154059 nm) in a 2θ range from 2° to 50° at 298 K. Scanning electron microscopy (SEM) images were recorded on HITACHI SU8020 operated at an acceleration voltage of 2 kV. Nitrogen physisorption isotherms were performed on Autosorb IQ at 77 K. Before the measurements, 20 - 40 mg of the sample was activated in a vacuum at 373 K overnight. The Brunauer-Emmett-Teller (BET) area was derived from the N_2 isotherm at 77 K in the relative pressure range from 0.04 to 0.12. In this range, the R^2 of linear fitting was higher than 0.999. Pore size distribution was calculated from the desorption branch of the N_2 isotherms at 77 K by utilizing the Barrett-Joyner-Halenda (BJH) method. The thermogravimetric analysis (TGA) data were collected on STA 409 PC (NETZSCH) in air and STA 449F5 (NETZSCH) in Ar from 313

to 1273 K with a heating rate of 5 K min^{-1}.

3. Nuclear Magnetic Resonance (NMR)

The NMR spectra were collected on a Bruker Avance 300 MHz spectrometer. The chemical shifts are reported in parts per million (ppm). $CDCl_3$ (7.26 ppm) and DMSO-d_6 (2.50 ppm) were used as references for ^1H and ^{13}C NMR chemical shifts. For the calculation of Faradaic efficiency (FE), D_2O (4.79 ppm) is used for the references of ^1H chemical shift.

4. In situ Electrochemical (EC) - Raman spectroscopy

The ink for in *situ* EC-Raman measurement was prepared utilizing 1 mg of $Ni_3(HHTQ)_2$ added into the mixture of ethanol (147 μL), water (49 μL), and Nafion (4 μL of 5 wt% D-520 dispersion in water and 1-propanol). Then, 4 μL ink was drop cast onto the glassy carbon electrode, leading to a loading of about 0.1mg cm^{-2}. EC measurements were performed in a three-electrode rotating disc system (reference electrode Hg/HgO and counter electrode Pt wire) at a slow rotation (about 8 rpm) to ensure averaged spectra over multiple sample points. *In situ* EC-Raman measurement was conducted using a 561 nm Laser (Cobolt jive) with a power of about 3 mW using a confocal Raman microscope (S&I Monovista CRS+).

5. *Synthesis of 2,3,7,8,12,13-hexahydroxytricycloquinazoline (H_6HHTQ)*

The H_6HHTQ was synthesized following the reported procedure.[3]

5.1 Synthesis of 5,6-dimethoxyanthranil

Tin powder (13 g, 0.11 mol) was added in portions to the stirred mixture of 6-nitroveratraldehyde (5 g, 0.019 mol) and acetic acid (300 mL). The mixture was stirred at 298 K for 20 hours, then quenched with water and extracted with diethyl ether. The organic layer was collected and dried with anhydrous magnesium sulfate. Then, the solvent was removed using a rotary evaporator. The residue was purified by silica gel column chromatography using a mixture of iso-hexane and ethyl acetate (v/v = 1/1) as the eluent. Yield: 3.4 g (81%), white solid.

^{1}H NMR ($CDCl_3$): 8.81 (s, I H), 6.78 (s, I H), 6.64 (s, I H), 3.96 (s, 3 H), 3.90 (s, 3 H) ppm.

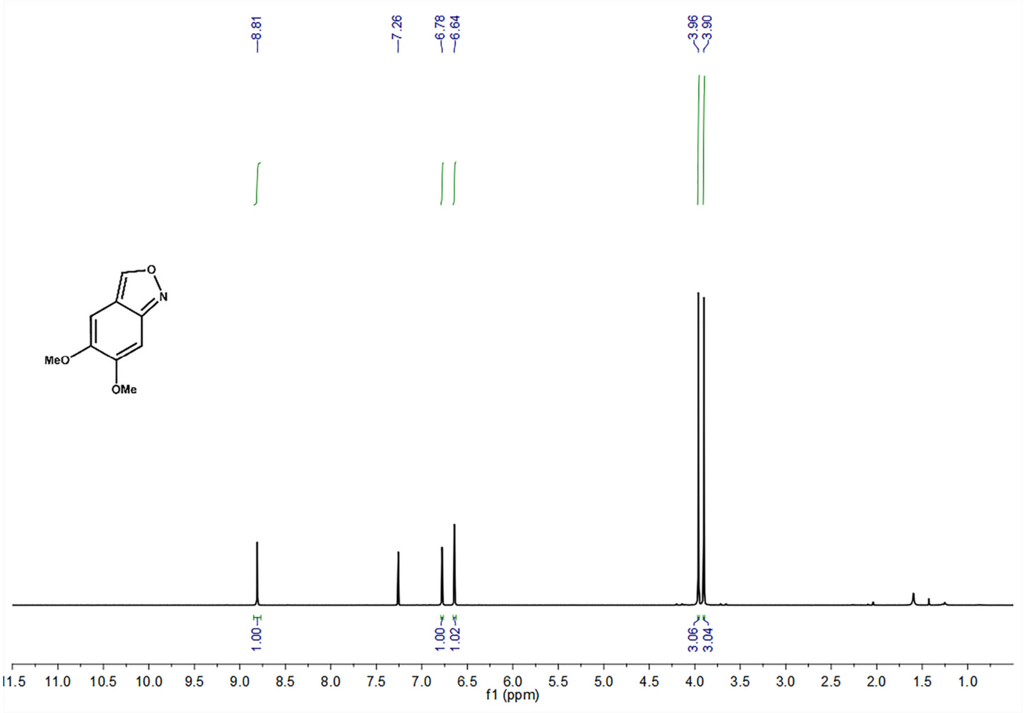

Figure S1. ^{1}H NMR spectrum ($CDCl_3$) of 5,6-dimethoxyanthranil.

5.2 Synthesis of compound 2,3,7,8,12,13-hexamethoxytricycloquinazoline

5,6-Dimethoxyanthranil (8.1 g, 0.045 mol) and ammonium acetate (24.3 g, 0.31 mol) were added into the flask containing sulfolane (202 mL) and acetic acid (17.5 M, 50.5 mL). The mixture was refluxed for 96 h in N_2 atmosphere and then cooled to 298 K. Water (600 - 800 mL) was added to the mixture, and the mixture was placed into the refrigerator at 274 K for 12 h. The resulting solid was collected by filtration. The solid was washed with water and methanol several times and dried under vacuum overnight (16 h).

Yield: 1.32 g (17.6%), a yellow-greenish solid.

^{1}H NMR ($CDCl_3$): 7.66 (s, 3H), 6.87 (s, 3H), 4.02 (s, 9H), 4.00 (s, 9H) ppm.

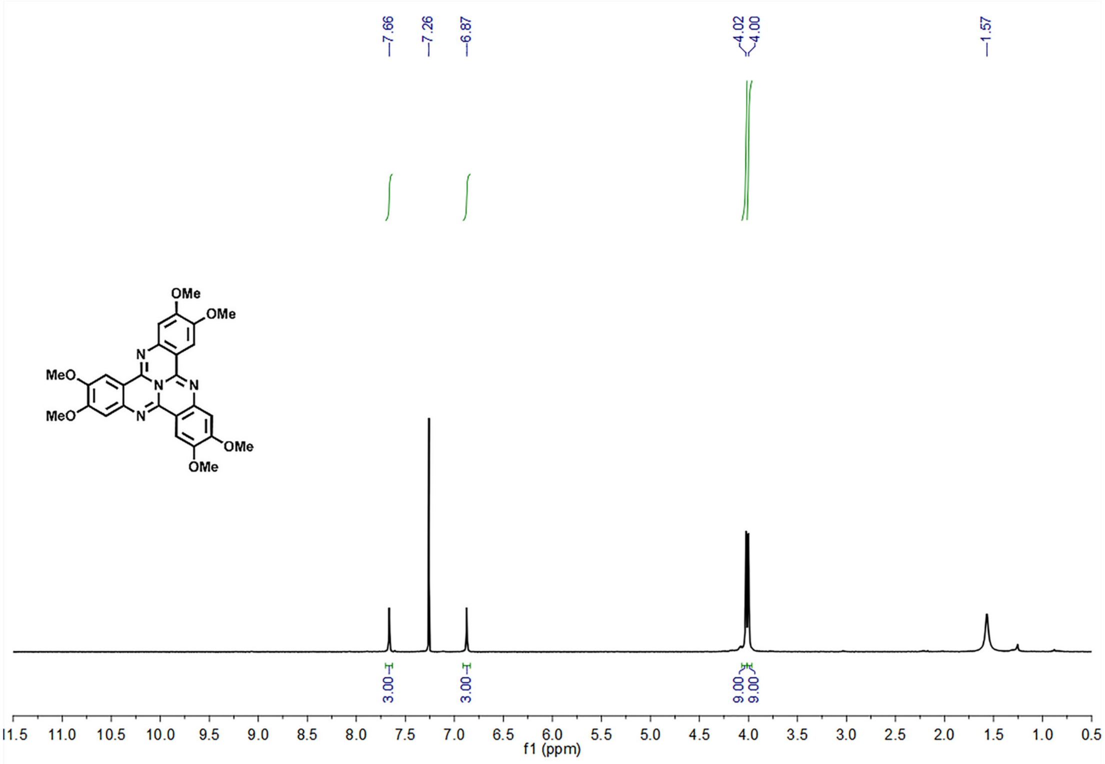

Figure S2. ^{1}H NMR spectrum ($CDCl_3$) of 2,3,7,8,12,13-Hexamethoxytricycloquinazoline.

Synthesis of H_6HHTQ ligand

2,3,7,8,12,13-hexamethoxytricycloquinazoline (2.3 g, 0.005 mol) and pyridine hydrochloride (76.9 g, 0.67 mol) were placed into the flask, heated at 513 K for 3 h in an inert atmosphere, and cooled down to 298 K. Water was added to the mixture, and the resulting black solid was collected by centrifugation. The solid was washed with water several times and dried under vacuum.

Yield: 1.58 g (83.3%).

^{1}H NMR (DMSO-d_6): 9.95 (s, 3H), 9.60 (s, 3H), 7.58 (s, 3H), 6.70 (s, 3H) ppm. ^{13}C NMR (DMSO-d_6): 151.74, 145.26, 143.49, 139.29, 112.21, 110.73, 109.38 ppm.

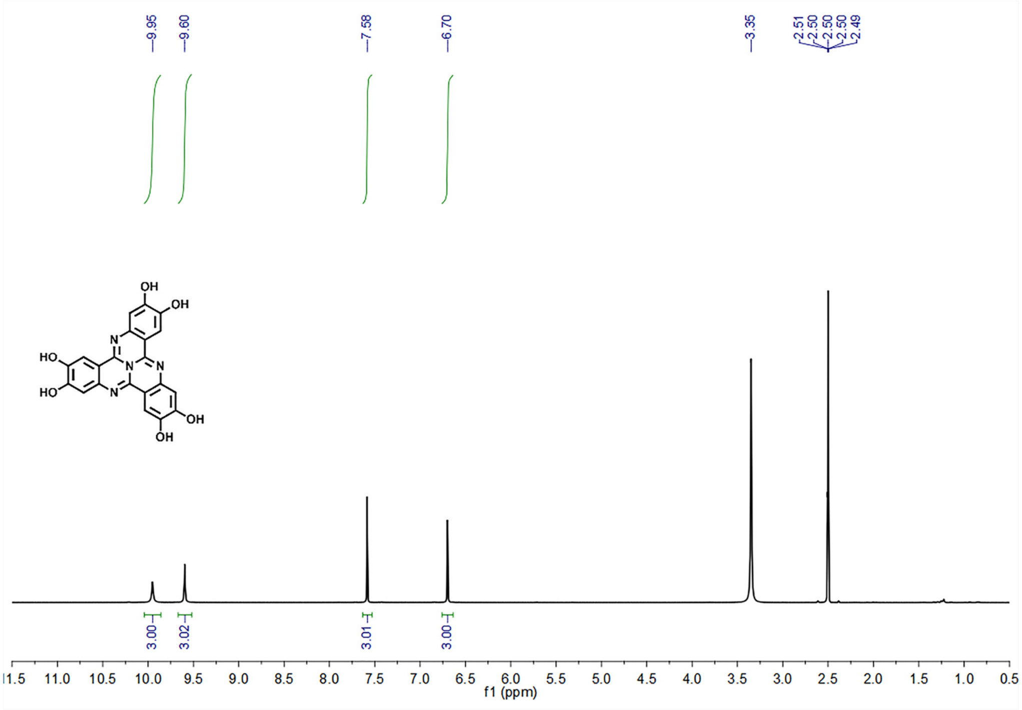

Figure S3. ^{1}H NMR spectrum (DMSO-d_6) of HHTQ ligand.

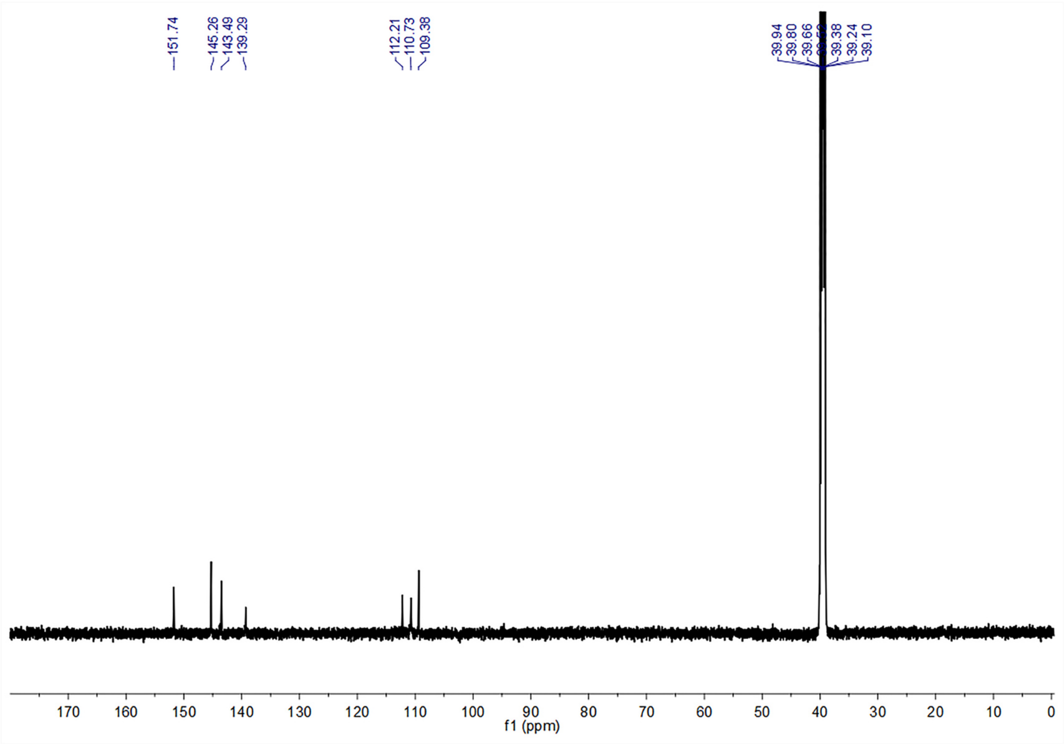

Figure S4. ^{13}C NMR spectrum (DMSO-d$_6$) of HHTQ ligand.

6. Synthesis of 2D c-MOFs

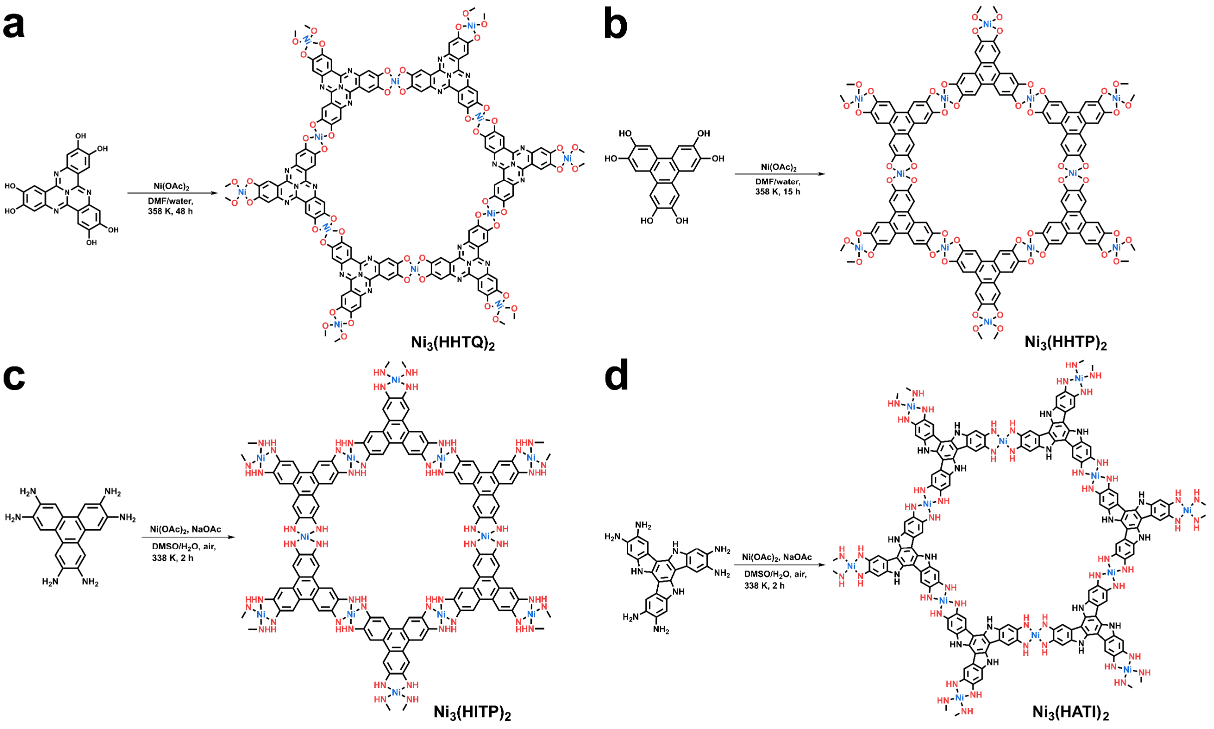

Figure S5. The synthetic schemes for (a) $Ni_3(HHTQ)_2$, (b) $Ni_3(HHTP)_2$, (c) $Ni_3(HITP)_2$, and (d) $Ni_3(HATI)_2$.

6.1 Synthesis of $Ni_3(HHTQ)_2$

$Ni(OAc)_2{\cdot}4H_2O$ (15 mg, 2.5 eq) and HHTQ (10 mg, 1 eq) were dissolved in a mixture of 0.2 mL DMF and 0.8 mL H_2O. The mixture was sonicated for 5 min and then heated in a glass vial at 358 K for 48 h. The resulting product was cooled down to 298 K, filtered, and washed with a large amount of water, DMF, and acetone, and then dried under vacuum at 298 K for 12 h.

Yield: 9.9 mg, 82.5%.

6.2 Synthesis of $Ni_3(HHTP)_2$

$Ni(OAc)_2·4H_2O$ (10 mg, 2.0 eq) and HHTP (6.5 mg, 1 eq) were dissolved in a mixture containing DMF (0.5 mL) and H_2O (0.5 mL). The mixture was sonicated for 5 min, heated in a glass reaction tube at 358 K for 15 h, cooled down to 298 K, filtered, washed with a large amount of water, DMF, and acetone, and dried under vacuum at 298 K for 12 h.

Yield: 6.9 mg, 84.9%.

6.3 Synthesis of $Ni_3(HITP)_2$ (HITP = 2,3,6,7,10,11-hexaiminotriphenylene)

$Ni(OAc)_2·4H_2O$ (3.5 mg, 1.5 eq) and NaOAc (11.5 mg, 15 eq) were added to 1.5 ml DMSO and preheated to 338 K. To this solution, HITP·6HCl (5.0 mg, 1.0 eq) in water (1.5 mL) was added. The mixture was heated in a glass vial at 338 K for 2 h under stirring, and then cooled down to 298 K. The product was filtered, washed with a large amount of water, DMSO, and acetone, and then dried under vacuum at 298 K for 12 h.

Yield: 3.6 mg, 57.2%.

6.4 Synthesis of $Ni_3(HATI)_2$ (HATI = 2,3,7,8,12,13-hexaiminotriindole)

The $Ni_3(HATI)_2$ MOF was synthesized following the reported procedure.[4]

$Ni(OAc)_2·4H_2O$ (2.4 mg, 1.5 eq) and NaOAc (88 mg, 150 eq) in DMSO/H_2O (v/v = 3:2, 4 mL in total) were preheated to 338 K. To this solution, HATI·6HCl (5 mg, 1 eq) DMSO (1 mL) was added. The mixture was heated in a 10 mL open glass vial for 2 h at 338 K under stirring. The resulting black powder was filtered, washed with a large amount of water, DMF, and acetone, and dried under vacuum at 298 K for 12 h.

Yield: 3.5 mg, 58.9%.

7. Rietveld refinement

The reported structures of $Ni_3(HHTQ)_2$, $Ni_3(HATI)_2$ and $Ni_3(HITP)_2$ frameworks were refined against PXRD data using Reflex tool of Materials Studio 5.0 software (Acellrys Inc., San Diego, CA, USA 2009). The Rietveld refinement was conducted using force-field supported refinement procedure (force field contribution 1%). In order to improve the data-to-parameter ratio, the rigid bodies were defined, namely metal ions and planar condensed aromatic rings. The broadening of the reflection at 2θ = 28° in the case of $Ni_3(HATI)_2$ structure may be explained by the stacking irregularities in the structure. In the case of $Ni_3(HHTP)_2$, the convergence was less satisfiable when using the earlier reported orthorhombic structure.[6] Therefore, we also used the hexagonal structure with AB stacking as the starting model, which results in a better convergence. The Rietveld plots are given in Figures S6 - S8.

Structural data for $Ni_3(HHTQ)_2$:

$C_{42}H_{12}N_8O_{12}Ni_3$, M = 996.68 g mol^{-1}, Hexagonal, $P\bar{6}2m$ (no. 187), a = 25.711(18) Å, c = 3.3352(34) Å, V = 1909.4(1) Å^3, Z = 1, λ = 1.54059 Å, T = 296 K, $2\theta_{range}$ = 2 - 50°, profile function Pseudo-Voigt, U = 0.01455, V = 0.73389, W = 0.07984, NA = 0.94913, refined motion groups/degree of freedom 2/2, R_p = 0.0273, and R_{wp} = 0.0364.

Structural data for $Ni_3(HHTP)_2$

$C_{12}H_4NiO_4$, M = 270.84 g mol^{-1}, hexagonal, $P6_3/mmc$ (no. 194), a = 21.669(18) Å, c = 6.658(2) Å, V = 2707.4(7) Å^3, Z = 6, λ = 1.54059 Å, T = 296 K, $2\theta_{range}$ = 3° - 50°, profile function Pseudo-Voigt, U = 0.64552, V = 0.40841, W = 0.17607, NA = 0.93343, refined motion groups/degree of freedom 3/3, R_p = 0.0448, and R_{wp} = 0.0685.

Structural data for $Ni_3(HITP)_2$

$C_{36}H_{24}N_{12}Ni_3$, M = 800.74 g mol^{-1}, orthorhombic, $Cmcm$ (no. 63), a = 37.425(30) Å, b = 21.182(36) Å, c = 6.533(45) Å, V = 5179.5(8) Å^3, Z = 3, λ = 1.54059 Å, T = 296 K, $2\theta_{range}$ = 3° - 50°, profile function Pseudo-Voigt, U = 0.21076, V = 0.16721, W = 0.14178, NA = 0.75114, refined motion groups/degree of freedom 4/11, R_p = 0.0456, and R_{wp} = 0.0638.

Structural data for $Ni_3(HATI)_2$

$C_{16}H_{10}N_6Ni$, $M = 344.99$ g mol^{-1}, hexagonal, *P6/m* (no. 175), $a = 26.670(40)$ Å, $c = 4.134(66)$ Å, $V = 2546.6(5)$ Å^3, $Z = 3$, $\lambda = 1.54059$ Å, $T = 296$ K, $2\theta_{range} = 3°$- 50°, profile function Pseudo-Voigt, $U = 0.01040$, $V = 0.71247$, $W = 0.27084$, $NA = 0.49967$, refined motion groups/degree of freedom 3/4, $R_p = 0.0369$, and $R_{wp} = 0.0568$.

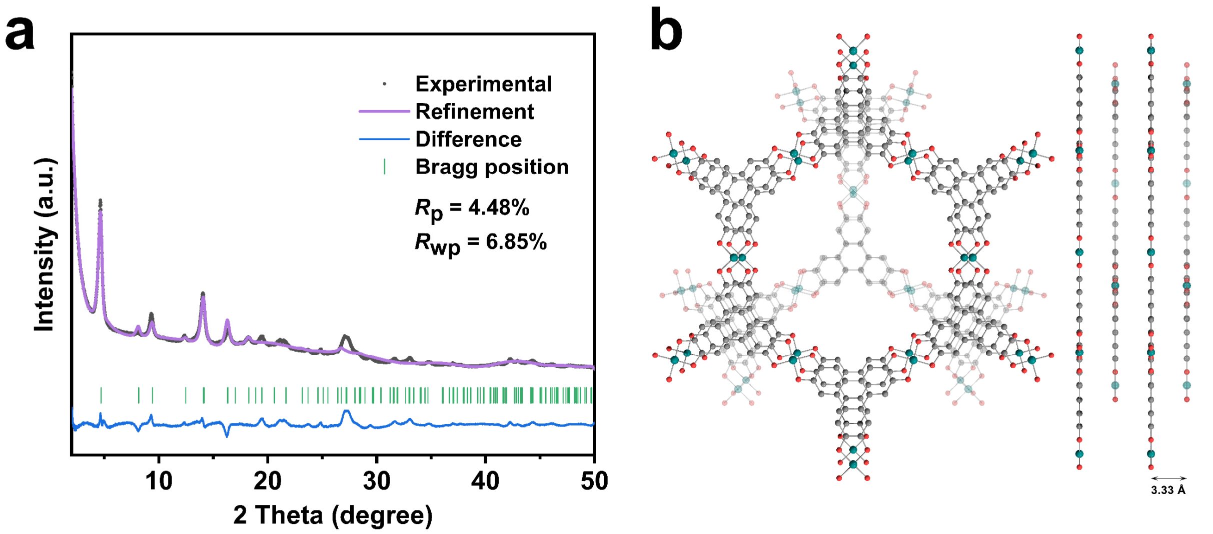

Figure S6. (a) Rietveld plot for hexagonal $Ni_3(HHTP)_2$, (R_p = 4.48% and R_{wp} = 6.85%) and (b) The crystal structure of $Ni_3(HHTP)_2$ along *c* axis. Red, grey, and green spheres represent O, C, and Ni atoms, respectively. Hydrogen atoms are omitted for clarity.

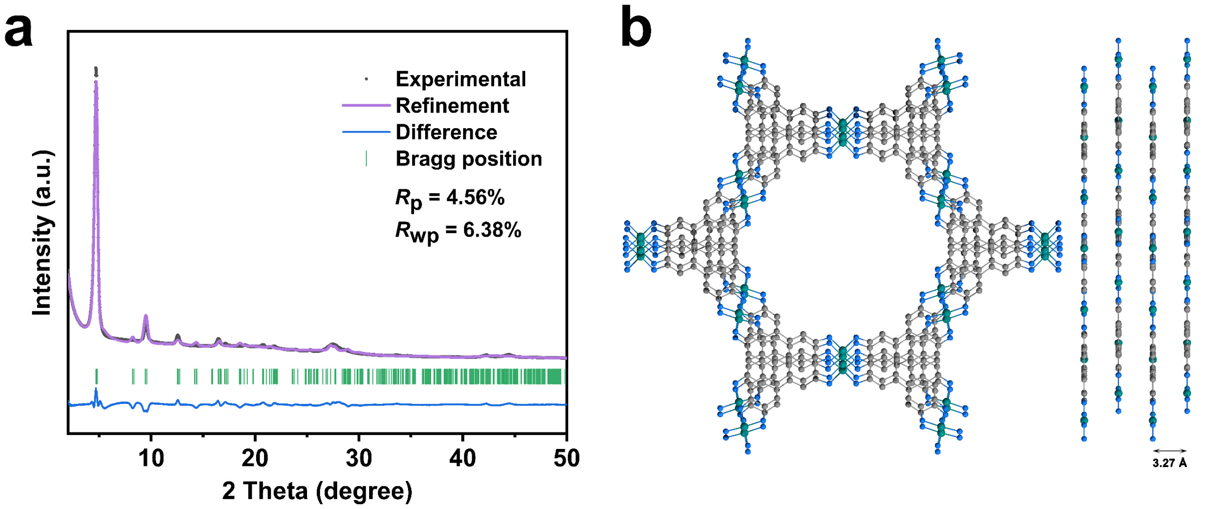

Figure S7. (a) Rietveld plot for $Ni_3(HITP)_2$, (R_p = 4.56% and R_{wp} = 6.38%) and (b) The crystal structure of $Ni_3(HITP)_2$ along *c* axis. Grey, blue, and green spheres represent C, N, and Ni atoms, respectively. Hydrogen atoms are omitted for clarity.

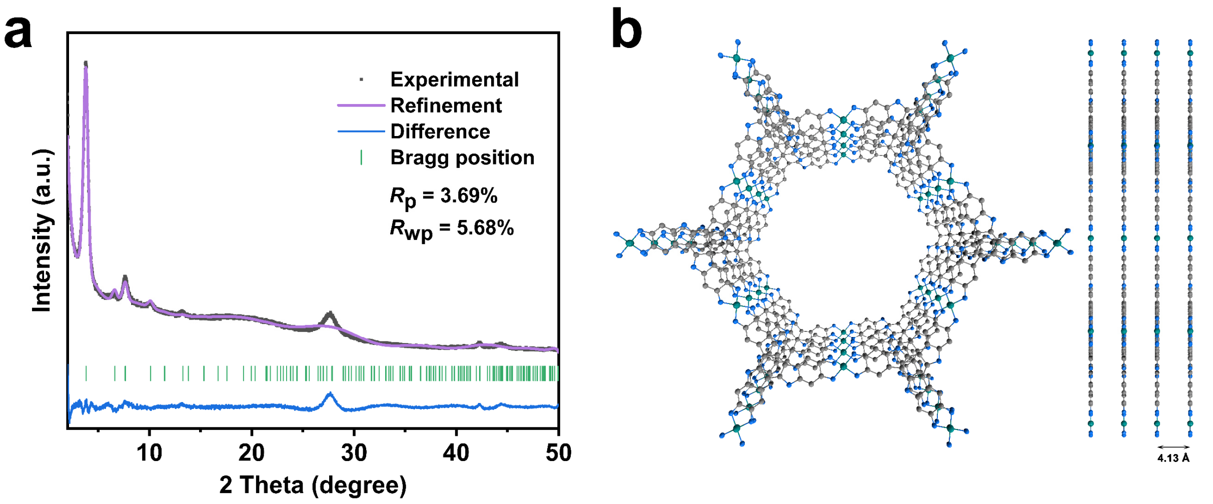

Figure S8. (a) Rietveld plot for $Ni_3(HATI)_2$, (R_p = 3.69% and R_{wp} = 5.68%) and (b) The crystal structure of $Ni_3(HATI)_2$ along *c* axis. Grey, blue, and green spheres represent C, N, and Ni atoms, respectively. Hydrogen atoms are omitted for clarity.

Table S1. Unit cell parameters and atomic coordinates for $Ni_3(HHTQ)_2$.

$Ni_3(HHTQ)_2$				
Hexagonal, $P\bar{6}2m$, a = 25.711(18) Å, c = 3.3352(34) Å, V = 1909.4(1) Å^3, Z = 1				
**Atom**	***x***	***y***	***z***	***Atom site occupancy***
Ni1	0.00000	0.48673	1/2	1.00
O1	0.05480	0.46293	1/2	1.00
O2	0.05921	0.56618	1/2	1.00
N1	0.27245	0.55997	1/2	1.00
N2	1/3	2/3	1/2	1.00
C1	0.11192	0.51334	1/2	1.00
C2	0.16470	0.50979	1/2	1.00
C3	0.22108	0.56304	1/2	1.00
C4	0.11444	0.56818	1/2	1.00
C5	0.17023	0.62209	1/2	1.00
C6	0.22442	0.61977	1/2	1.00
C7	0.28220	0.67191	1/2	1.00

Table S2. Unit cell parameters and atomic coordinates for $Ni_3(HHTP)_2$.

$Ni_3(HHTP)_2$				
hexagonal, $P6_3/mmc$, a = 21.669(18) Å, c = 6.658(2) Å, V = 2707.4(7) Å^3, Z = 6				
**Atom**	***x***	***y***	***z***	***Atom site occupancy***
Ni1	1.16520	0.83480	3/4	1.00
O1	1.39992	1.25872	1/4	1.00
O2	2.26431	1.07340	1/4	1.00
C1	2.06628	1.00027	1/4	1.00
C2	1.99675	0.86652	1/4	1.00
C3	2.19766	1.06779	1/4	1.00
C4	1.46660	1.26435	1/4	1.00

C5	1.33193	0.59808	3/4	1.00
C6	1.20190	0.53083	3/4	1.00

Table S3. Unit cell parameters and atomic coordinates for $Ni_3(HITP)_2$.

**$Ni_3(HITP)_2$**				
orthorhombic, Cmcm, a = 37.425(30) Å, b = 21.182(36) Å, c = 6.533(45) Å, V = 5179.5(8) Å^3, Z = 3				
**Atom**	***x***	***y***	***z***	***Atom site occupancy***
Ni1	-0.25657	-0.68886	5/4	1.00
Ni2	-1/2	-0.96309	5/4	1.00
N1	-0.30309	-0.71608	5/4	1.00
N2	-0.24568	-0.76904	5/4	1.00
N3	-0.46684	-0.90467	5/4	1.00
N4	-0.46352	-1.01727	5/4	1.00
N5	-0.28915	-1.16841	5/4	1.00
N6	-0.23464	-1.10662	5/4	1.00
C1	-0.26413	-1.07166	5/4	1.00
C2	-0.26697	-1.00782	5/4	1.00
C3	-0.29996	-0.97848	5/4	1.00
C4	-0.33197	-1.01506	5/4	1.00
C5	-0.32914	-1.07895	5/4	1.00
C6	-0.29608	-1.10809	5/4	1.00
C7	-0.30251	-0.90853	5/4	1.00
C8	-0.36661	-0.98483	5/4	1.00
C9	-0.36873	-0.91843	5/4	1.00
C10	-0.33650	-0.87804	5/4	1.00
C11	-0.40207	-0.89047	5/4	1.00
C12	-0.43319	-0.92504	5/4	1.00
C13	-0.43126	-0.99122	5/4	1.00

C14	-0.39786	-1.01932	5/4	1.00
C15	-0.27171	-0.87296	5/4	1.00

Table S4. Unit cell parameters and atomic coordinates for $Ni_3(HATI)_2$.

**$Ni_3(HATI)_2$**				
hexagonal, P6/m, a = 26.670(40) Å, c = 4.134(66) Å, V = 2546.6(5) Å^3, Z = 3				
**Atom**	***x***	***y***	***z***	***Atom site occupancy***
Ni1	1	1/2	1/2	1.00
N1	0.55299	0.25466	3/2	1.00
N2	0.47399	0.04910	3/2	1.00
N3	0.56846	0.06270	3/2	1.00
C1	0.27362	0.62990	1/2	1.00
C2	0.64650	0.49569	3/2	1.00
C3	0.60472	0.39163	3/2	1.00
C4	0.48690	0.89564	3/2	1.00
C5	0.39159	0.70198	3/2	1.00
C6	0.65214	0.44828	3/2	1.00
C7	0.56958	0.11260	3/2	1.00
C8	0.61690	0.16708	3/2	1.00

8. Thermogravimetric Analysis

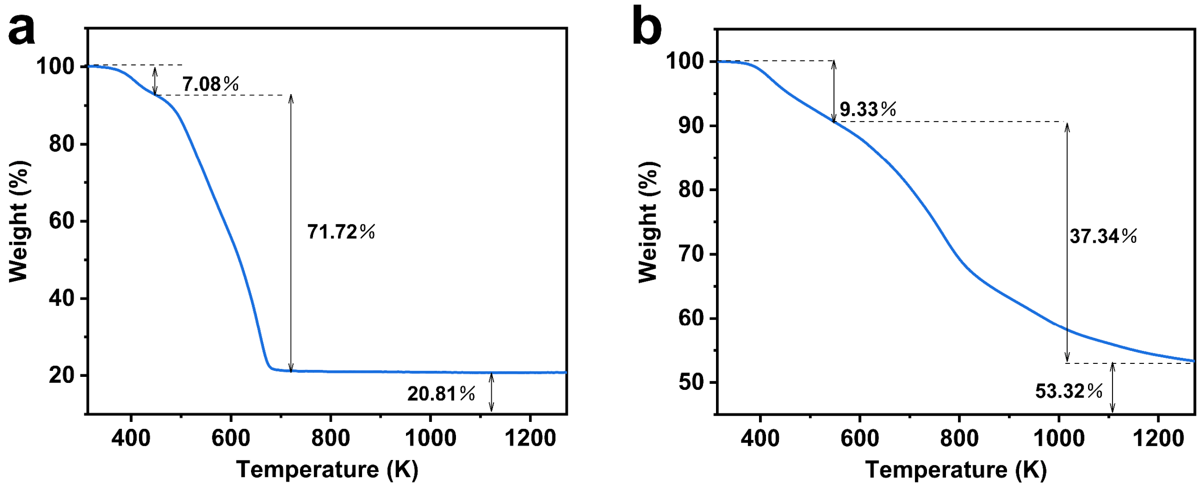

Figure S9. TGA curve of $Ni_3(HHTQ)_2$ in (a) air and (b) Ar.

9. Nitrogen adsorption

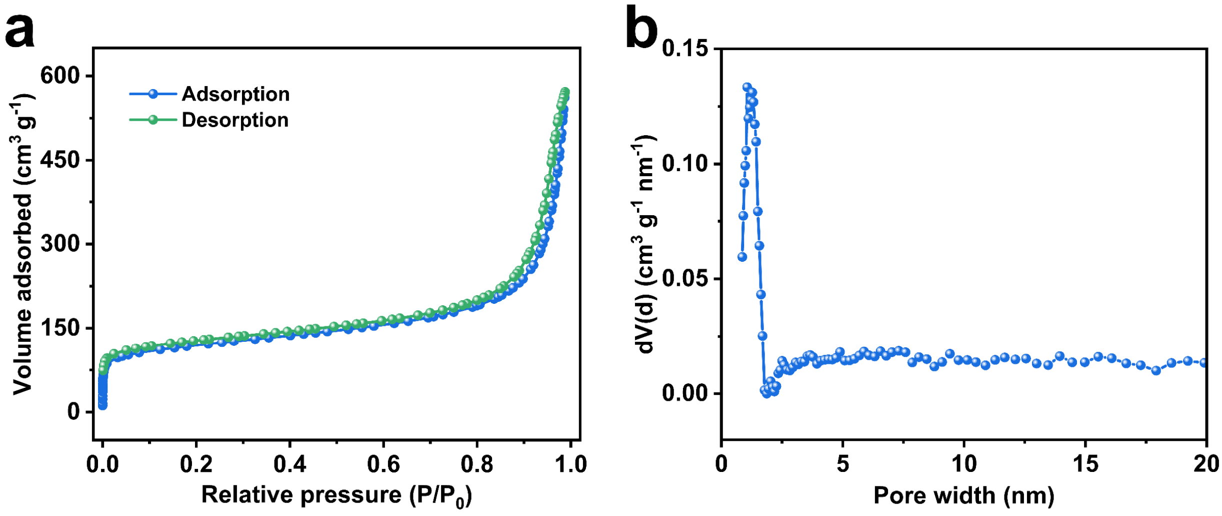

Figure S10. (a) N_2 adsorption-desorption isotherms of $Ni_3(HHTP)_2$ at 77 K, the BET surface area is 431 m^2 g^{-1}. (b) The pore size distribution, the pore size peak is centred at 1.2 nm.

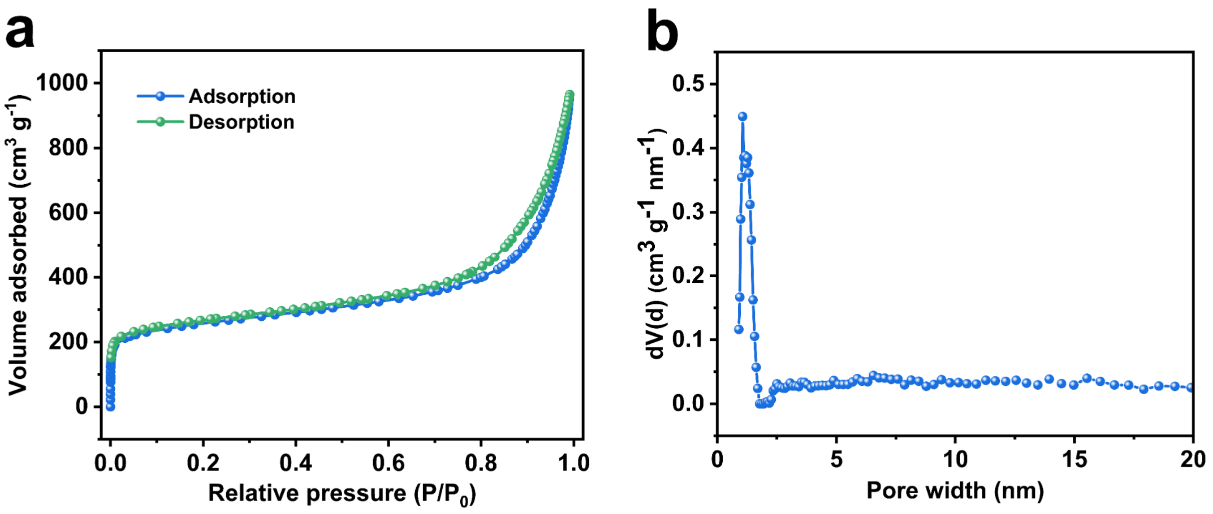

Figure S11. (a) N_2 adsorption-desorption isotherms of $Ni_3(HITP)_2$ at 77 K, the BET surface area is 931 m^2 g^{-1}. (b) The pore size distribution, the pore size peak is centred at 1.1 nm.

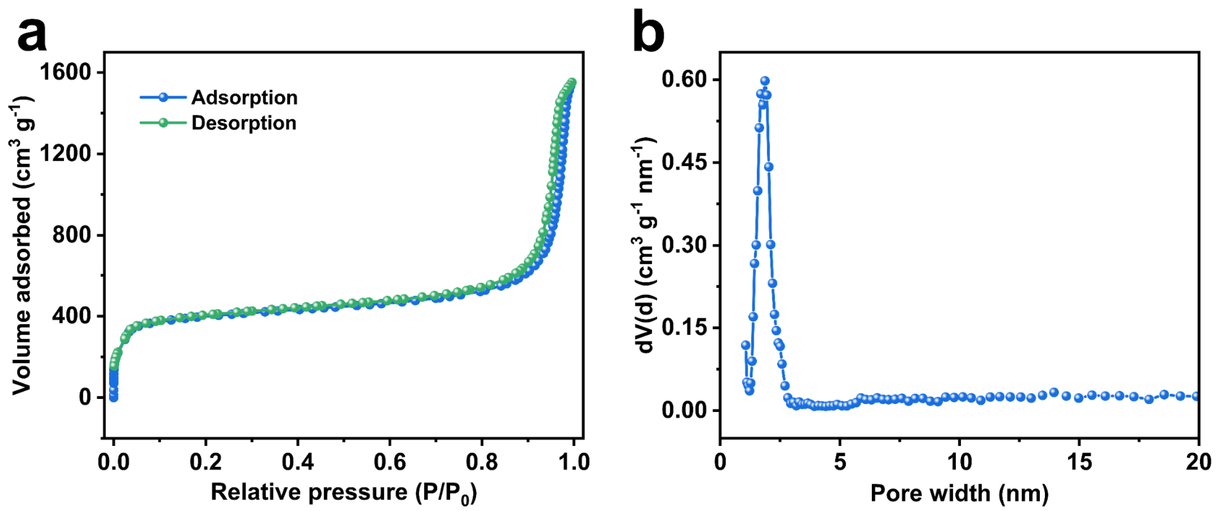

Figure S12. (a) N_2 adsorption-desorption isotherms of $Ni_3(HATI)_2$ at 77 K, the BET surface area is 1467 m^2 g^{-1}. (b) The pore size distribution, the pore size peak is centred at 1.8 nm.

Table S5. Surface characteristics of four *c*-MOFs.

Samples	$ECSA_{GOR}$ (mF cm^{-2})	$ECSA_{HER}$ (mF cm^{-2})	Surface area (m^2 g^{-1})	Pore volume (cc g^{-1})
$Ni_3(HHTQ)_2$	6.76	9.65	480	0.31
$Ni_3(HHTP)_2$	4.81	7.03	431	0.29
$Ni_3(HITP)_2$	4.06	6.04	931	0.62
$Ni_3(HATI)_2$	3.29	4.71	1467	0.81

10. Scanning electron microscopy (SEM) of synthetized MOFs

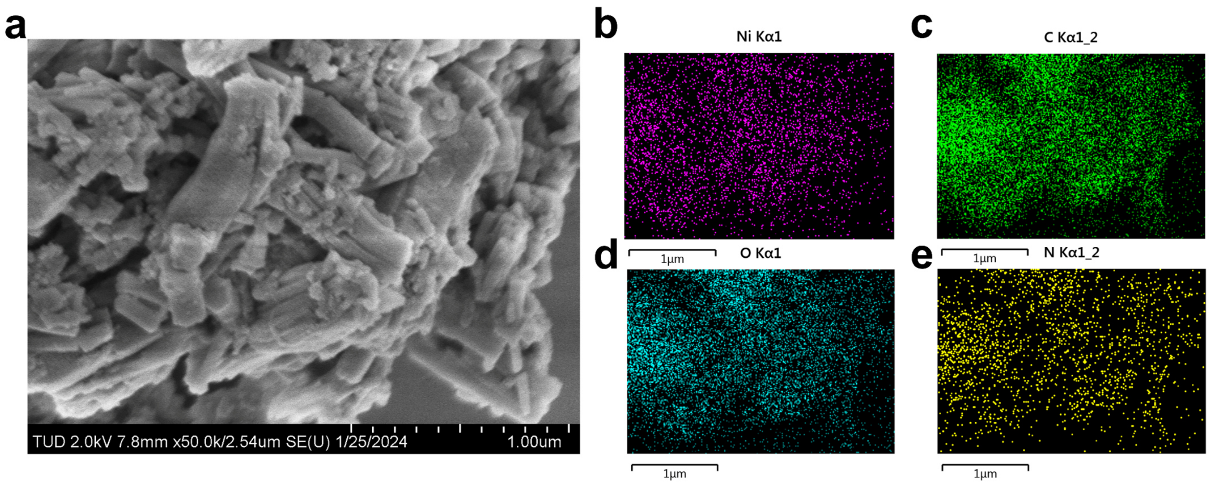

Figure S13. $Ni_3(HHTQ)_2$. (a) SEM image. (b-e) Corresponding EDS mapping images with respect to Ni, C, O, N.

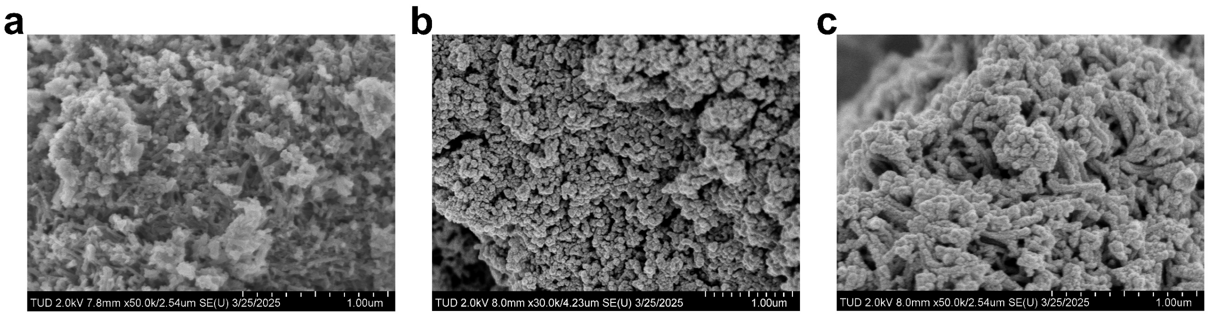

Figure S14. SEM images of (a)$Ni_3(HHTP)_2$, (b) $Ni_3(HITP)_2$, and (c) $Ni_3(HATI)_2$.

11. Preparation of MOF-based electrodes

10.0 mg of the as-prepared MOFs powder was added to the mixture of ethanol (0.8 mL), water (0.2 mL), and Nafion (5 wt%, 20 μL) and ultrasonicated for 30 min to form a homogeneous suspension. Then, the catalyst ink was drop cast onto the surface of carbon paper (10 × 20 mm) and dried. The corresponding loading of the catalyst was about 1 mg cm^{-2}.

12. Electrochemical characterizations

A Biologic VMP3 potentiostat was utilized for the electrochemical characterizations at 298 K. Electrochemical measurements in this work were mostly performed in a conventional three-electrode electrolytic cell, where the carbon paper with loaded with the corresponding catalyst was used as the working electrode, the platinum mesh was used as the counter electrode, and Hg/HgO was used as the reference electrode, respectively. A graphite rod was used as the counter electrode in hydrogen evolution reaction (HER) measurement. For the measurement of overall electrochemical performance toward glycerol oxidation reaction-HER (GOR-HER), a two-electrode electrolytic cell with a pair of $Ni_3(HHTQ)_2$ electrodes was used. The volume of electrolyte for electrochemical measurements was about 30 mL. All potential values reported in this work were referenced to the reversible hydrogen electrode (RHE) according to the following equation:[7]

$$E_{RHE} = E_{Hg/HgO} + 0.059 \times pH + 0.098$$

Linear sweep voltammetry (LSV) was measured at a scan rate of 5 mV s^{-1}. Cyclic voltammetry (CV) was measured at a series of scan rates from 20 to 120 mV s^{-1}. The long-term GOR electrolysis was performed at the applied oxidation potentials of 1.30 to 1.55 V (vs. RHE) in a three-electrode electrolytic cell and at the applied voltages of 1.65 to 1.90 V in a two-electrode electrolytic cell for 8 h to determine the GOR products and calculate the corresponding FEs. The electrolyte volume and the surface area of the two-electrode system are the same as that of the three-electrode system. The concentrations of the liquid products were determined by ^1H NMR using DMSO as an internal standard.

During each ^{1}H NMR measurement, 100 μL D_2O and 2 μL DMSO were added to 500 μL electrolyte after the long-term GOR electrolysis.

13. Calculation of Faradaic efficiency (FE, %) for formate

According to the proposed GOR mechanistic scheme, the conversion from glycerol into formate under alkaline conditions can be described by the following chemical equation:

$$C_3H_8O_3 \text{ (glycerol)} + 11OH^- \rightarrow 3HCOO^- \text{ (formate)} + 8H_2O + 8e^-$$

Therefore, the FE of formate can be calculated by the following equation:

$$FE\ (\%) = \frac{\text{n(formate)}}{Q/\alpha F},$$

where Q stands for the total charge passed through the electrodes during the reaction process (C), F represents the Faradaic constant (96485 C mol^{-1}), and α is the number of electrons that produce 1 mol of formate during the GOR process (α = 8/3).

14. Drainage method for hydrogen production

The drainage method was used to verify the volume of hydrogen produced during the reaction process.[8] The volume of hydrogen can be determined by measuring the volume of electrolyte in a graduated cylinder with different time intervals at a constant applied potential. Moreover, combining the Faraday's law and ideal gas law, the following equation can be used to determine the theoretical volume of hydrogen production:

$$V_{theo,H_2} = \frac{QRT}{\alpha PF},$$

where Q stands for the total charge passed through the electrodes during the reaction process (C), R represents the ideal gas constant (8.314 J K^{-1} mol^{-1}), T is the working temperature (K), α is the number of electrons needed to generate 1 mol H_2 (α = 2), P is the working pressure (Pa), and F is the Faradaic constant (96485 C mol^{-1}).

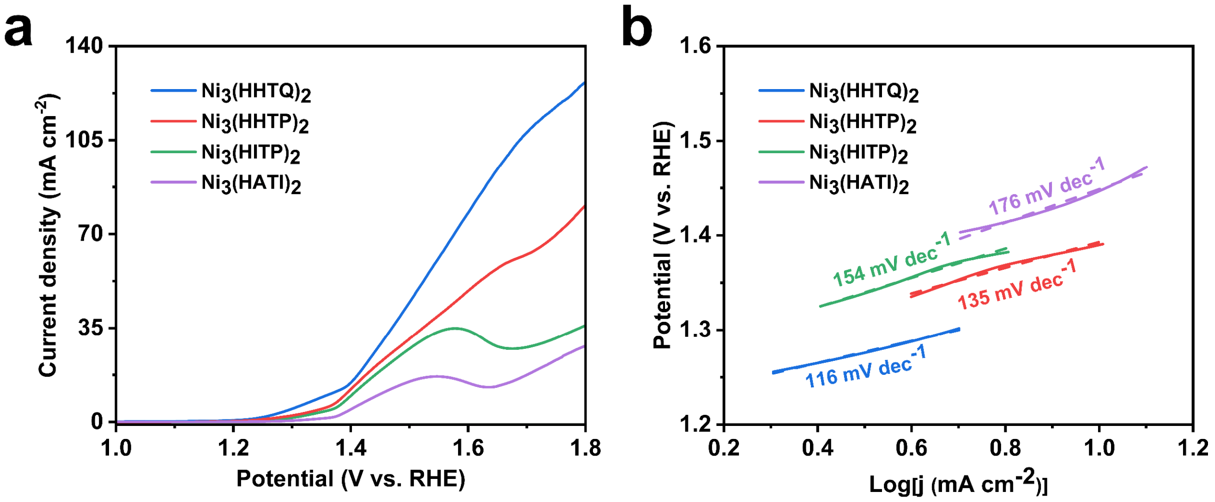

Figure S15. (a) LSV curves and (b) corresponding Tafel plots of various catalysts toward GOR.

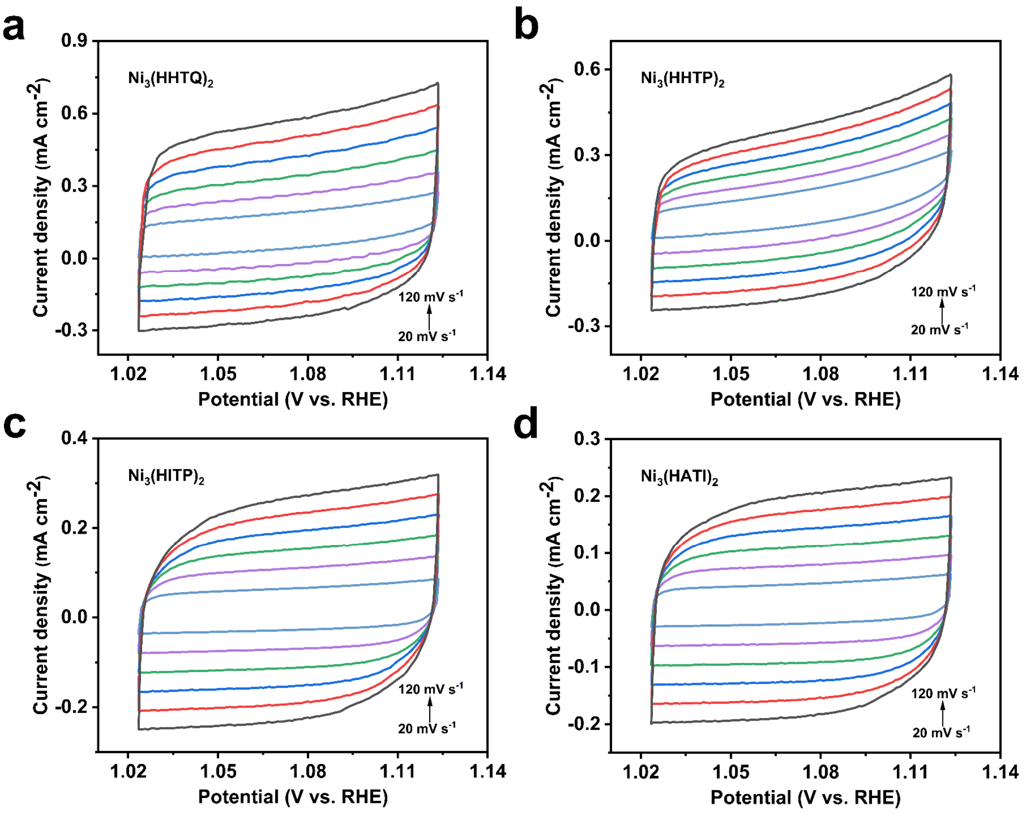

Figure S16. (a-d) CV curves of different catalysts in 1 M KOH with 0.1 M glycerol at non-Faradaic regions with various scan rates for $Ni_3(HHTQ)_2$, $Ni_3(HHTP)_2$, $Ni_3(HITP)_2$, and $Ni_3(HATI)_2$.

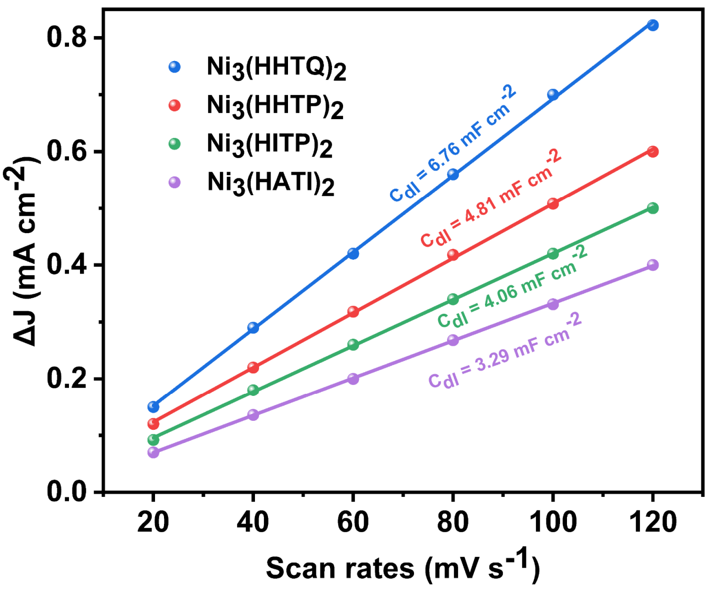

Figure S17. The current density differences (ΔJ) at 1.074 V (vs. RHE) of different catalysts plotted against the scan rate for GOR.

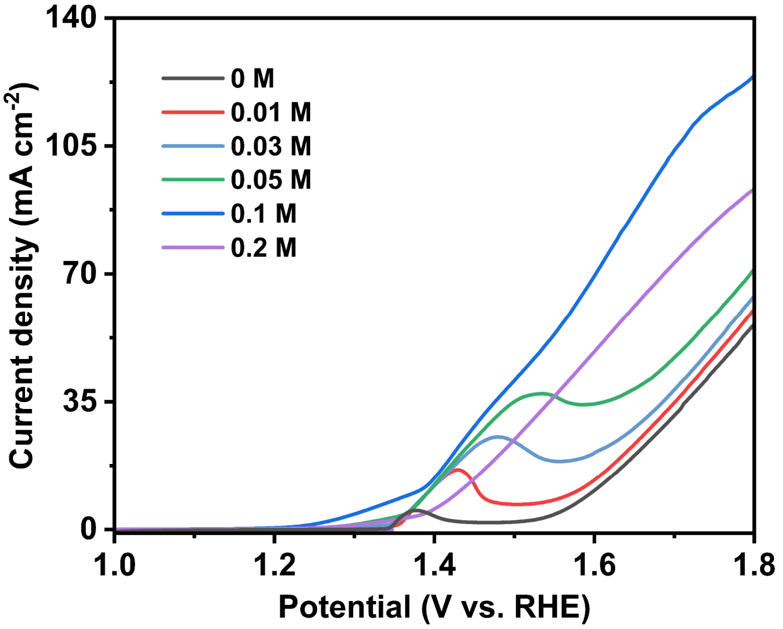

Figure S18. LSV curves of $Ni_3(HHTQ)_2$ in 1.0 M KOH with varied concentrations of glycerol. The molar ratio between Ni in $Ni_3(HHTQ)_2$ and glycerol are 0.02 for 0.01 M glycerol, 6.67×10^{-3} for 0.03 M glycerol, 4×10^{-3} for 0.05 M glycerol, 2×10^{-3} for 0.1 M glycerol, and 1×10^{-3} for 0.1 M glycerol, respectively.

15. Powder X-ray diffraction (PXRD)

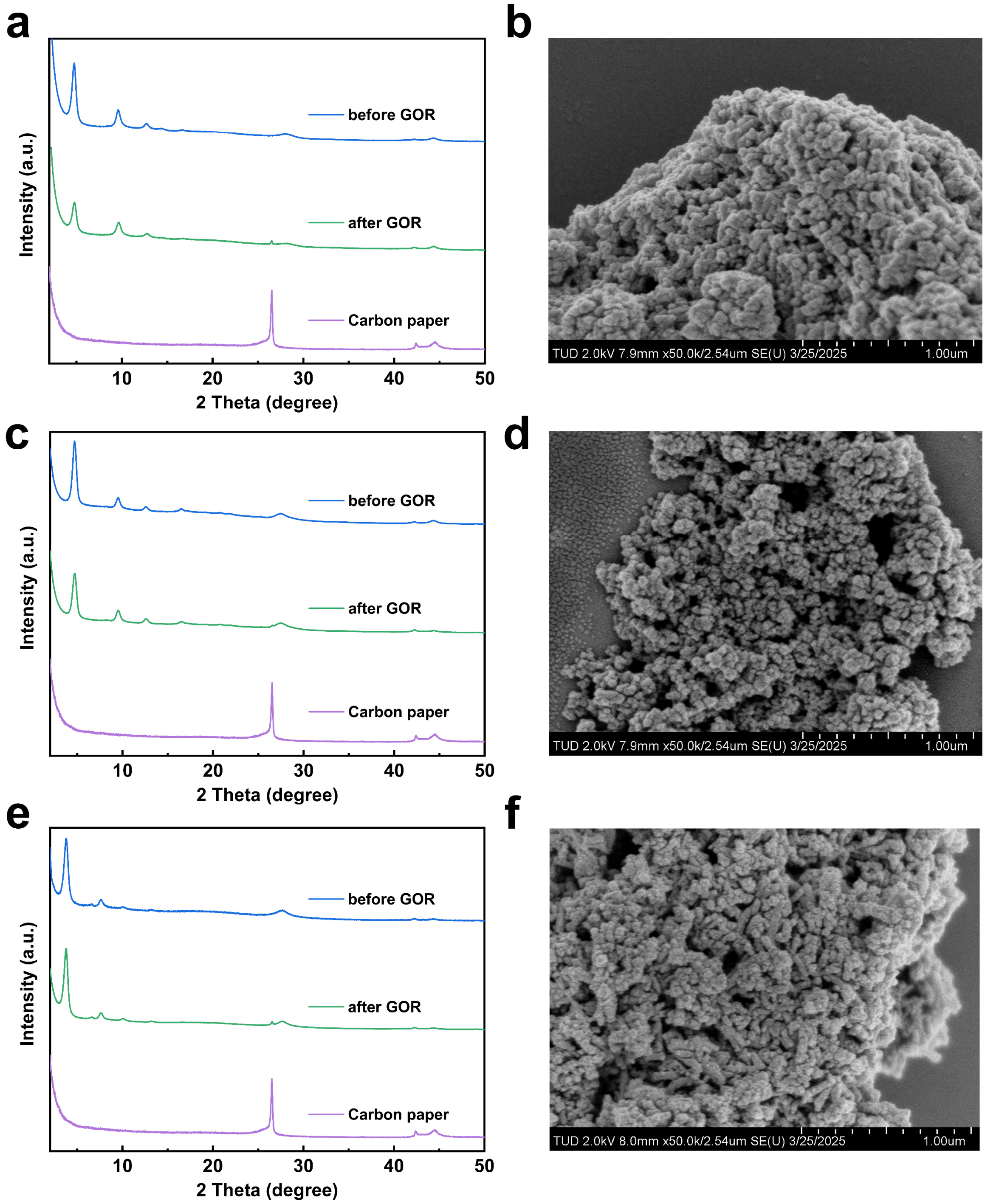

Figure S19. PXRD patterns of (a) $Ni_3(HHTP)_2$, (c) $Ni_3(HITP)_2$, and (e) $Ni_3(HATI)_2$ before and after GOR test. SEM images of (b) $Ni_3(HHTP)_2$ (d) $Ni_3(HITP)_2$, (f) $Ni_3(HATI)_2$ after GOR test.

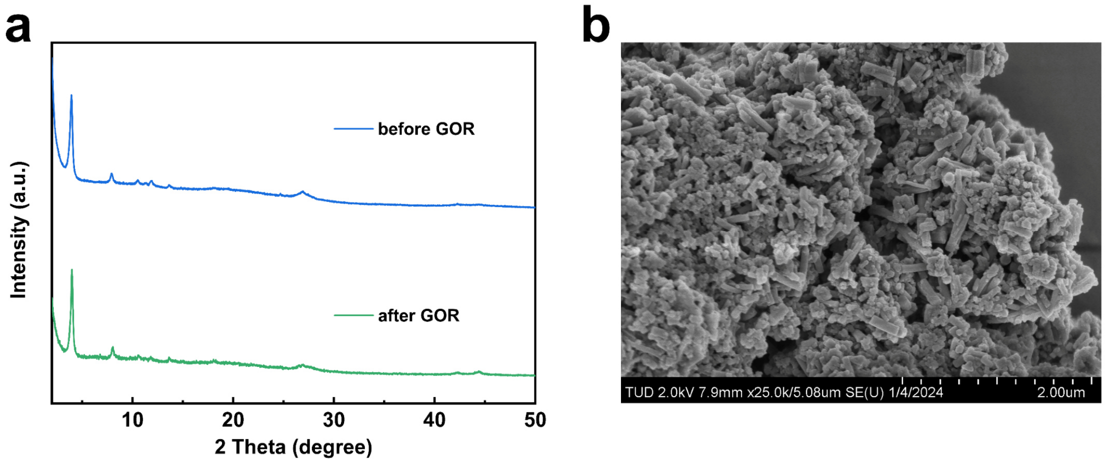

Figure S20. (a) PXRD patterns of $Ni_3(HHTQ)_2$ before and after GOR test. (b) SEM images of $Ni_3(HHTQ)_2$ after GOR test.

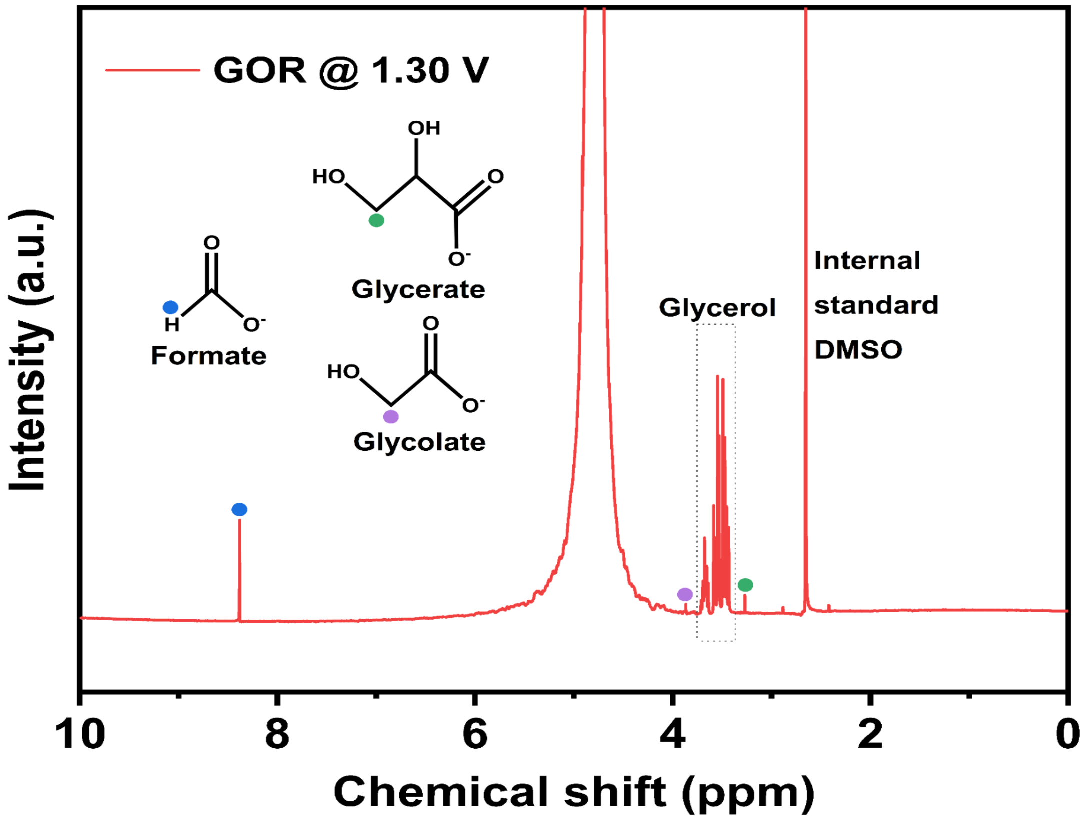

Figure S21. The high-resolution ^{1}H NMR spectra of the products for $Ni_3(HHTQ)_2$ at 1.30 V for 8h.

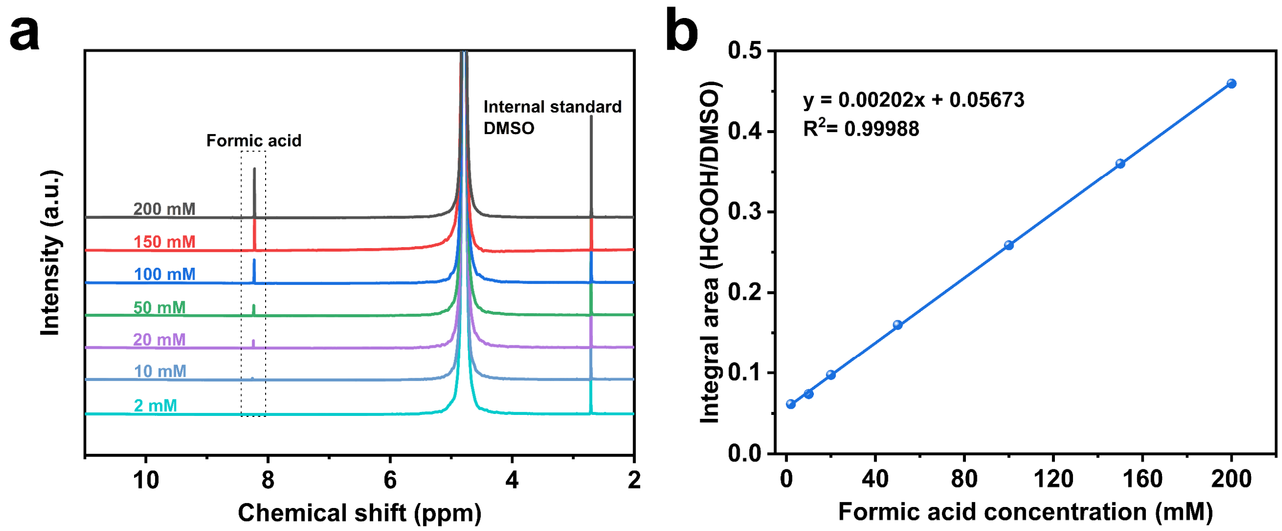

Figure S22. (a) ^{1}H NMR spectra for a series of standard formic acid solution. (b) The ^{1}H NMR calibration curve for formic acid.

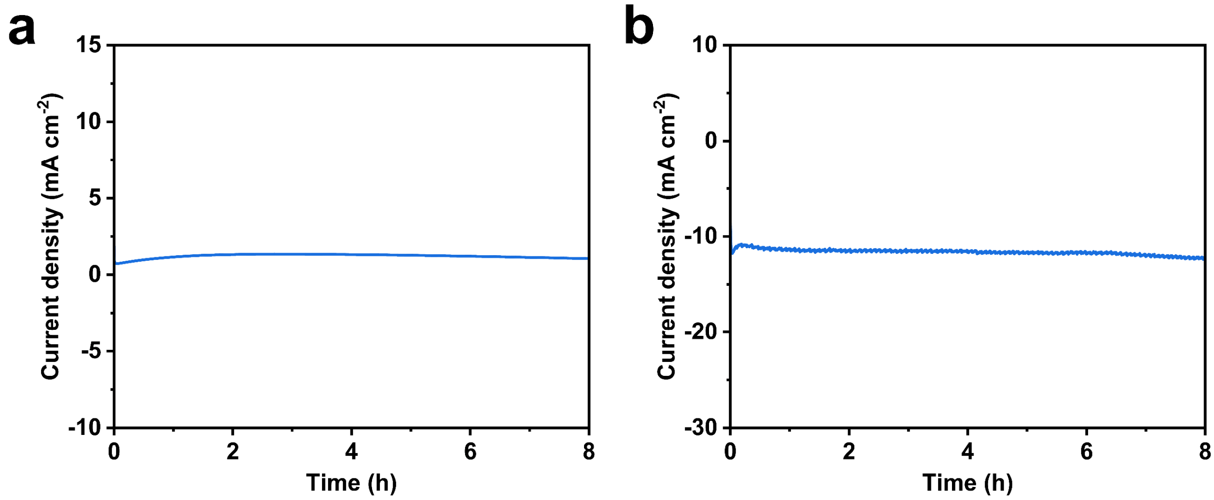

Figure S23. Chronoamperometry curves of $Ni_3(HHTQ)_2$ over 8 h for (a) GOR in 1.0 M KOH with 0.1 M glycerol, and (b) HER in 1.0 M KOH.

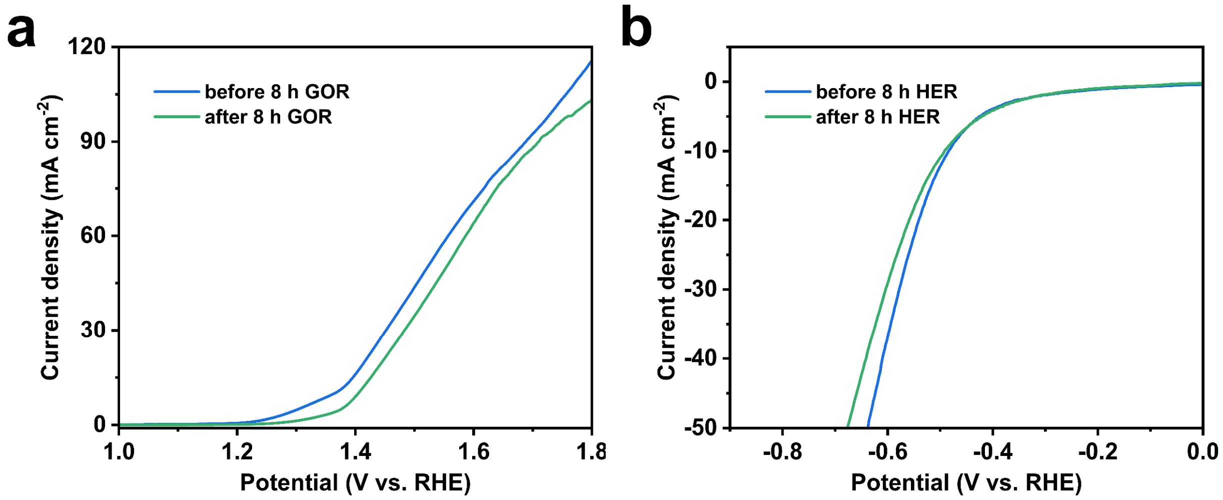

Figure S24. Long-term stability analysis of $Ni_3(HHTQ)_2$. (a) LSV curves before and after 8 h GOR. (b) LSV curves before and after 8 h HER.

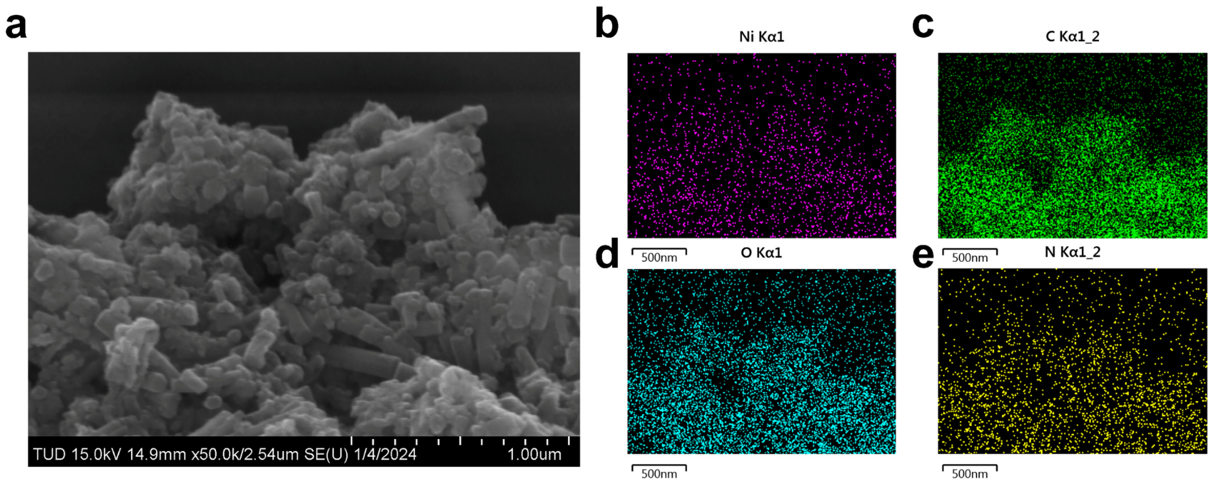

Figure S25. EDS mappings of $Ni_3(HHTQ)_2$ after GOR test. (a) SEM image. (b-e) corresponding EDS mapping images with respect to Ni, C, O, N.

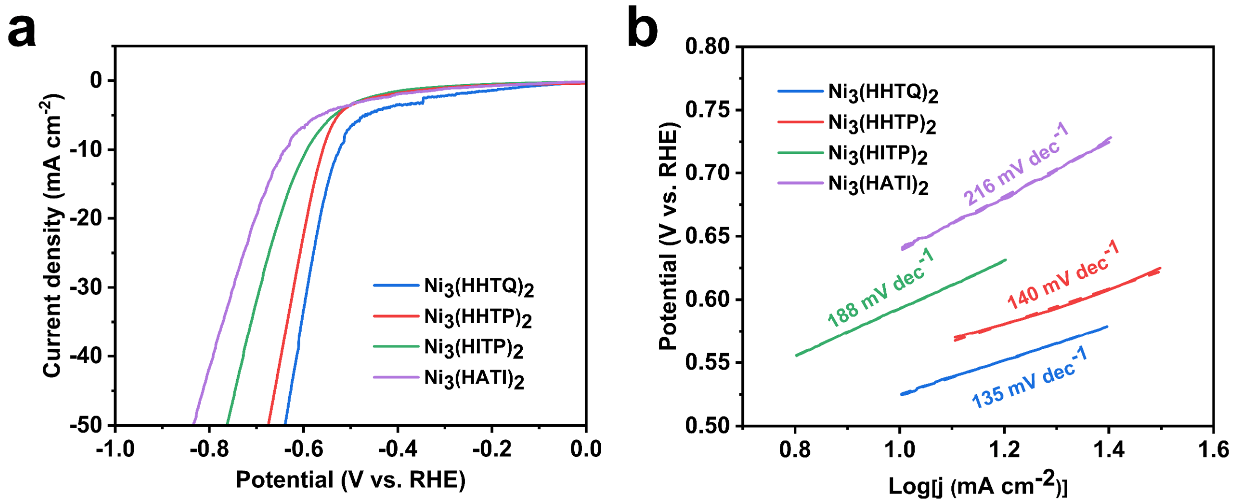

Figure S26. (a) LSV curves and (b) corresponding Tafel plots of various catalysts toward HER.

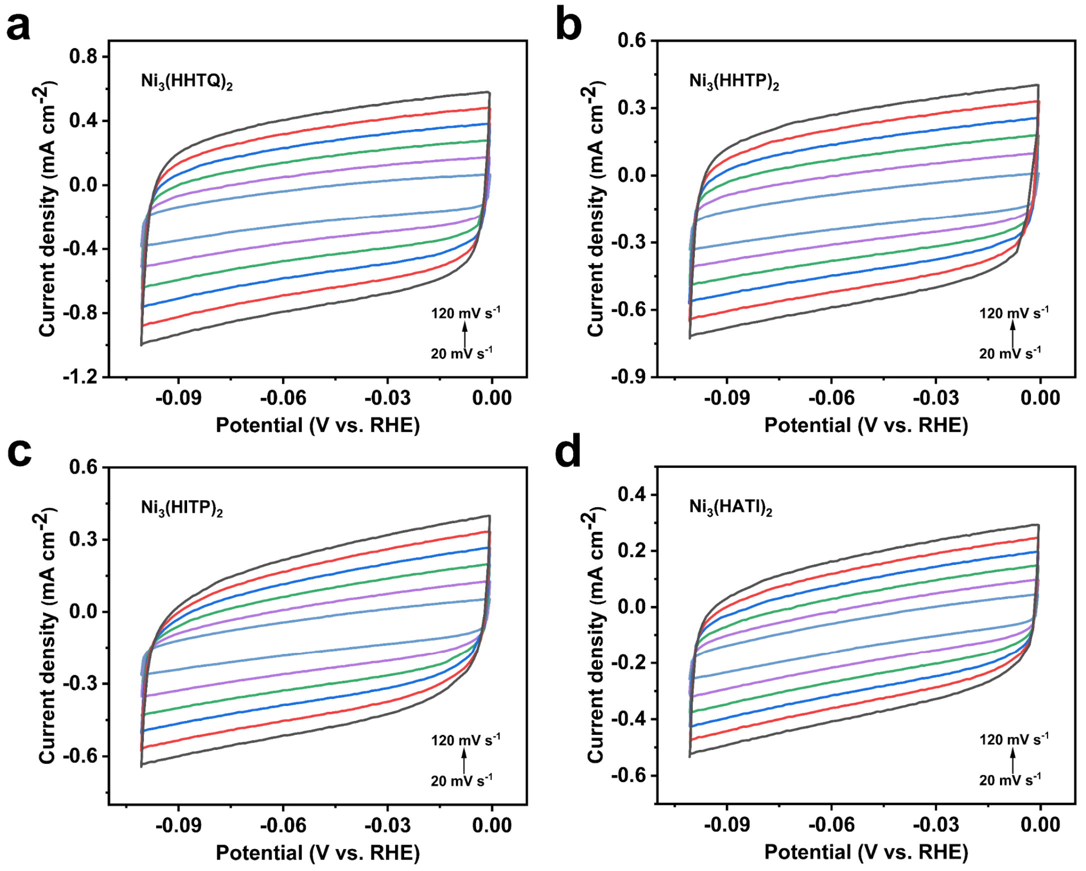

Figure S27. (a-d) CV curves of different catalysts in 1 M KOH at non-Faradaic regions with various scan rates for $Ni_3(HHTQ)_2$, $Ni_3(HHTP)_2$, $Ni_3(HITP)_2$, and $Ni_3(HATI)_2$.

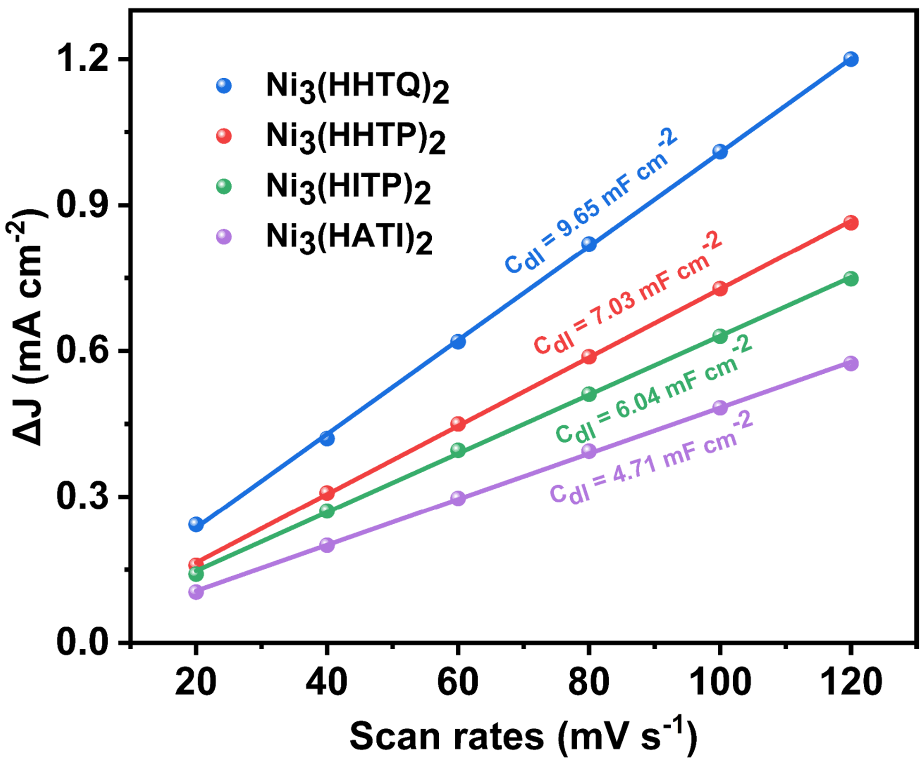

Figure S28. The current density differences (ΔJ) at -0.05 V (vs. RHE) of different catalysts plotted against the scan rate for HER.

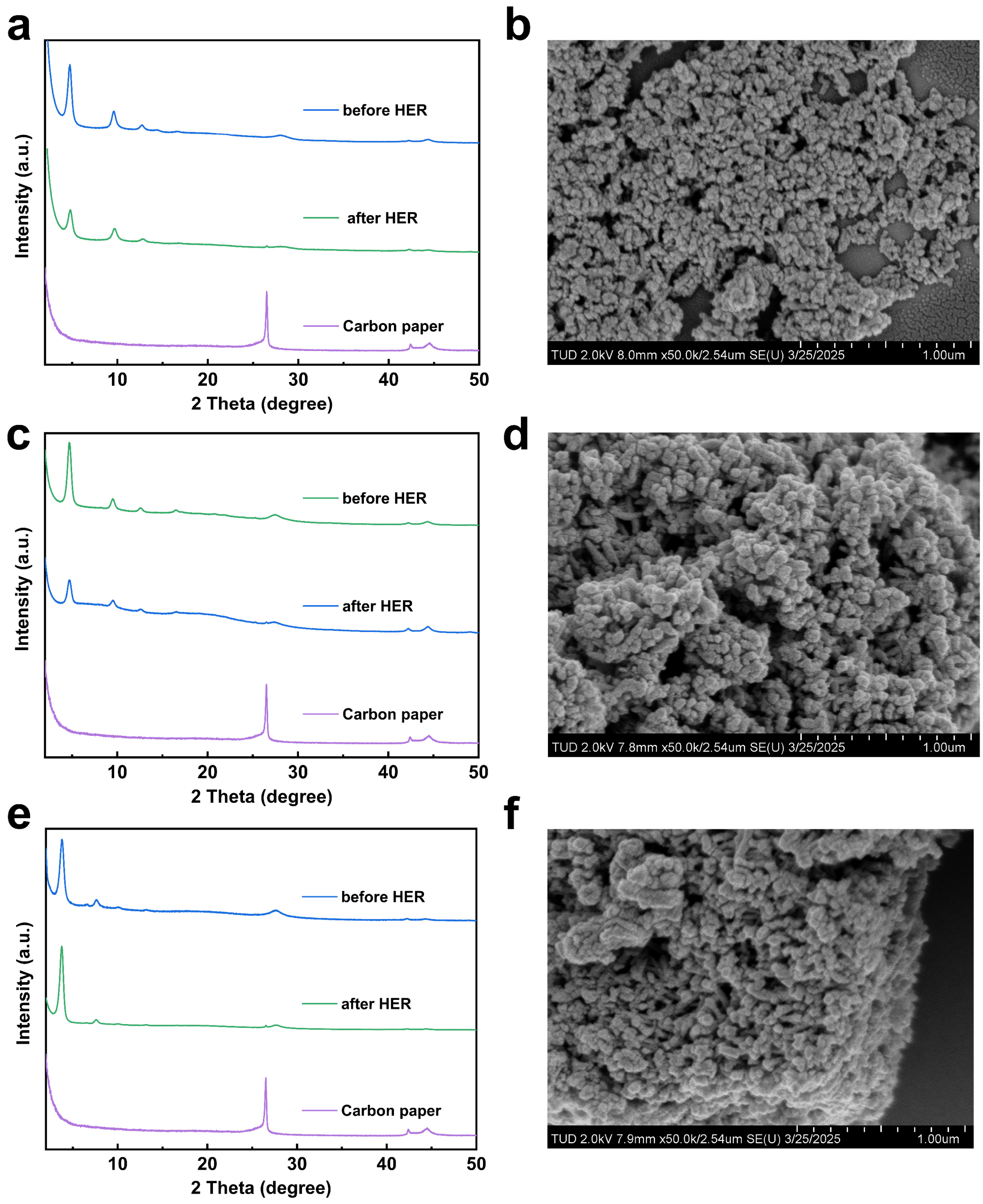

Figure S29. PXRD patterns of (a) $Ni_3(HHTP)_2$, (c) $Ni_3(HITP)_2$, and (e) $Ni_3(HATI)_2$ before and after HER test. SEM images of (b) $Ni_3(HHTP)_2$ (d) $Ni_3(HITP)_2$, (f) $Ni_3(HATI)_2$ after HER test.

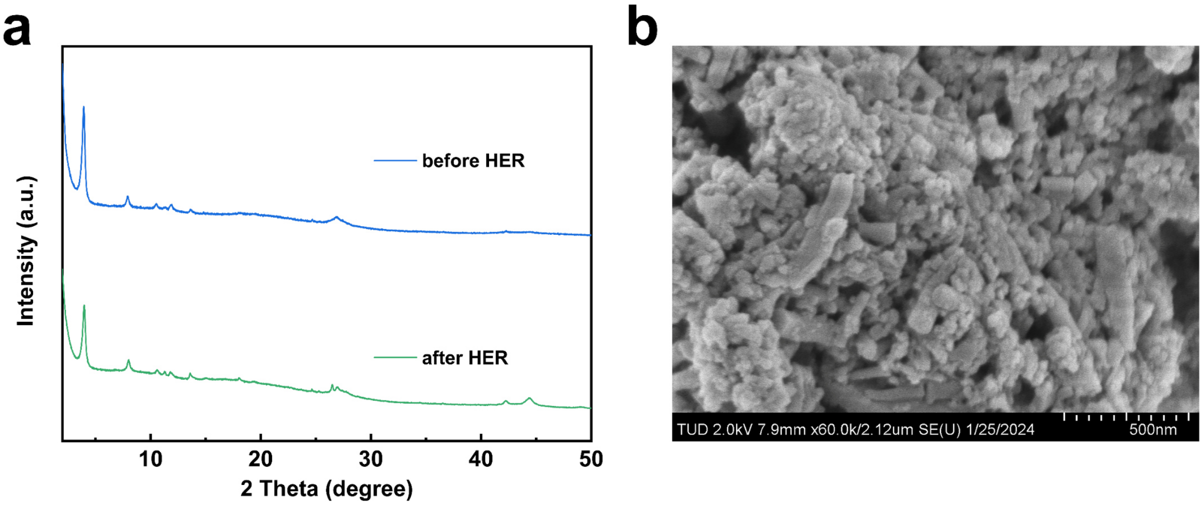

Figure S30. (a) PXRD patterns of $Ni_3(HHTQ)_2$ before and after HER test. (b) SEM images of $Ni_3(HHTQ)_2$ after HER test.

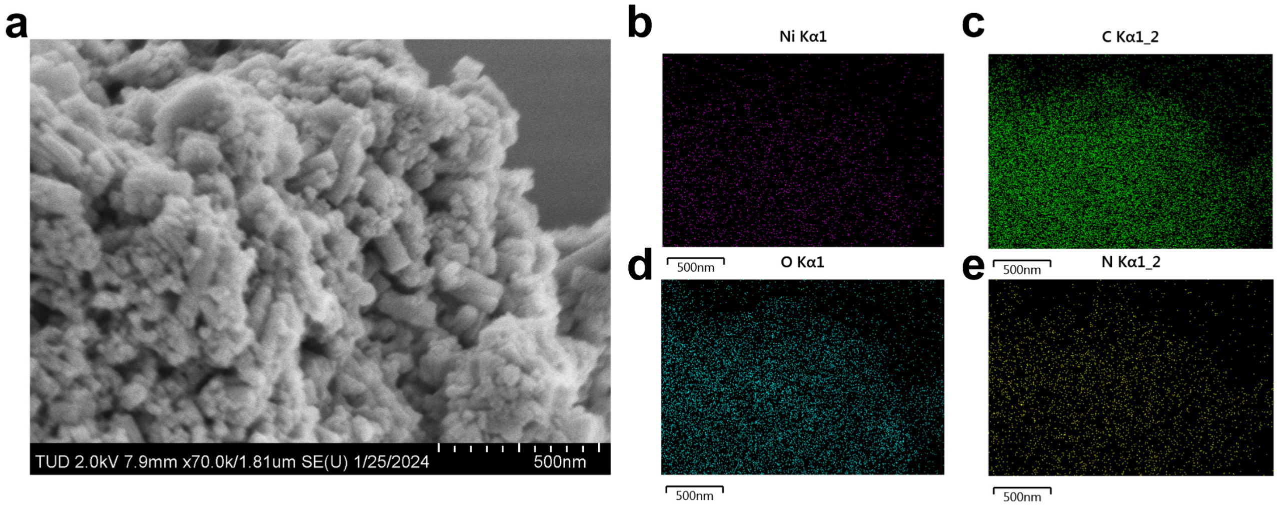

Figure S31. EDS mappings of $Ni_3(HHTQ)_2$ after HER test. (a) SEM image, and (b-e) corresponding EDS mapping images of $Ni_3(HHTQ)_2$ with respect to Ni, C, O, N.

16. *In situ-^{13}C electrochemical (EC) -NMR spectroscopy* **I*n situ*-^{13}C EC-NMR**

In situ-^{13}C EC-NMR measurement was carried out on the Bruker Avance 300 MHz spectrometer with a commercial *in situ* NMR probe (eProbe GmbH, Erfurt, Germany). Special low pass filters were used between electrodes and potentiostat to minimize interference between RF pulse and potentiometric measurement.[1] For the electrochemical measurements, a potentiostat (Iviumstat) is connected to the NMR probehead. For the *in situ*-^{13}C EC-NMR measurement, 3D printed cell with a diameter of 2.2 cm was used (Figure S32). The water solution of 1 M KOH and 0.1 M ^{13}C labelled glycerol $(HOCH_2)_2{}^{13}CHOH$ (Sigma Aldrich) was utilized as the electrolyte. The electrochemical system is composed of three electrodes, using catalyst-modified carbon paper as the working electrode, pure carbon paper as the counter electrode, and a home-made leakless, bipolar reference electrode (BPRE) based on Ag/AgCl as the reference electrode,[2] respectively. The home build Ag/AgCl electrode was tested by open circuit voltage (OCV) before *in situ*-^{13}C EC-NMR measurement to ensure the good electrochemical stability of the electrode. *In situ*-^{13}C NMR is performed with single-pulse excitation at a resonance frequency of 75.47 MHz. *In situ*-^{13}C NMR spectrum is accumulated by 1342 scans with a 45° pulse length of 11.25 μs and a delay time of 4 s equivalent to T_1. A pseudo 2D single pulse sequence was used for the recording of the *in situ*-^{13}C NMR spectra. All the NMR data was processed by Topspin software. The *in situ*-^{13}C EC-NMR measurements were performed in the 3D printed cell at different applied oxidation potentials of 1.30, 1.50, and 1.55 V for 80 h. The ^{13}C chemical shift was referenced with respect to the signal of a standard ethanol solution.

The $^{13}C_2$-labeled glycerol was used for *in situ*-^{13}C EC-NMR (Figure S34), so only compounds with ^{13}C-labeled atoms could be identified. The detailed elucidation is presented in Figure S34. As we have mentioned in the manuscript and supporting information, a glycerol molecule, after complete electrochemical oxidation, can break two C-C single bonds and generate three formate ions. Only $^{13}C_2$-labeled formate can be detected, while the other two formate ions with unlabeled C atoms (C_1 and C_3) cannot be detected. However, in ^{1}H NMR spectroscopy, all three formate molecules produced by electrochemical oxidation of one glycerol molecule can be identified. Therefore, in the

concentration profiles from *in situ*-^{13}C EC-NMR, the concentration of formate product is not the highest at some applied oxidation potentials.

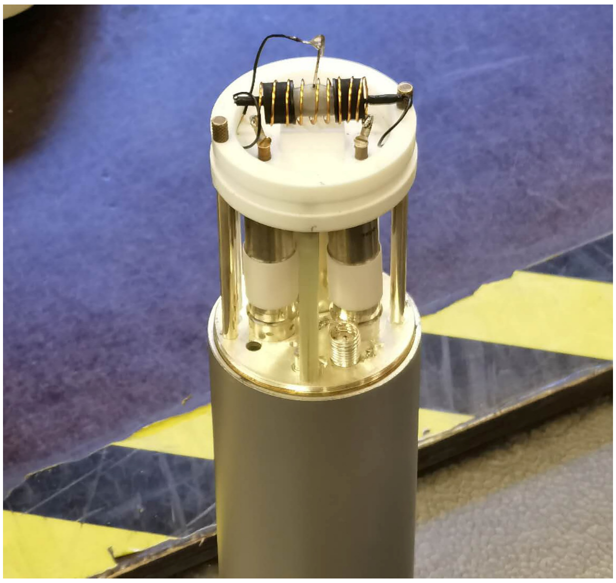

Figure S32. The customized 3D-printed cell used for *in situ*-^{13}C EC-NMR measurement.

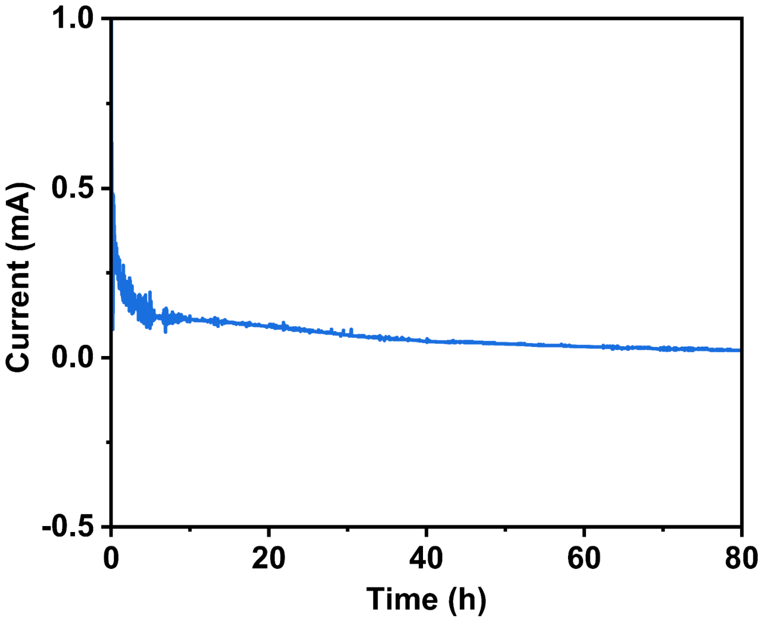

Figure S33. Chronoamperometry (CA) curve for *in situ* ^{13}C EC-NMR at the applied oxidation potential of 1.50 V (vs. RHE) during the 80 h measurement.

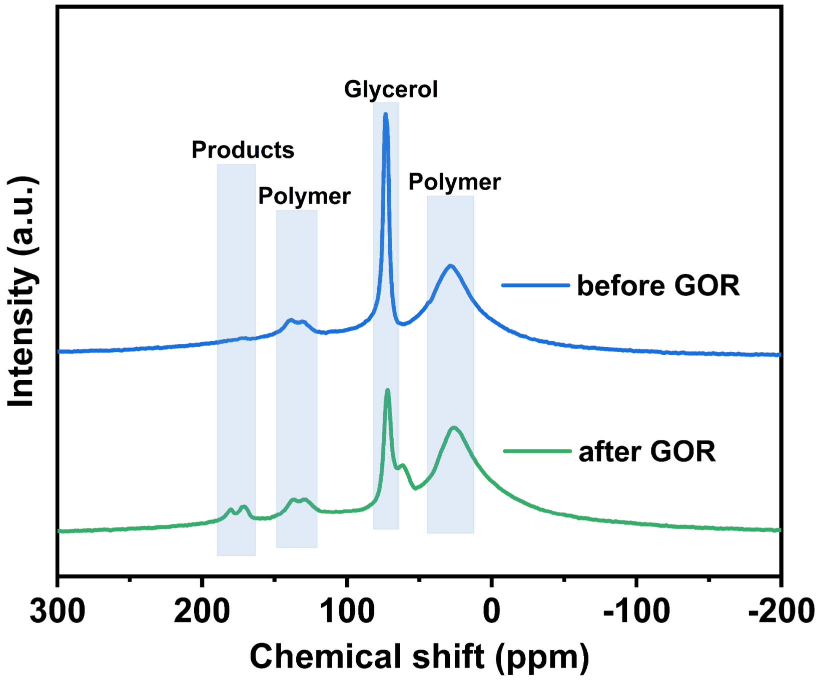

Figure S34. ^{13}C NMR spectra before and after GOR for products, polymer material from *in situ* EC-cell and glycerol.

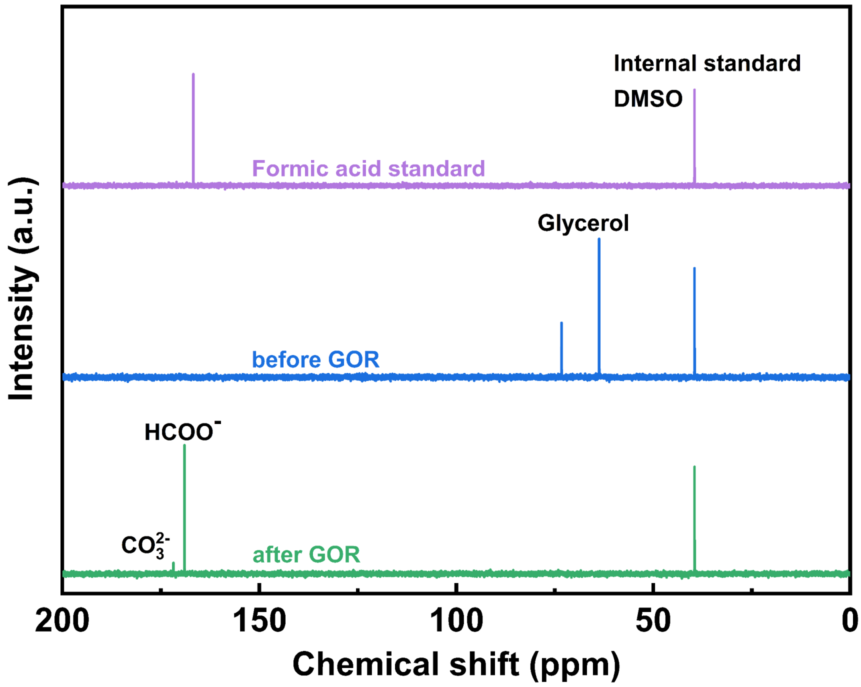

Figure S35. ^{13}C NMR spectra of products before and after 50 h GOR on $Ni_3(HHTQ)_2$.

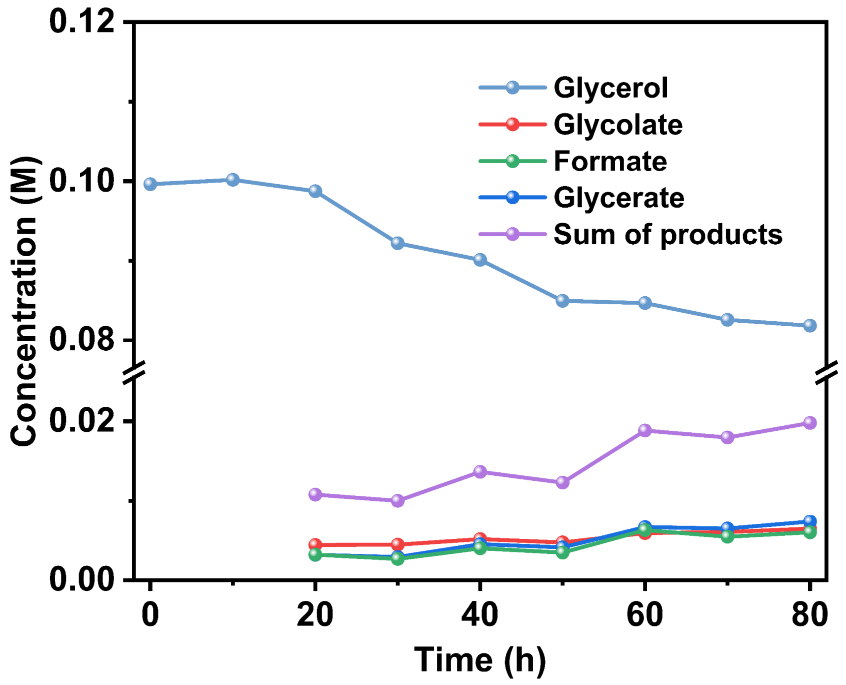

Figure S36. Concentration profiles of GOR compounds on as-prepared $Ni_3(HHTQ)_2$ at potential of 1.30 V, in 1.0 M KOH with 0.1 M ^{13}C-labeled glycerol.

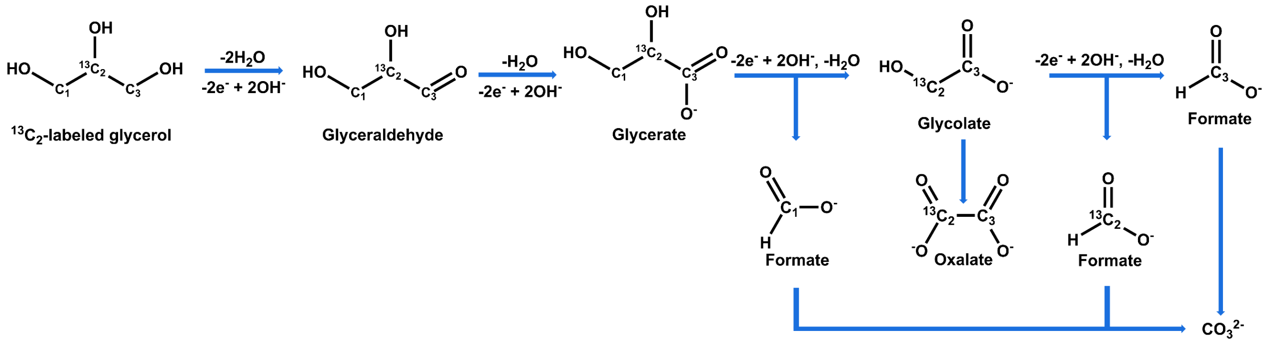

Figure S37. Illustration of the reaction pathway for GOR based on *in situ*-^{13}C EC-NMR.

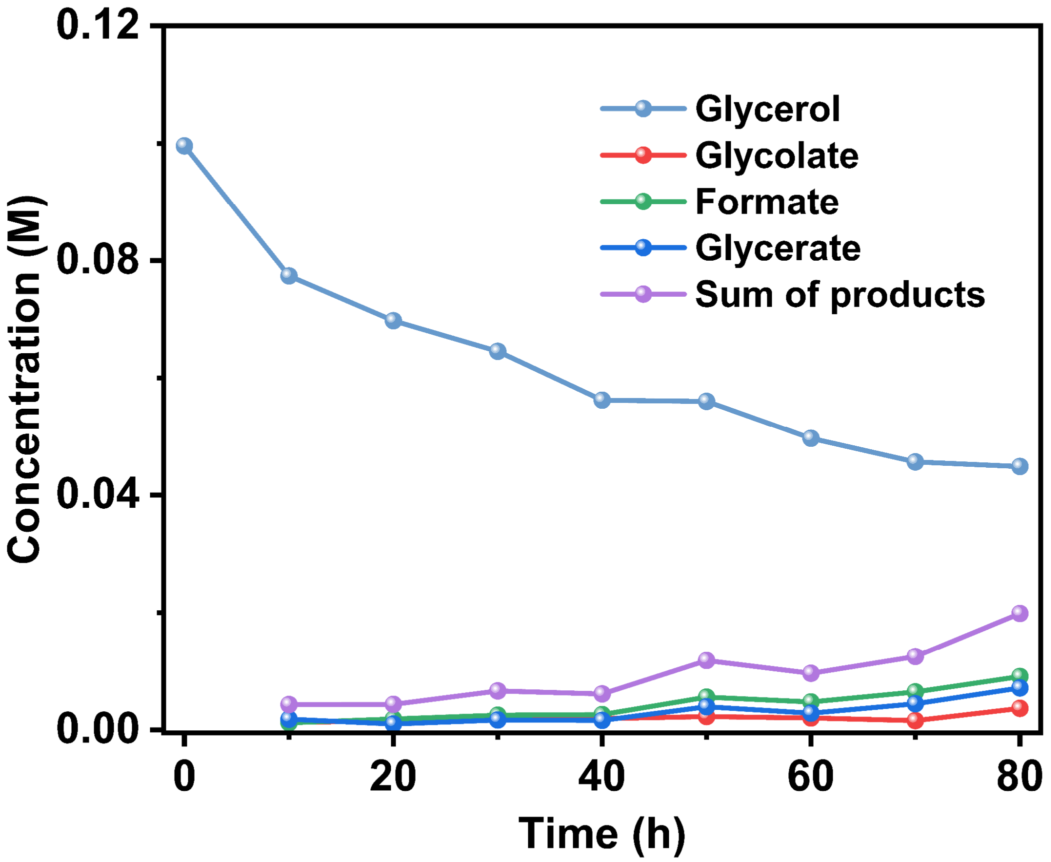

Figure S38. Concentration profiles of GOR compounds on as-prepared $Ni_3(HHTQ)_2$ at a potential of 1.50 V, in 1.0 M KOH with 0.1 M ^{13}C-labeled glycerol.

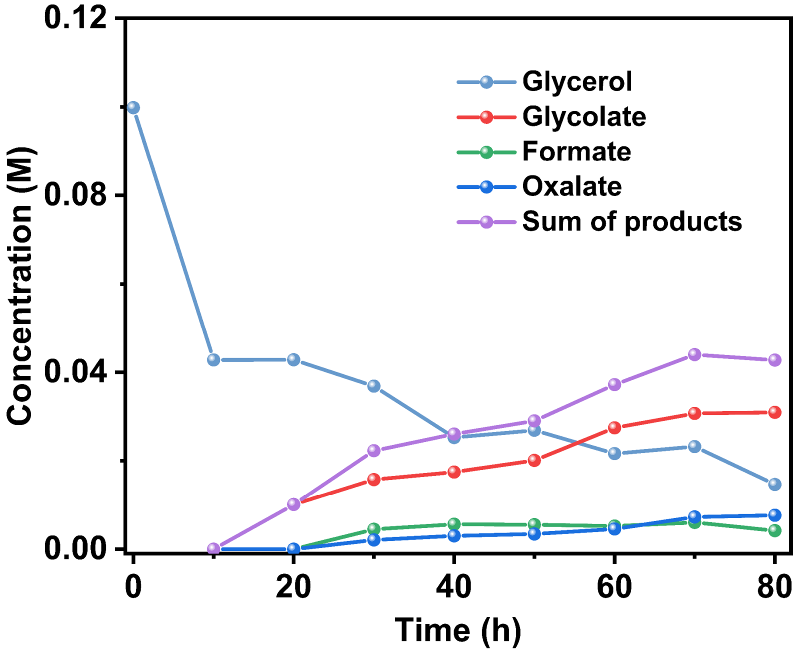

Figure S39. Concentration profiles of GOR compounds on as-prepared $Ni_3(HHTQ)_2$ at a potential of 1.55 V, in 1.0 M KOH with 0.1 M ^{13}C-labeled glycerol.

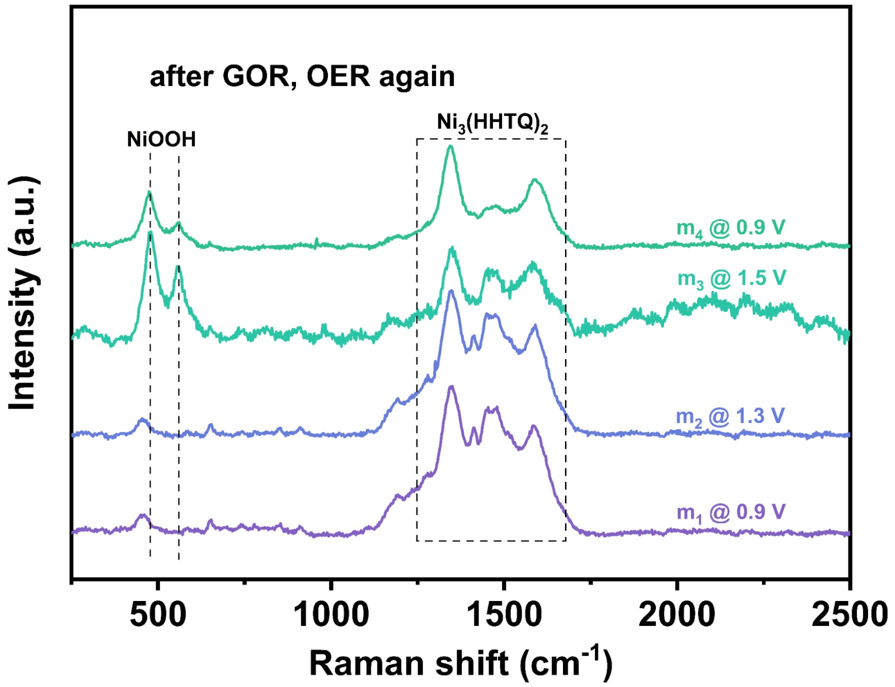

Figure S40. *In situ* EC-Raman spectra of $Ni_3(HHTQ)_2$ after GOR and measured OER again under different applied potentials.

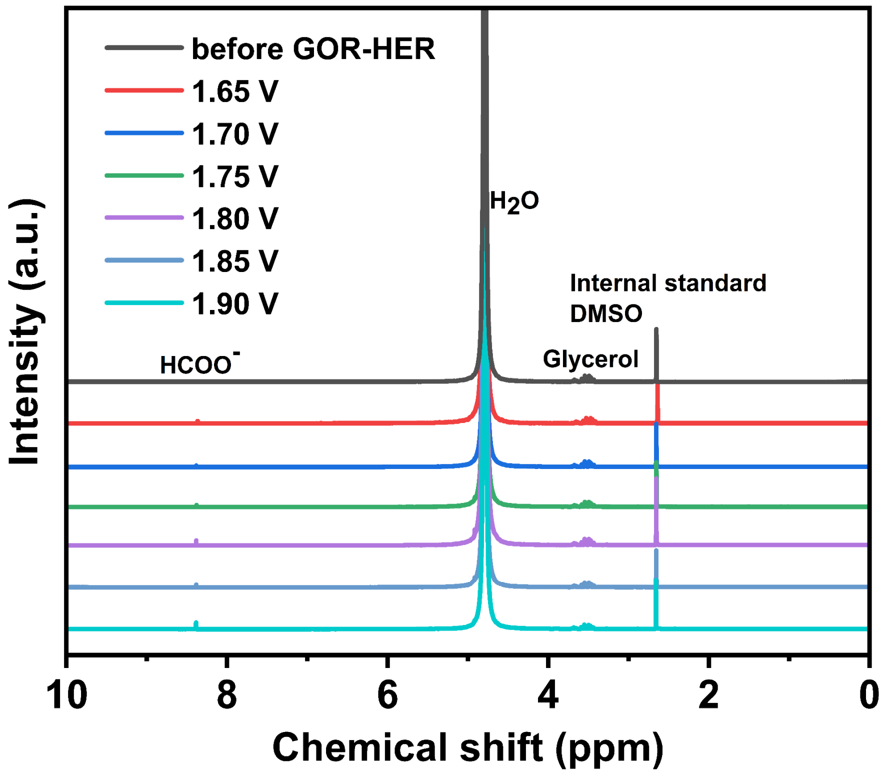

Figure S41. ^1H NMR spectra of the electrolytes after 8 h in a two-electrode system at various applied oxidation voltages.

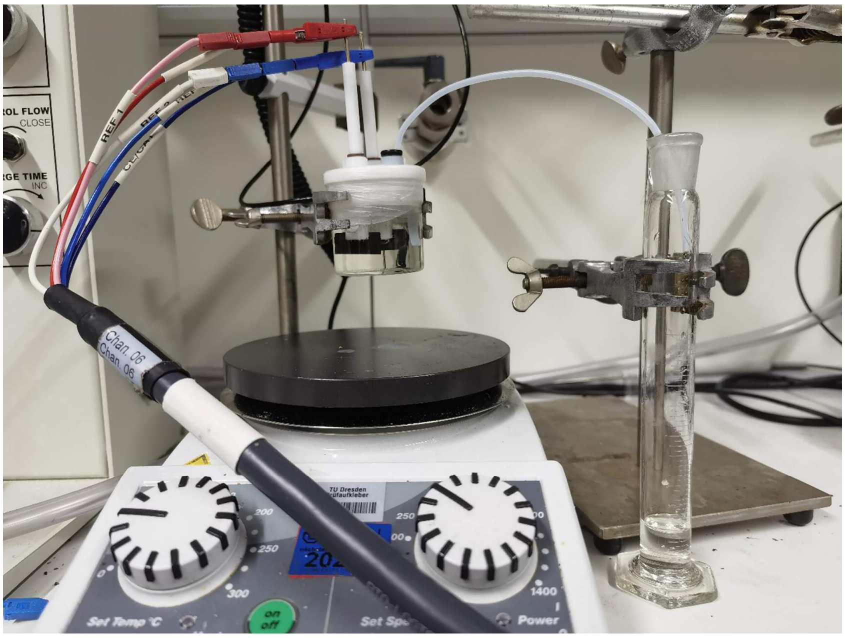

Figure S42. Photographic image of the drainage device for hydrogen production.

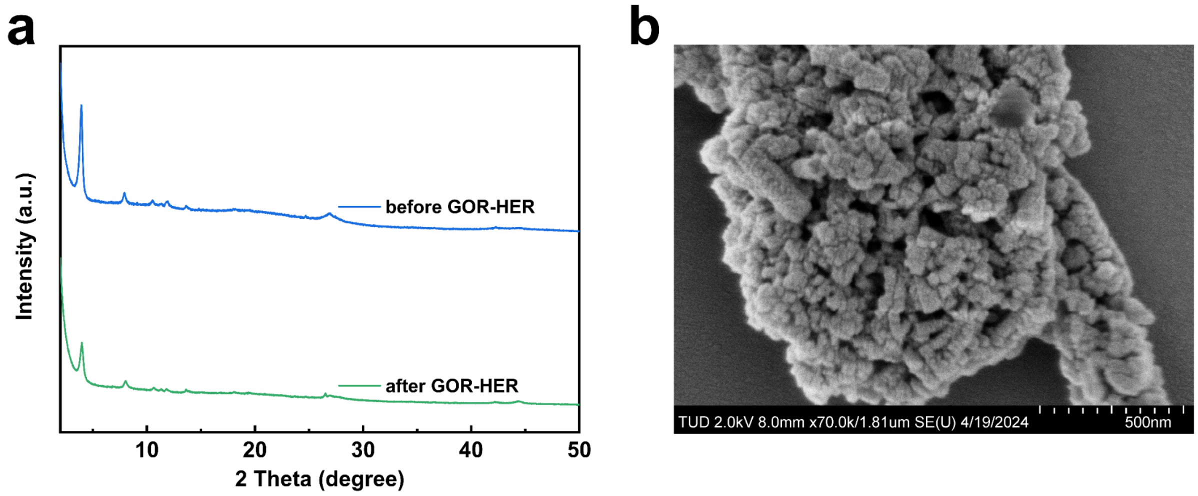

Figure S43. (a) PXRD patterns of $Ni_3(HHTQ)_2$ before and after long-term GOR-HER test. (b) SEM images of $Ni_3(HHTQ)_2$ after long-term GOR-HER test.

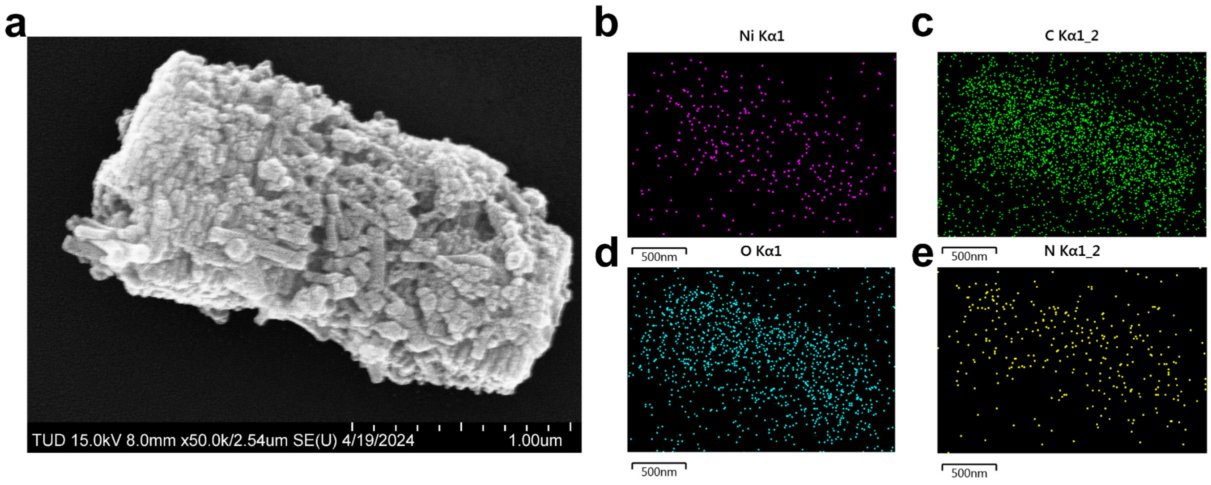

Figure S44. EDS mappings of $Ni_3(HHTQ)_2$ after GOR-HER test. (a) SEM image, and (b-e) corresponding EDS mapping images of $Ni_3(HHTQ)_2$ with respect to Ni, C, O, N.

17. Computational details

The density functional theory (DFT) calculations were performed on single unit cells under periodic boundary conditions (PBC) with the length of the *c*-axis set to four times. The cells were constructed from related crystallographic data.[3-4] In addition, a 1 x 1 x 4 supercell structure was geometrically optimized for each MOF using Kohn-Sham DFT (KS-DFT). The adsorptive molecules were geometrically optimized using KS-DFT. The cell size for the organic molecules was set to the smallest 1 x 1 x 4 MOF supercell.

The calculations were performed on one isolated MOF layer, terminated by vacuum conditions. The geometries of the single layer MOF, as well as the adsorbed complex (one molecule of adsorbate added to the MOF), were optimized with the extended Tight Binding ($GFN_{1-x}TB$) method reported by Grimme et al.,[9] which is included in the quickstep (QS) module in CP2K (Version 2024.1). The energies were calculated using KS-DFT on the optimized geometries. The Gaussian and plane waves method[10] was employed with Goedecker-Teter-Hutter pseudopotentials-Perdew-Burke-Ernzerhof exchange-correlation functional (GTH-PBE) potentials[11] and double zeta valence plus polarization-molecular optimized-GTH (DZVP-MOLOPT-GTH) basis sets.[12] The basis set for the nickel was set to short range. The exchange-correlation (XC) potential was set to the PBE potential and Grimme's DFTD3 correction.[13]

The adsorption energies E_{Ads} were calculated as:

$$E_{\mathrm{Ads}} = E_{A-MOF} - (\mathrm{E_A} + \mathrm{E_{MOF}}),$$

where $E_{A\text{-}MOF}$ is the energy of the adsorbent-adsorbate-complex, E_{MOF} is the energy of the adsorbent MOF without the adsorbed molecule, and E_A is the energy of the free adsorbate.

The Gibbs free energies were calculated as the change in energies upon adsorption of all molecules, starting from the substrate, including intermediates, and the corresponding product molecules. The energies were further adjusted by the number of exchanged electrons and an applied potential of 1.3 V.

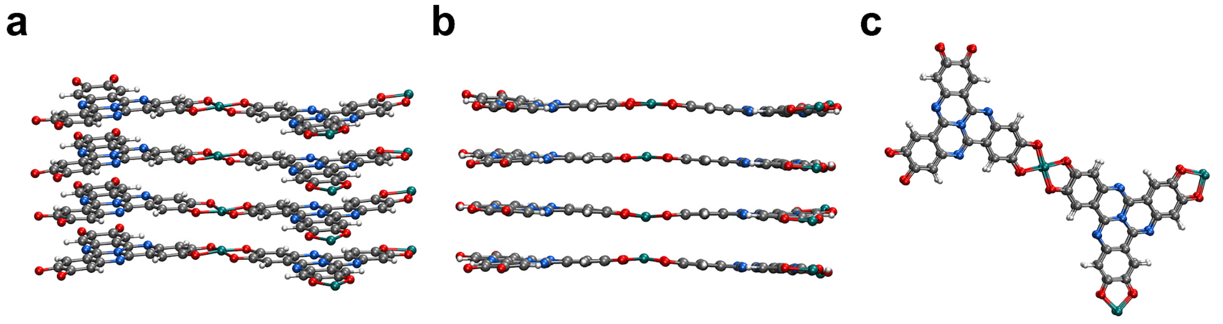

Figure S45. The DFT-optimized structure of $Ni_3(HHTQ)_2$: Multilayers for (a) 3D view, (b) side view, and (c) top view.

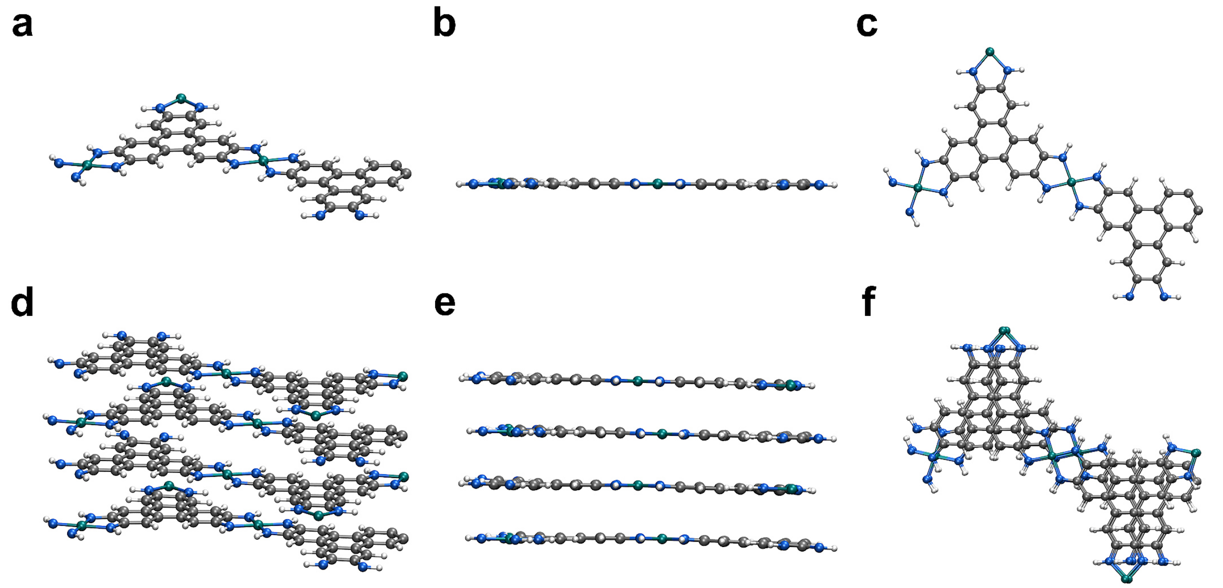

Figure S46. The DFT-optimized structure of $Ni_3(HITP)_2$. single layer and multilayers for (a) and (d) 3D view, (b) and (e) side view, and (c) and (f) top view.

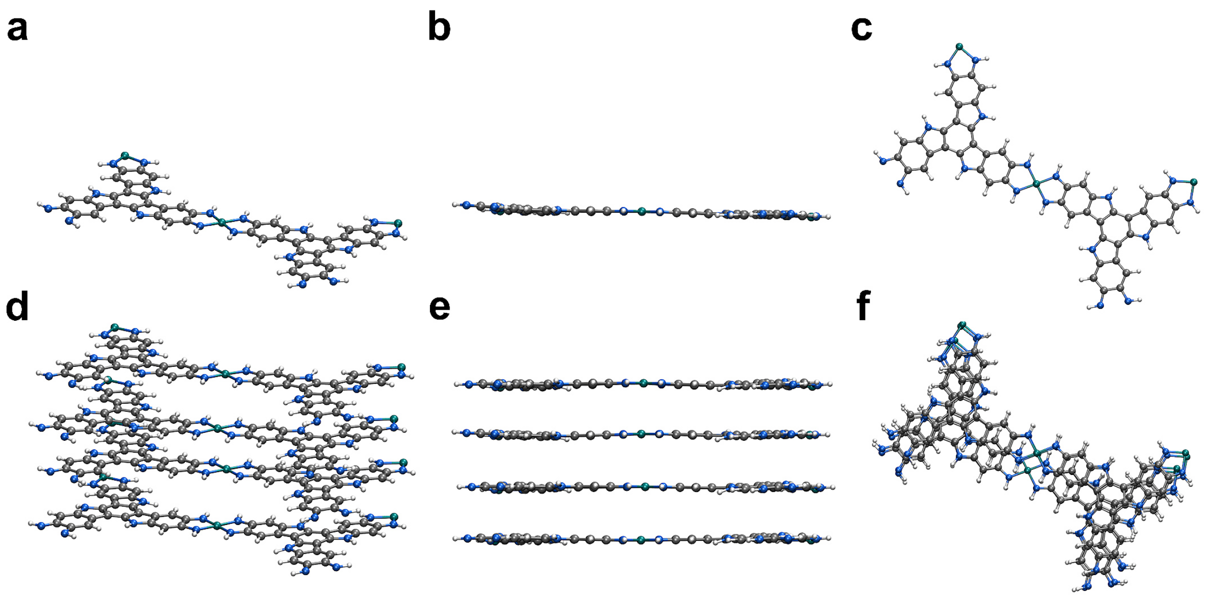

Figure S47. The DFT-optimized structure of $Ni_3(HATI)_2$. Single layer and multilayers for (a) and (d) 3D view, (b) and (e) side view, and (c) and (f) top view.

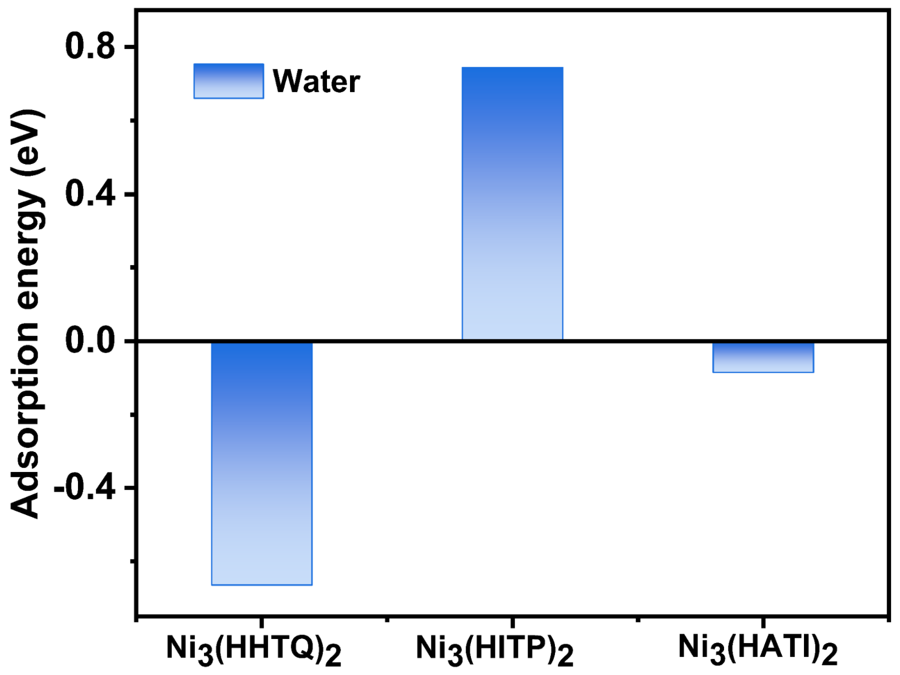

Figure S48. Adsorption free energy of water on Ni-X_4 sites in $Ni_3(HHTQ)_2$, $Ni_3(HITP)_2$, and $Ni_3(HATI)_2$, respectively.

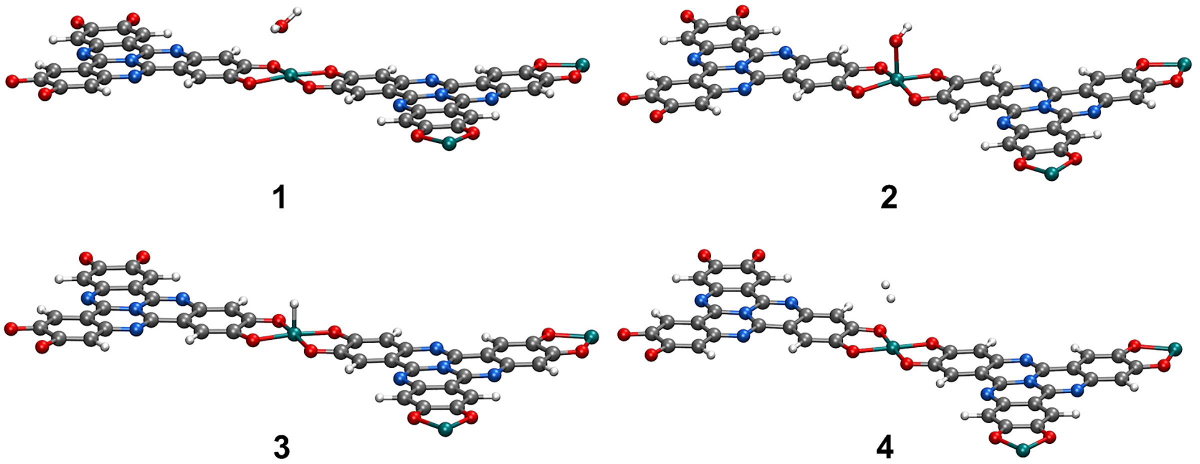

Figure S49. The HER process for $Ni_3(HHTQ)_2$. 1 - one water molecule, 2 - one hydroxide ion, 3 - one hydrogen atom, and 4 - one hydrogen molecule.

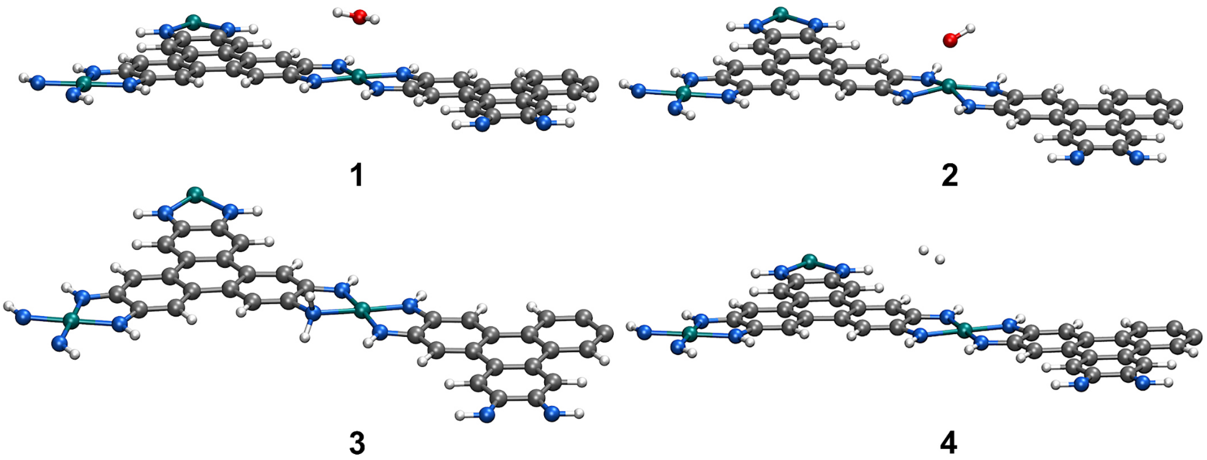

Figure S50. The HER process for $Ni_3(HITP)_2$. 1 - one water molecule, 2 - one hydroxide ion, 3 - one hydrogen atom, and 4 - one hydrogen molecule.

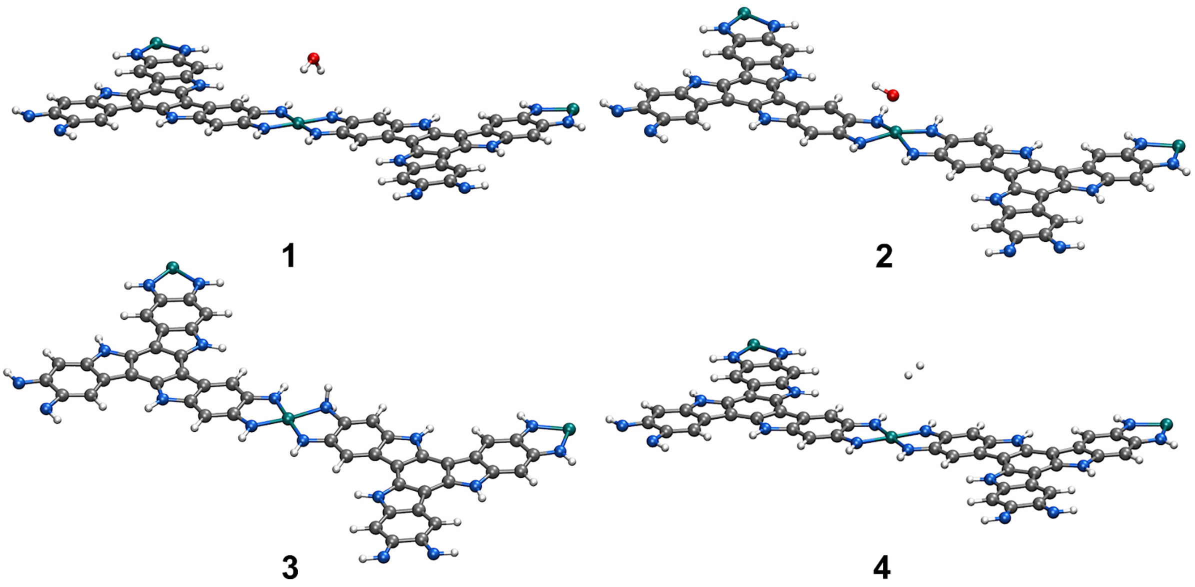

Figure S51. The HER process for $Ni_3(HATI)_2$. 1 - one water molecule, 2 - one hydroxide ion, 3 - one hydrogen atom, and 4 - one hydrogen molecule.

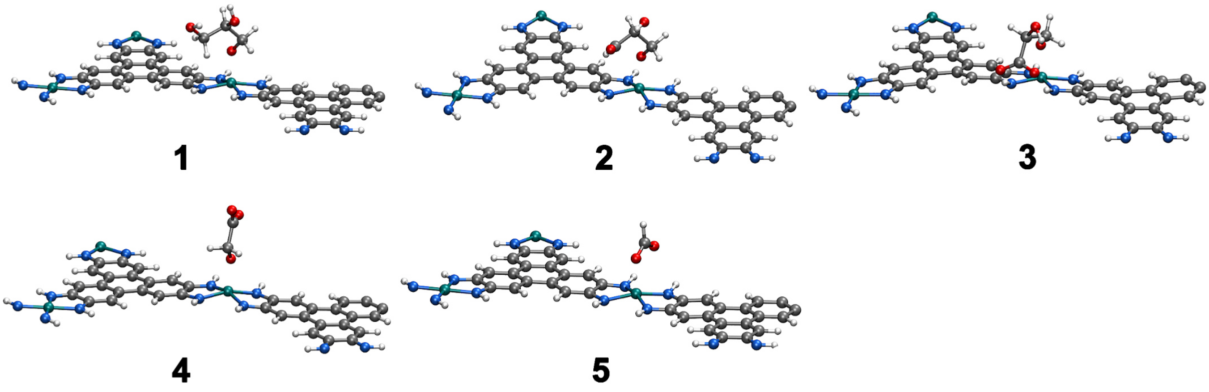

Figure S52. The GOR process for $Ni_3(HITP)_2$. 1 - one glycerol molecule, 2 - one glyceraldehyde molecule, 3 - one glycerate molecule, 4 - one glycolate molecule, 5 - one formate molecule.

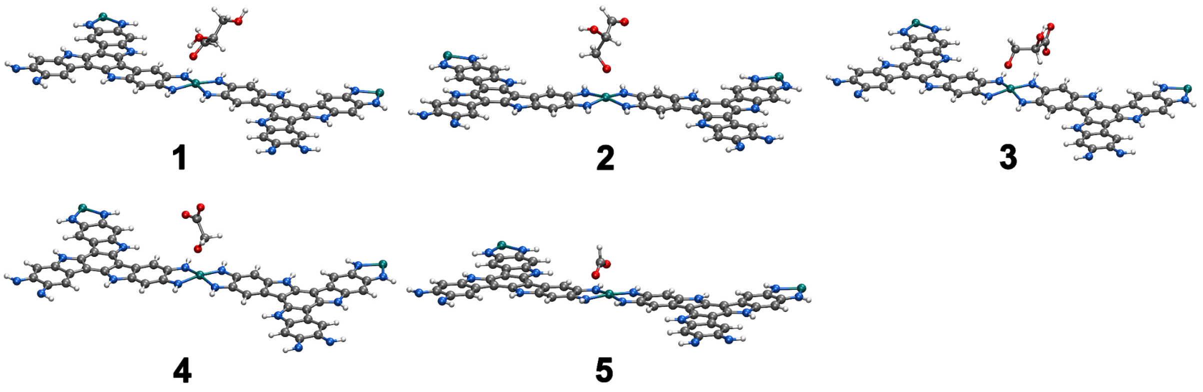

Figure S53. The GOR process for $Ni_3(HATI)_2$. 1 - one glycerol molecule, 2 - one glyceraldehyde molecule, 3 - one glycerate molecule, 4 - one glycolate molecule, 5 - one formate molecule.

Table S6. The Adsorption energies calculated for *c*-MOFs.

Samples	Adsorption energy of water (eV)	Adsorption energy of glycerol (eV)	Adsorption energy of OH^- (eV)
$Ni_3(HHTQ)_2$	-0.66	-2.77	-4.49
$Ni_3(HITP)_2$	0.74	-0.79	-2.60
$Ni_3(HATI)_2$	-0.08	0.46	-1.87

Table S7. Performance comparisons of the electrochemical biomass reforming systems in alkaline solutions.

Catalyst	Electrolyte	Value-added products	Potential at 10 mA cm^{-2} (V vs. RHE)	Ref
$Ni_3(HHTQ)_2$	1 M KOH +0.1 M glycerol	formate	1.36	This work
NF/NiMoO-Ar	1 M KOH+0.5 M urea	N_2, CO_2	1.37	[14]
Co_3O_4 NWs/CC	1 M KOH+40 mg L^{-1} triclosan	Phenol	1.54	[15]
$Co(OH)_2$@HOS/CP	1 M KOH +2 M methanol	formate	1.397	[16]
Ni-MOF/NF	1 M KOH +4 M methanol	formate	1.39	[17]
Co-S-P/CC	1 M KOH +1 M ethanol	acetic acid	1.38	[18]
CoNi-PHNs	1 M KOH +1 M ethanol	acetic acid	1.39	[19]
Cu-oxide	0.1 M KOH+0.1 M glycerol	glyceric acid	1.41	[20]
$Co(OH)_2$&Co+3O(OH)Ti	1 M KOH+0.3 M urea	N_2, CO_2	1.536	[21]
CuCo-UMOFNs	1 M KOH+3 M methanol	-	1.365	[22]
CoS_2 NA/Ti	1 M KOH+0.3 M urea	N_2, CO_2	1.4	[23]

References

[1] K. Kitada, O. Pecher, P. Magusin, M. F. Groh, R. S. Weatherup, C. P. Grey, *J. Am Chem. Soc.* **2019**, 141, 7014-7027.

[2] N. L. Walker, J. E. Dick, *Anal. Chem.* **2021**, 93, 10065-10074.

[3] J. H. Dou, M. Q. Arguilla, Y. Luo, J. Li, W. Zhang, L. Sun, J. L. Mancuso, L. Yang, T. Chen, L. R. Parent, G. Skorupskii, N. J. Libretto, C. Sun, M. C. Yang, P. V. Dip, E. J. Brignole, J. T. Miller, J. Kong, C. H. Hendon, J. Sun, M. Dinca, *Nat. Mater.* **2021**, 20, 222-228.

[4] Y. Lu, Z. Hu, P. Petkov, S. Fu, H. Qi, C. Huang, Y. Liu, X. Huang, M. Wang, P. Zhang, U. Kaiser, M. Bonn, H. I. Wang, P. Samori, E. Coronado, R. Dong, X. Feng, *J. Am Chem. Soc.* **2024**, 146, 2574-2582.

[5] J. Rodríguez-Carvajal, *Physica B* **1993**, 192, 55-69.

[6] M. Hmadeh, Z. Lu, Z. Liu, F. Gándara, H. Furukawa, S. Wan, V. Augustyn, R. Chang, L. Liao, F. Zhou, E. Perre, V. Ozolins, K. Suenaga, X. Duan, B. Dunn, Y. Yamamto, O. Terasaki, O. M. Yaghi, *Chem. Mater.* **2012**, 24, 3511-3513.

[7] K. Kawashima, R. A. Márquez, Y. J. Son, C. Guo, R. R. Vaidyula, L. A. Smith, C. E. Chukwuneke, C. B. Mullins, *ACS Catal.* **2023**, 13, 1893-1898.

[8] a) X. Han, N. Li, Y. B. Kang, Q. Dou, P. Xiong, Q. Liu, J. Y. Lee, L. Dai, H. S. Park, *ACS Energy Lett.* **2021**, 6, 2460-2468; b) L.-L. Feng, G. Yu, Y. Wu, G.-D. Li, H. Li, Y. Sun, T. Asefa, W. Chen, X. Zou, *J. Am Chem. Soc.* **2015**, 137, 14023-14026; c) P. Cai, Y. Li, G. Wang, Z. Wen, *Angew. Chem. Int. Ed.* **2018**, 57, 3910-3915; d) Z. J. Chen, J. Dong, J. Wu, Q. Shao, N. Luo, M. Xu, Y. Sun, Y. Tang, J. Peng, H. M. Cheng, *Nat. Commun.* **2023**, 14, 4210.

[9] S. Grimme, C. Bannwarth, P. Shushkov, *J. Chem. Theory Comput.* **2017**, 13, 1989-2009.

[10] a) G. Lippert, J. Hutter, M. Parrinello, *Mol. Phys.* **2010**, 92, 477-488; b) T. D. Kühne et al., *J. Chem. Phys.* **2020**, 152, 194103.

[11] a) S. Goedecker, M. Teter, J. Hutter, *Phys. Rev. B* **1996**, 54, 1703-1710; b) C. Hartwigsen, S. Goedecker, J. Hutter, *Phys. Rev. B* **1998**, 58, 3641-3662; c) M. Krack, *Theor. Chem. Acc.* **2005**, 114, 145-152.

[12] J. VandeVondele, J. Hutter, *J. Chem. Phys.* **2007**, 127, 114105.

[13] S. Grimme, J. Antony, S. Ehrlich, H. Krieg, *J. Chem. Phys.* **2010**, 132, 154104.

[14] Z.-Y. Yu, C.-C. Lang, M.-R. Gao, Y. Chen, Q.-Q. Fu, Y. Duan, S.-H. Yu, *Energ. & Environ. Sci.* **2018**, 11, 1890-1897.

[15] C. Lyu, J. Zheng, R. Zhang, R. Zou, B. Liu, W. Zhou, *Mater. Chem. Front.* **2018**, 2, 323-330.

[16] K. Xiang, D. Wu, X. Deng, M. Li, S. Chen, P. Hao, X. Guo, J. L. Luo, X. Z. Fu, *Adv. Funct. Mater.* **2020**, 30, 1909610.

[17] Y. Xu, M. Liu, M. Wang, T. Ren, K. Ren, Z. Wang, X. Li, L. Wang, H. Wang, *Appl. Catal. B-Environ.* **2022**, 300, 120753.

[18] S. Sheng, K. Ye, L. Sha, K. Zhu, Y. Gao, J. Yan, G. Wang, D. Cao, *Inorg. Chem. Front.* **2020**, 7, 4498-4506.

[19] W. Wang, Y. B. Zhu, Q. Wen, Y. Wang, J. Xia, C. Li, M. W. Chen, Y. Liu, H. Li, H. A. Wu, T. Zhai, *Adv. Mater.* **2019**, 31, 1900528.

[20] L. S. Oh, M. Park, Y. S. Park, Y. Kim, W. Yoon, J. Hwang, E. Lim, J. H. Park, S. M. Choi, M. H. Seo, W. B. Kim, H. J. Kim, *Adv. Mater.* **2023**, 35, 2203285.

[21] Y. Jiang, S. Gao, J. Liu, G. Xu, Q. Jia, F. Chen, X. Song, *Nanoscale* **2020**, 12, 11573-11581.

[22] X. Wei, S. Wang, Z. Hua, L. Chen, J. Shi, *ACS Appl. Mater. Interfaces* **2018**, 10, 25422-25428.

[23] S. Wei, X. Wang, J. Wang, X. Sun, L. Cui, W. Yang, Y. Zheng, J. Liu, *Electrochim. Acta* **2017**, 246, 776-782.
